# Single-cell morphodynamics predict cell fate decisions during mucociliary epithelial differentiation

**DOI:** 10.1038/s44320-026-00212-x

**Published:** 2026-05-11

**Authors:** Mari Tolonen, Ziwei Xu, Ozgur Beker, Varun Kapoor, Bianca Dumitrascu, Jakub Sedzinski

**Affiliations:** 1 The Novo Nordisk Foundation Center for Stem Cell Medicine (reNEW), University of Copenhagen, Copenhagen, Denmark; 2 Department of Biomedical Sciences, University of Copenhagen, Copenhagen, Denmark; 3 Department of Statistics, Columbia University, New York, NY USA; 4 Irving Institute for Cancer Dynamics, Columbia University, New York, NY USA; 5 Kapoorlabs, Paris, France; 6 Universität Osnabrück, Osnabrück, Germany; 7 Department of Computer Science, Columbia University, New York, NY USA; 8 Columbia Stem Cell Institute, Columbia University, New York, NY USA

**Keywords:** Computational Biology, Development

## Abstract

Cell state transitions underlie the emergence of diverse cell types and are traditionally defined by changes in gene expression. Yet these transitions also involve coordinated shifts in cell morphology and behavior, which remain poorly characterized in densely packed epithelia. We developed a quantitative live-imaging and computational framework to track thousands of individual cells over time in the rapidly differentiating *Xenopus* mucociliary epithelium (MCE). From segmentations and trajectories, we extracted dynamic features—cell and nuclear shape, movement, and position—to create a time-resolved morphodynamic dataset spanning the full course of differentiation. While single features showed high noise and low separability of ground-truth cell types, supervised machine learning revealed that integrating time-resolved features improves the prediction of final cell fate. Gradient-boosted trees and multinomial logistic regression achieved moderate but consistent accuracy, especially for abundant epithelial lineages. Key discriminants included normalized Z position, membrane–nucleus offset, and absolute experimental time, whereas movement contributed minimally to the results. Our data show that morphodynamic signatures encode predictive information about cell identity and provide a framework linking cellular dynamics with molecular state.

## Synopsis

Cell and nuclear geometry progressively encode lineage identity during mucociliary epithelial differentiation.

- Morphodynamic profiling reveals gradual, multidimensional changes in cell and nuclear shape across development.
- Unsupervised analyses show weak clustering, indicating that fate is not defined by discrete morphological states.
- Supervised models uncover predictive geometric signatures embedded within time-resolved trajectories.
- Nuclear–membrane spatial organization and 3D shape descriptors outperform motility features in fate prediction.

## Abstract (web summary)

Cell and nuclear geometry progressively encode lineage identity during mucociliary epithelial differentiation.

## Graphical abstract

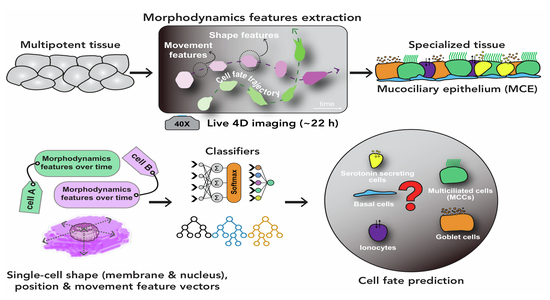

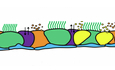

## Introduction

Multicellular organisms are composed of a diverse array of specialized cell types, each defined by a distinct phenotype that integrates a specific molecular program with characteristic cell shape and dynamic behaviors, such as movement and cell–cell interactions. Collectively, these features determine the cell’s functional identity. This integrated profile is often described as the cell state, representing the dynamic manifestation of phenotype and function at a given time (Mulas et al, 2021; Rafelski and Theriot, 2024). Cell states are inherently plastic and context-dependent. Even within homeostatic tissues, individual cells can transition between proliferative and quiescent states or modulate their behavior in response to mechanical stimuli or intercellular signaling (Martino et al, 2018; Marescal and Cheeseman, 2020; Puech and Bongrand, 2021). These dynamic cell state transitions highlight the fluidity of cellular phenotypes and their capacity to adapt to physiological demands.

Among the many biological contexts in which cell state transitions occur, development stands out as a particularly striking example, involving large-scale, tightly regulated shifts in cell identity (Mojtahedi et al, 2016; Pang et al, 2022). During this process, initially pluripotent cells progressively adopt distinct functional roles through coordinated changes in phenotype. Our understanding of these transitions has so far been primarily molecular, driven by advances in single-cell RNA sequencing, which enables high-resolution profiling of gene expression at the level of individual cells. These efforts have revealed distinct cell states, underlying regulatory networks, and lineage hierarchies that shape developmental trajectories (reviewed by, e.g., Griffiths et al, 2018; Sagar and Grün, 2020). Furthermore, with the development of computational methods such as pseudotime and trajectory inference, it has become possible to reconstruct the temporal progression of these transitions from static transcriptomic snapshots (Trapnell et al, 2014; Weinreb et al, 2020; Weiler et al, 2024).

While recent advances in single-cell ‘omics’ technologies, spanning genetic, epigenetic, and chromatin accessibility profiling, have significantly advanced our understanding of cell state transitions and the emergence of cellular phenotypes, much less is known about the accompanying morphogenetic and behavioral changes that occur during development. Morphology and cell behavior are frequently considered the downstream consequences of fate decisions. However, they may serve as complementary, and potentially predictive, indicators of cell state. For example, the stiffness of the extracellular matrix (ECM) can influence cell fate, acting in concert with genetic programs to regulate cellular morphology and function (Guilak et al, 2009; Watt and Huck, 2013; Smith et al, 2018; Luxenburg and Zaidel-Bar, 2019; Hayward et al, 2021). Unlike molecular measurements, cell morphology is continuously quantifiable in both space and time, making it particularly well suited to capture the dynamic and spatially embedded nature of cell state transitions, especially when studied through live imaging (Treiser et al, 2010; Guan et al, 2025; Soelistyo et al, 2022).

Image-based single-cell phenotyping has been widely used in biomedical applications such as drug discovery and medical diagnostics, particularly in cell culture systems, where easily quantifiable phenotypes can be efficiently analyzed at a large scale (Neumann et al, 2010; Rohban et al, 2017; Wiggins et al, 2023; De Vries et al, 2025). However, its application to developmental biology has been limited, largely due to the technical challenges of processing dynamic, high-resolution imaging data in complex living tissues. Accurately segmenting and tracking cells over time in morphogenetically active environments remains a significant obstacle. Recently, the integration of AI-based methods, including deep learning for segmentation, tracking, and feature extraction, has begun to overcome these barriers (Soelistyo et al, 2023; Wen et al, 2021; Kok et al, 2020; Turley et al, 2024; Bragantini et al, 2025b). Combined with the increasing availability of large-scale, single-cell imaging datasets, including whole-embryo recordings, these advances now enable morphodynamic profiling of developmental systems with unprecedented resolution and scale (Shah et al, 2019; Bragantini et al, 2025b). While these studies have begun to relate cellular dynamics to tissue- and organ-level morphogenesis, how cell shape and movement reflect, or even influence, cell-state transitions during development remains largely uncharacterized, particularly in densely packed, multilayered tissues where dynamic morphological features may serve as informative proxies for cell identity and function.

The *Xenopus laevis* mucociliary epithelium (MCE) of the embryonic presumptive epidermis provides a powerful system for dissecting how cell states, lineage decisions, and morphogenetic behaviors unfold within a developing multilayered tissue. This rapidly differentiating epithelium contains multiple specialized cell types, including multiciliated cells (MCCs), goblet cells, ionocytes (ICs), small secretory cells (SSCs), and basal stem cells, arranged in a stereotyped bilayered architecture in the mature tissue, resembling the cellular composition and organization of mammalian airway epithelia (Stubbs et al, 2006; Deblandre et al, 1999; Walentek and Quigley, 2017; Quigley et al, 2011; Walentek et al, 2014; Cibois et al, 2015; Haas et al, 2019; Dubaissi et al, 2014; Bowden et al, 2026; Drysdale and Elinson, 1992; Dubaissi and Papalopulu, 2011; Tríbulo et al, 2012). In our previous work, we defined the lineage relationships within the MCE and characterized the molecular basis of cell lineage bifurcations, demonstrating that distinct cell fates arise through progressive transcriptional transitions from shared progenitor states (Lee et al, 2023).

As these molecular programs unfold, cells also engage in coordinated morphogenetic behaviors that shape the architecture of developing tissues. Goblet cells arise and remain in the superficial (outer) layer, where they acquire a large apical surface early and undergo limited morphogenetic change (Hayes et al, 2007; Billett and Gould, 1971; Huang and Niehrs, 2014). Similarly, basal stem cells remain confined to the basal layer of the epithelium and undergo progressive thinning as the MCE matures (Haas et al, 2019; Cibois et al, 2015). In contrast, MCCs, ICs, and SSCs arise deep within the tissue and execute a sequence of morphogenetic steps as they differentiate. These include vertical cell rearrangements of their progenitors associated with epithelial thinning, epiboly-like cell shifts in both apical and basal directions (Keller, 1980; Marsden and DeSimone, 2001; Luu et al, 2011), and ultimately radial intercalation, during which deep-layer progenitors insert into the superficial layer in a temporally staggered, fate-specific manner (Keller, 1980; Marsden and DeSimone, 2001; Szabó et al, 2016).

Following intercalation, these lineages display strikingly different apical morphologies and dynamics. MCCs undergo a well-characterized program of extensive active apical surface expansion, driven by actin-based pushing forces and mechanical integration into the surrounding epithelium, whereas ICs and SSCs maintain relatively stable, moderate apical domains (Sedzinski et al, 2016; Walentek et al, 2014; Ventura et al, 2022). In addition, immature MCCs exhibit Scf/Kit-dependent active migration and mutual repulsion prior to intercalation, enabling their characteristic even spacing across the epithelium, a behavior not shared by other intercalating lineages (Chuyen et al, 2021).

Together, these studies highlight that differentiation in the MCE is inseparable from the dynamic changes in cell shape, position, and motility that accompany it. The *Xenopus* MCE, therefore, represents an ideal system to investigate how cell-state transitions are encoded not only in transcriptional programs but also in the quantitative morphological trajectories and movement patterns that cells follow as they build a functional tissue.

In this study, we developed a quantitative live-imaging and computational analysis pipeline to track thousands of individual cells over time in the developing *Xenopus* MCE, capturing dynamic changes in cell shape, nuclear shape, cell position, and movement as cells transition from a multipotent progenitor state to their terminal identities. To explore the relationship between single-cell measurements and cell fate, we developed a method to label the five cell types (basal, goblet, ICs, MCCs, and SSCs) after live imaging and to use trajectory information to reconstruct cells’ developmental trajectories prior to specification. We first extracted morphodynamic features from segmentation and tracking data, generating a rich dataset of time-resolved single-cell behaviors spanning the entire differentiation timeline. Despite the biological relevance of the extracted features, individual-cell features showed high variability, resulting in a feature space with low separability between cell types. Unsupervised explorations, such as PCA, revealed limited separation in feature space beyond slight temporal shifts, with the overall distribution of cells remaining largely uniform and intermixed.

To test whether subtle but consistent differences could nonetheless support fate prediction, we trained a supervised XGBoost classifier using endpoint immunostaining and backtracking-derived ground-truth labels. The model achieved moderate but stable performance across 20 independent stratified train–test splits (80/20), with the highest accuracy for the predominant epithelial lineages: basal cells, goblet cells, and MCCs. Class imbalance was addressed by combining undersampling and synthetic oversampling during training. Feature importance analysis revealed that positional and nuclear shape features, especially Z-position and the nucleus–membrane offset, were the most informative, while movement features contributed relatively little to cell-fate prediction, likely due to collective migration within the tissue. Temporal trends in classification uncertainty and accuracy further indicated that morphodynamic signatures of fate emerge progressively during differentiation and often peak before terminal maturation.

Together, our findings show that, while instantaneous morphodynamic features are noisy and weakly separable, supervised models trained on time-integrated cell trajectories can extract predictive signatures of cell identity. This study establishes a framework for using dynamic aspects of cell shape and movement, alongside molecular profiles, as complementary descriptors of cell state transitions in dense, developing tissues.

## Results

### A live-imaging pipeline for quantifying cell shape and movement during mucociliary epithelial (MCE) differentiation

**Figure 1:**
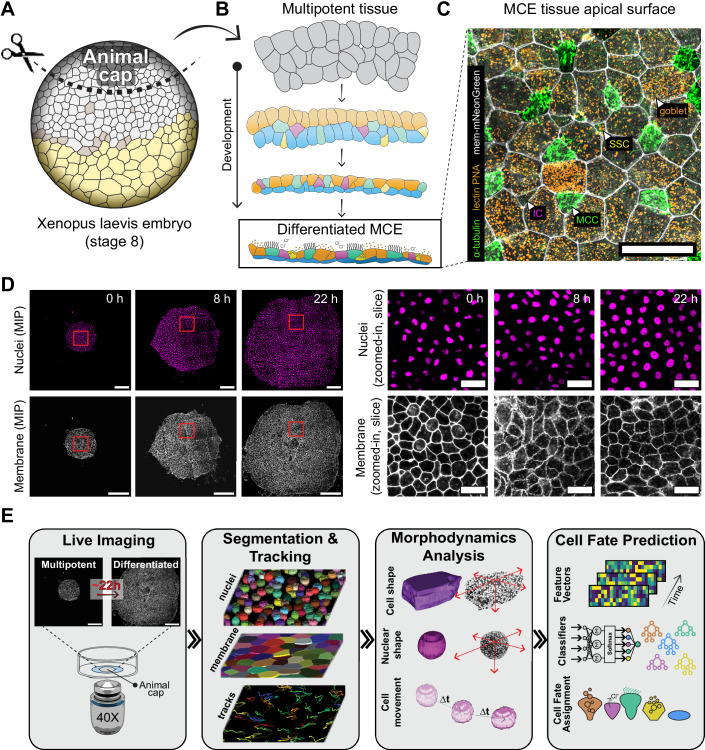
Experimental overview of cell fate prediction. (**A**) Animal caps are cut from Xenopus embryos at NF stages 8–9. (**B**) The prospective MCE undergoes morphogenetic shaping and cell fate decisions during development into differentiated tissue. (**C**) Immunostained differentiated tissue apical surface at NF stage 32. α-tubulin labels multiciliated cells (MCC) (green) and lectin (orange) labels goblet cells, and small secretory cells (SSC) (granular staining). Ionocytes (IC) are distinct for their lack of labeling. Arrowheads and labels mark single cells of each cell type. Scale bar: 50 µm. (**D**) Nuclei and membrane labeling (H2B-RFP and mem-mNeonGreen, respectively) of developing tissue at 0, 8, and 22 h. Overall image scale bar: 300 µm, zoomed-in scale bar: 50 µm. (**E**) Image analysis pipeline from live image acquisition to cell fate prediction. Membrane and nucleus objects are achieved via 3D segmentation, and cell shapes and movement are quantified. Single-cell features are then used to classify cell fate using ground-truth data from cell immunolabeling.

As a first step toward quantifying how dynamic changes in cell morphology and behavior reflect cell-state transitions, we established a high-resolution live-imaging and computational analysis pipeline for the developing multilayered *Xenopus* MCE (Fig. 1). We used animal cap explants, a well-established *Xenopus* system (Dingwell and Smith, 2018) derived from the animal pole ectoderm of the blastula-stage embryo (Fig. 1A). When cultured ex vivo under permissive conditions, this tissue undergoes a well-characterized developmental progression from a multipotent state to terminal differentiation, closely mirroring in vivo epidermal development and recapitulating the key morphogenetic events that structure the tissue during embryogenesis (Fig. 1B). It reproducibly gives rise to the MCE, generating the full spectrum of epithelial cell types, and recapitulating the cell morphologies of the embryonic tissue (Fig. 1C; Appendix Fig. S1).

To visualize individual cells over time, we injected mRNAs encoding membrane- and nucleus-localized fluorescent markers (mem-mNeonGreen and H2B-RFP, respectively) into early-stage embryos. Animal caps were dissected at Nieuwkoop-Faber (NF) stage 8–9, while the tissue was still multipotent, and mounted onto fibronectin-coated glass-bottom dishes. Upon adhesion, the tissue flattened, forming a stratified epithelium that enabled continuous optical access throughout development. Volumetric time-lapse imaging was performed over 16–22 h using confocal microscopy, with z-stacks acquired every 3–5 min. This setup enabled us to capture dynamic cell behaviors throughout differentiation, from multipotent states to terminal identities (NF stages 9–30) (Fig. 1D; Movie EV1). Across three independent experiments, which we refer to as Datasets 1–3, we acquired high-resolution datasets spanning all major developmental timepoints (Appendix Table S1).

For analysis, we implemented a custom image-processing pipeline to extract morphodynamic features at the single-cell level (Fig. 1E). Segmented cells were tracked across time points, and their features—such as shape, size, and movement—were quantified. Using ground-truth cell type annotations derived from endpoint immunostaining, we trained classifiers to infer cell fate from morphodynamic profiles (Fig. 1E). Together, this pipeline enables high-resolution, long-term tracking of individual cells as they transition from multipotent to terminal fates within a differentiating tissue context. By capturing both morphological and behavioral dynamics over time, we can begin to map how distinct cell identities emerge during MCE formation.

### Segmentation and tracking pipeline optimized for developing MCE

**Figure 2:**
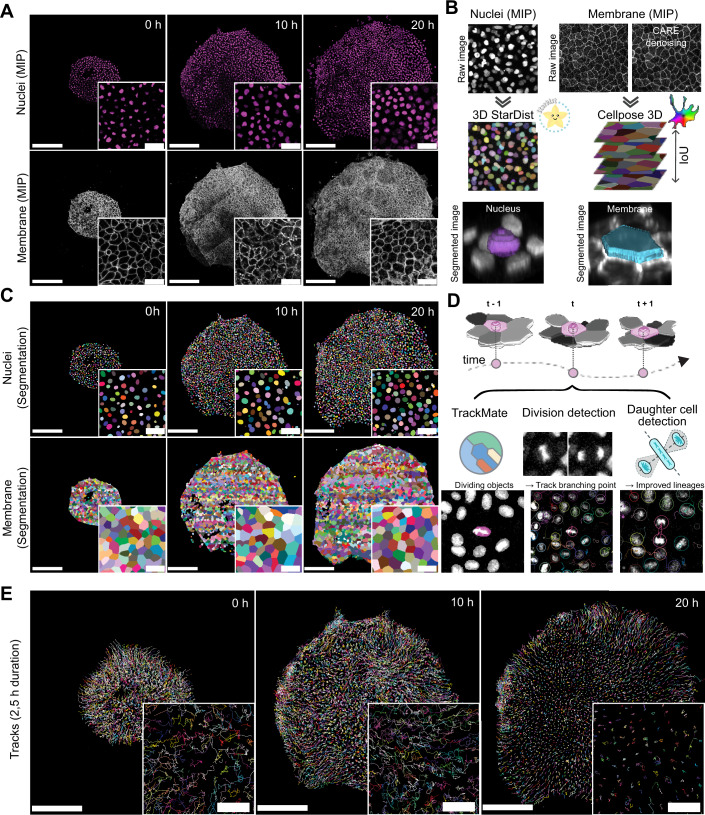
Segmentation and tracking overview. (**A**, **B**) Nuclei and membrane stacks (raw for nuclei, CARE denoised for membrane) are used as inputs for StarDist (nuclei) and Cellpose (membrane). For Cellpose, segmentation is performed slicewise, and 3D objects are reconstructed based on maximal slice-to-slice overlap (IoU). Close-up panels are single z slices (**A**). (**C**) Segmented nuclei and membrane object labels. (**D**) Cell trajectories are generated by tracking nuclei objects, which are then assigned membrane objects based on the closest object centroid matching. Tracks are initially generated with TrackMate, using StarDist-labeled objects as input, and trajectory branching points are imposed at cells classified as dividing by Oneat using the Fiji plugin TrackMate-Oneat. (**E**) Resulting cell trajectories at 0, 10, and 20 h. Scale bars for (**A**, **C**, **E**): overall image 300 µm, close-up 50 µm.

To characterize single-cell morphodynamics during MCE differentiation, we utilized 3D segmentation and tracking of our volumetric imaging data (Fig. 2A). Raw nuclear signals were segmented using a 3D StarDist (Weigert et al, 2020) model trained on manually annotated volumes. Membrane signals were first denoised using CARE (Weigert et al, 2018) and then segmented in 2D on individual z-slices using Cellpose (Stringer et al, 2021; Pachitariu and Stringer, 2022) (Fig. 2B,C; Movie EV2). We constructed full 3D membrane objects by assigning slicewise segments across adjacent slices based on maximal pairwise Intersection over Union (IoU), thereby stitching 2D contours into consistent 3D volumes (Fig. 2B). This approach reduced spurious objects and improved object consistency along the z-axis. Compared to ground 3D truth annotations, we achieved high segmentation accuracy for nuclei (Appendix Table S2). For membrane segmentation, Cellpose produced accurate 2D segmentations with good per-slice performance with a mean per-slice binary Jaccard index of 0.55 ± 0.24 (binary F1 score = 0.68 ± 0.22, Sparse Jaccard index 0.62 ± 0.16, OaGTC Jaccard index 0.72 ± 0.11). However, stitching these slices into coherent 3D cell objects proved more challenging due to variability in cell morphology and limited Z resolution. To evaluate 3D reconstruction quality, we computed intersection-over-union (IoU) using the same predicted and ground-truth cell volumes as for the 2D objects. The mean 3D binary Jaccard was 0.55 ± 0.17 (binary F1 score = 0.70 ± 0.15, Sparse Jaccard index 0.48 ± 0.16, OaGTC Jaccard index 0.55 ± 0.10), with lower performance in regions with dense packing or ambiguous membrane signal (Appendix Table S2). Qualitative comparison of ground‑truth and predicted labels (Appendix Fig. S2) shows that the automated segmentations recapitulate the global position, size, and overall outline of cells at apical and basal surfaces and across z, albeit with rougher boundaries and reduced fidelity at fine-scale membrane features. These properties indicate that the segmentations are appropriate for capturing coarse-grained shape and positional descriptors used in our subsequent analyses, while fine‑scale membrane geometry should be interpreted with caution.

To reconstruct cell trajectories across developmental time, we tracked segmented nuclear objects, enabling identification of individual cells even when membrane segmentation was incomplete or absent. To integrate nuclear tracking with shape information, each membrane object was matched to the nearest nuclear centroid in 3D space, thereby linking trajectory data to membrane segmentation (Movie EV3). For 3D cell tracking, we used TrackMate 7 (Ershov et al, 2022), which supports labeled nuclear objects as input for spot detection and linking. This approach yielded high linking accuracy (0.919); however, accurate detection of branching events—i.e., cell divisions—remained challenging due to dense tissue packing and limited Z resolution (Appendix Table S3). To address this, we integrated Oneat, a custom-built convolutional neural network (CNN)-based division detection module, into the TrackMate pipeline (Fig. 2D; Movie EV4).

Oneat identifies cell division events as spatiotemporal (TXYZ) coordinates. These coordinates were then used to impose trajectory splitting within TrackMate tracks through our custom-developed TrackMate-Oneat plugin (Fig. 2D). The plugin optionally applies the MARI (Mitosis Angular Region of Interest) principle, which filters division events to retain only those in which daughter cells emerge perpendicular to the mother cell’s nuclear major axis (see “Methods”).

Oneat integration significantly improved mitosis detection compared to TrackMate’s native linking algorithm (TrackMate native branching accuracy  = 0.122, TrackMate-Oneat branching correctness = 0.328). By combining Oneat-predicted division locations with trajectory continuity, the system generated more biologically realistic branching structures and reduced the number of false positives typically produced by Oneat alone (Appendix Fig. S3C–J). However, while applying the MARI principle nearly eliminated false positives, it also reduced true positive detections (Appendix Fig. S3E). As such, the user should choose the division detection strategy—Oneat alone or Oneat with MARI filtering—based on the specific goals and tolerance for false positives in their downstream analyses.

Although reconstructing complete cell lineages remains inherently challenging, since even a single linking error can break lineage continuity (Appendix Fig. S3K), the overall tracking performance was sufficient to achieve our primary objective: generating continuous single-cell trajectories that enable quantification of instantaneous phenotypic features and their integration into complete lineage histories (Fig. 2E; Movie EV5). On average across all datasets, trajectories spanned ≥9.5 h in 33% of cells (mean dataset duration = 19.4 h), with an average track length of 7.47 h, providing sufficient coverage for downstream analyses of morphodynamics, while lineage reconstruction remains imperfect.

### Emerging cell types occupy a continuous and overlapping morphodynamic space

Cell state transitions during development are accompanied by continuous, dynamic changes in morphology and behavior. To quantitatively capture these changes, we defined a *morphodynamic state* for each cell by computing a set of time-resolved morphological and behavioral features, building on methodologies used in recent studies (Alizadeh et al, 2020; Phillip et al, 2021; Copperman et al, 2023). This approach is conceptually analogous to defining a transcriptomic state, in which multiple gene-expression values are assembled into a high-dimensional vector representing a cell’s identity at a given moment.

**Figure 3:**
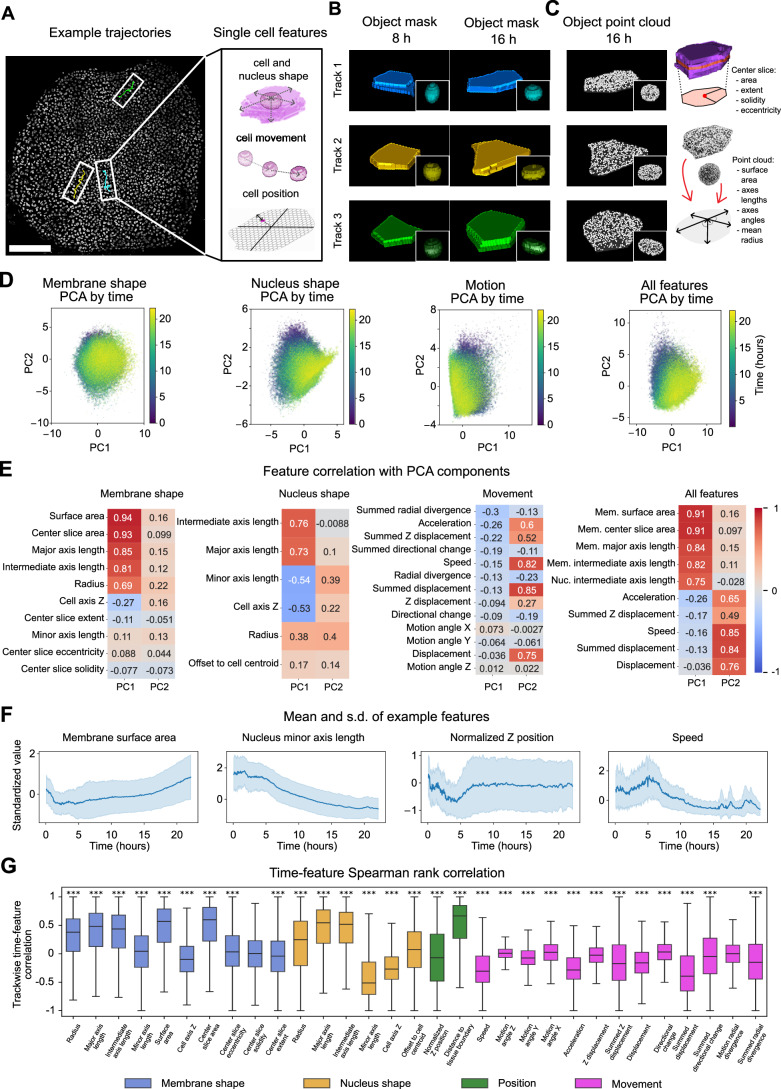
Principal component analysis of single-cell features. (**A**) Each constructed trajectory in time consists of single cells with shape, nucleus shape, movement, and positional features. Scale bar: 300 µm. (**B**, **C**) Membrane and nucleus shapes of example trajectories at 8 and 16 h, with the corresponding point cloud to 16 h. 3D shape measurements of both cell membranes and nuclei are derived from point clouds. For membranes, additional 2D shape descriptor features are calculated from the object center slice. (**D**) 1st and 2nd principal components of membrane shape, nucleus shape, movement, and all features’ feature space. (**E**) Feature correlations with principal components. For all features, only the top five correlating features for PC1 and PC2 are shown. (**F**) Selected feature trends across developmental time for all cells. (**G**) Spearman rank correlations of each feature with time, calculated per trajectory (*n* = 4495 from Dataset 2), and a boxplot shows the distribution of correlations. Box center line shows 50th percentile of the data. Box bounds are set at the 25th percentile (Q1, lower bound); and the 75th percentile (Q3, higher bound). Whiskers extend to Q1–1.5*IQR and Q3 + 1.5*IQR (IQR, interquantile range). Outliers beyond the whiskers are not shown. Asterisks denote features with significant nonzero correlation after FDR correction (***P  <  0.001) (Wilcoxon signed-rank test across trackwise correlations).

For every cell in the dataset, we computed a comprehensive set of features, including spatial position, nuclear and membrane shape descriptors, and instantaneous movement (Fig. 3A; Appendix Table S4). Segmentation masks were converted into 3D mesh representations and resampled into 1024-point clouds per object (Fig. 3C). From these point clouds, we extracted eigenvector-based shape metrics such as surface area, eccentricity, and orientation. In addition, for the membrane objects, widely used 2D descriptors of shape were obtained from each 3D membrane object’s center slice (Fig. 3B,C; “Methods”). These measurements were computed at each time point, yielding a high-dimensional feature vector that characterizes each cell’s morphodynamic identity over time. All feature measurements were calculated at each time point along a cell’s trajectory, forming a high-dimensional morphodynamic feature space.

**Figure EV1:**
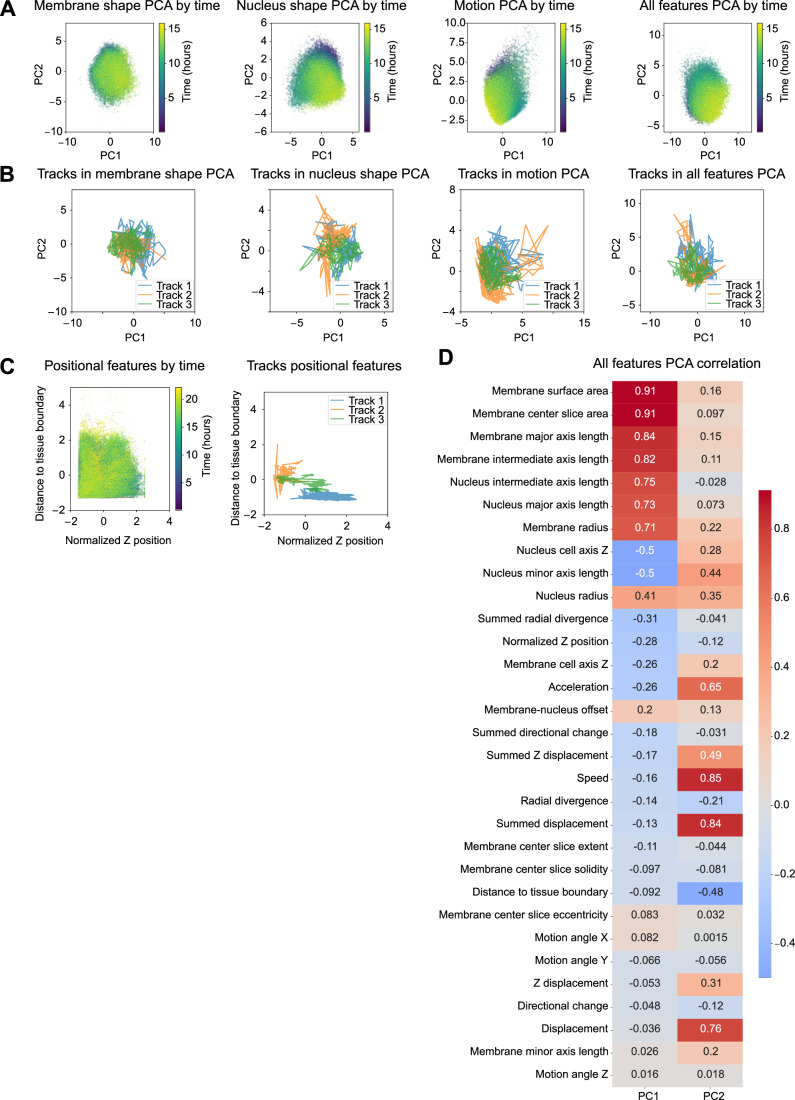
Supplemental data for principal component analysis. (**A**) PCA feature spaces of dataset 1. (**B**) Individual cell trajectories in feature spaces fit for dataset 2. (**C**) Timewise positional features and individual trajectories’ positional features for dataset 2. (**D**) All features’ correlation with all feature PC1 and PC2.

Principal component analysis (PCA) was used to reduce dimensionality across nuclear shape, membrane shape, and motility features, both individually and in combination with positional features (Figs. 3D and EV1C). PCA was performed on a standardized feature matrix comprising *n* = 4269 cell trajectories and *d* = 34 features (Appendix Table S4). In the feature space with all features, the first three principal components accounted for 37.3% of the total variance (PC1: 17.6%, PC2: 10.7%, PC3: 8.9%). However, PCA projections did not reveal distinct clusters corresponding to specific cell types or developmental stages. Instead, cells were distributed continuously in feature space, with slight shifts over developmental time. These results suggest that morphodynamic transitions occur along a spectrum rather than through discrete jumps between feature-space groupings. These observations were consistent across datasets (Figs. 3D and EV1A,C), reinforcing the notion that cell-state transitions in the MCE are gradual and multidimensional, without sharp boundaries in morphodynamic space.

Correlations between individual features and principal components were used to interpret the major axes of variation (Fig. 3E), while time-resolved plots of selected features highlighted global trends in standardized features, such as decreasing motility and progressive cell flattening over time (Fig. 3F). However, most individual features exhibited limited temporal variation and showed no significant trends over time (Appendix Fig. S4). When individual cell trajectories were visualized in feature space, there was no consistent temporal progression, except for positional features, which showed gradual changes over time (Fig. EV1B,C). This was further supported by Spearman rank correlations between feature values and time, which revealed statistically significant, albeit low, correlations for most features (Fig. 3G). Thus, unlike shape-based profiling in cultured cells, where morphologies are highly heterogeneous (Soelistyo et al, 2022), cells within this densely packed 3D epithelium exhibit more constrained morphologies and movement behaviors. These similarities mean that, when all cells are projected into a shared feature space, they are not readily distinguishable based on shape or motion features alone.

### Establishing a ground-truth dataset of cell lineage histories with backtracking

**Figure 4:**
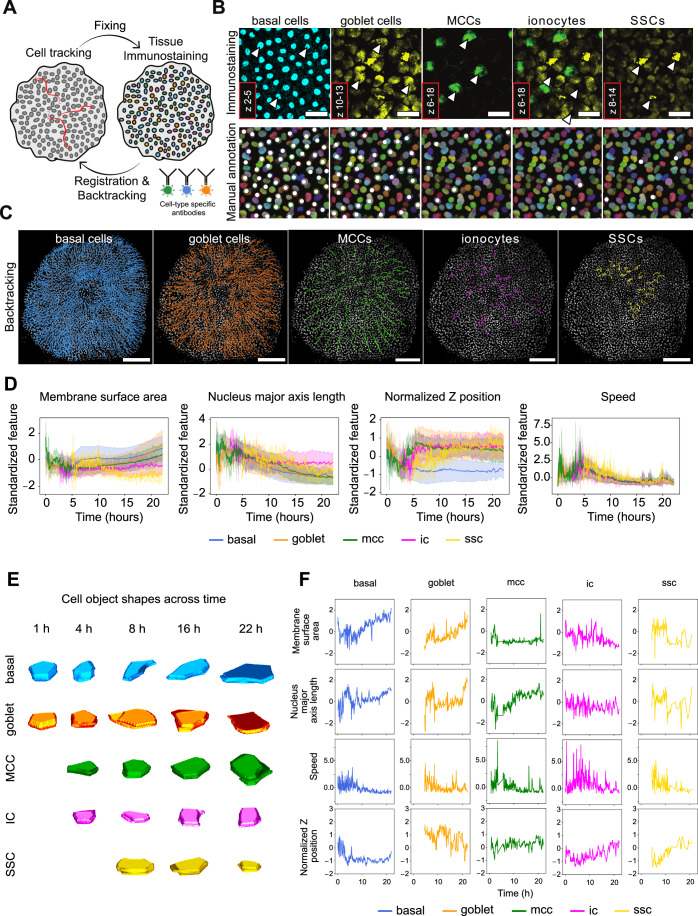
Backtracked cell types’ feature distributions. (**A**, **B**) Live-imaged cells are fixed at the end of the experiment and immunolabeled for MCE cell types (**A**). Cell fate is assigned to a track based on manual annotation of the final timepoint cell type, determined by cell type-specific immunolabelling pattern (**B**, cyan: Tp63, yellow: lectin PNA, green: ɑ-tubulin). Arrowheads show example positions of manually annotated cells. Scale bar: 30 µm. (**C**) Tracks with cell fate labeling, overlaid against the last timepoint. Scale bar: 300 µm. (**D**) Mean and s.d. of selected features, colored by cell type. (**E**) Membrane shape of randomly selected cells, one per cell type, at 1 h, 4 h, 8 h, 16 h, and 22 h. (**F**) Single-cell features of randomly selected cells across time, one per cell type.

Given the limited ability of unsupervised approaches to predict cell fate from morphodynamic features alone, we shifted toward a supervised strategy that incorporates lineage history. To enable this, we first established a ground-truth dataset by assigning final cell identities via endpoint immunostaining and morphology-based annotation of individual lineages. Following completion of live imaging, samples were fixed and immunostained for four well-characterized epithelial cell type markers corresponding to MCCs, goblet cells, small SSCs, and basal cells, as previously described. Cells lacking marker expression but exhibiting characteristic radially intercalating cell morphology and position were manually annotated as ICs (Fig. 4A,B). This endpoint immunostaining labeling enabled us to assign final fates to ~75% of all automatically tracked cell trajectories.

To ensure correct representation of cell lineages, we manually curated trajectories for MCCs (*n* = 156), ICs (*n *= 45), and SSCs (*n* = 11), which are less abundant and more morphologically variable cell types in our dataset. In contrast, trajectories for basal (*n* = 1457) and goblet cells (*n* = 909), which are more abundant and morphologically homogeneous, were initially annotated automatically, with manual correction of randomly selected subsets (*n* = 54 and *n* = 10 for basal and goblet cells, respectively) used for subsequent classifier testing (Fig. 4C; Movie EV6). This annotation produced a high-confidence ground-truth dataset comprising 2576 trajectories for subsequent classification tasks (see below).

To evaluate whether individual features were potentially predictive of final fate, we first examined the temporal dynamics of selected morphodynamic features across cell types (Fig. 4D). Consistent with earlier observations from the global feature space, single features alone provided limited discriminatory power between cell types, showing high within-type variability and overlapping distributions (see also Appendix Fig. S5).

**Figure EV2:**
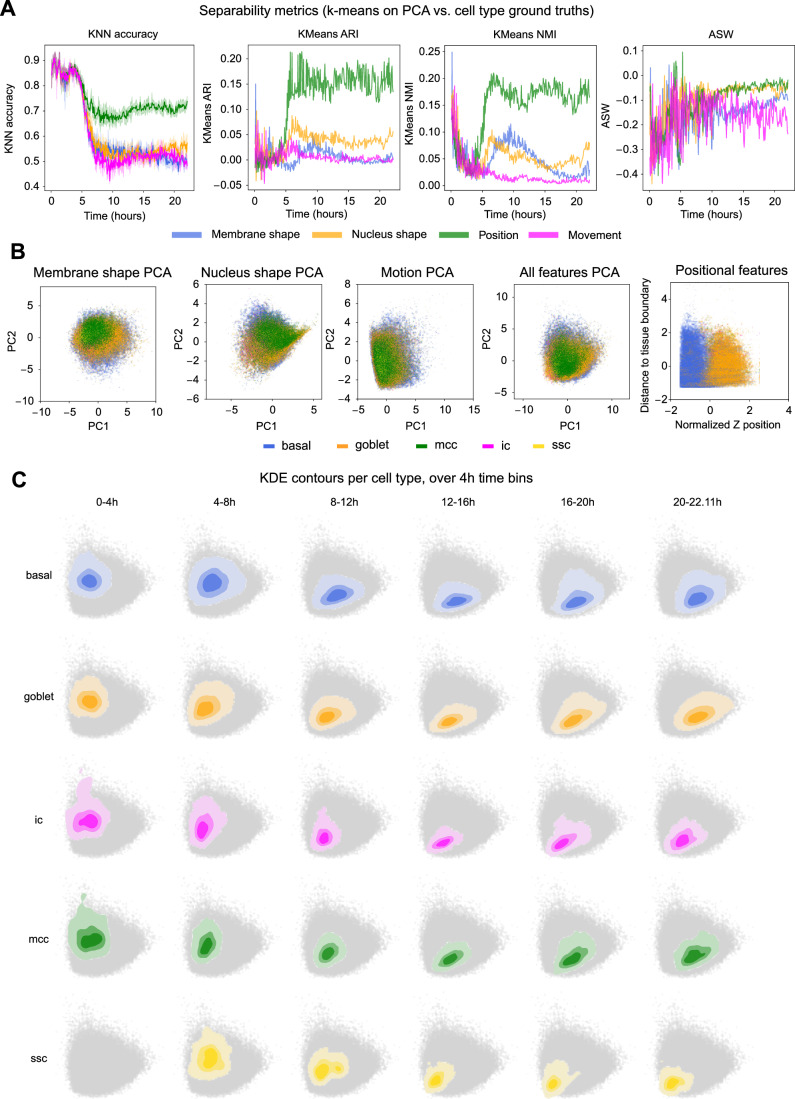
Backtracked cell types in PCA feature space. (**A**) Separability metrics for feature categories. Mean accuracy score (KNN accuracy) based on KNearestClassifier (scikit-learn) of different feature categories, where *k *= 5, Adjusted Rand Index score (KMeans ARI), Normalized Mutual Information score (KMeans NMI) for comparing k-means clustering of selected feature spaces (corresponding to (**B**)) with ground-truth cell type labels. Average silhouette width (ASW) is calculated for different feature categories against ground-truth cell-type labels. (**B**) Cell types labeled in feature spaces corresponding to Fig. 3D and Appendix Fig. S2C. (**C**) Kernel density estimation of time windowed cells in the all features PCA space, estimated per cell type.

To more comprehensively assess whether combinations of features could distinguish cell types, we projected our ground-truth backtracked trajectories into principal component spaces derived from nuclear shape, membrane shape, motility, and positional features, as in Fig. 3D. As before, cells did not form clearly separable clusters by fate in PCA space (Fig. EV2B). This observation held even when trajectories were divided into developmental time windows and analyzed separately by cell type (Fig. EV2C), indicating that feature divergence was subtle and gradual over time.

To quantify the seemingly low separability, we computed unsupervised clustering metrics, including the Adjusted Rand Index (ARI), Normalized Mutual Information (NMI), and Average Silhouette Width (ASW), across multiple feature combinations. Scores were uniformly low (mean ARI = 0.019 ± 0.001, NMI = 0.053 ± 0.001, ASW = -0.156 ± 0.004), with positional features contributing most to weak separability (mean ARI = 0.104 ± 0.004, NMI = 0.133 ± 0.003, ASW = -0.118 ± 0.005), and scores improving over developmental time (Fig. EV2A). Similarly, we trained a K-nearest neighbor classifier (KNN, *k* = 5) using morphodynamic features to predict final cell fate. The mean accuracy of the KNN classifier trained on all features remained at 65.8% across fivefolds (baseline = 54.0% for stratified dummy classifier), with balanced accuracy score at only 28.1% against a baseline of 24.8%, confirming the limited predictive power of unsupervised or shallow models in the absence of lineage context (Fig. EV2A).

Despite this, visualizing selected individual cell trajectories revealed temporally dynamic changes in shape and movement, with subtle but fate-associated trends (Fig. 4E,F; Movie EV7). While shape and movement trends show dynamic changes over time, normalized Z position exhibits the clearest single-cell signature of cell type, with basal cells remaining stably low after early variation, goblet cells showing a descending trend, and ICs, MCCs, and SSCs showing an ascending trend (Fig. 4F). These observations underscore the need for supervised, temporally aware models to efficiently link early morphodynamic behaviors to eventual fate.

**Figure EV3:**
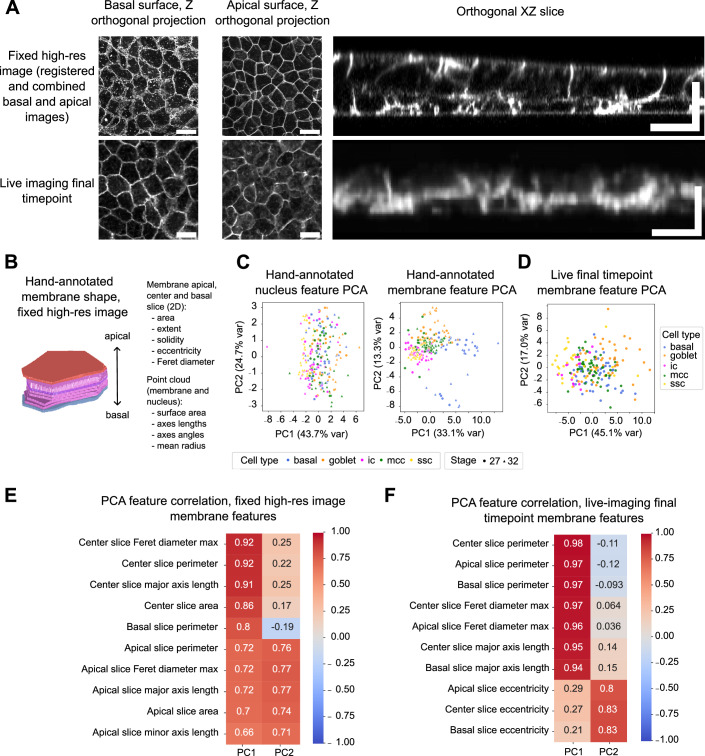
Comparison of high-resolution imaging and live imaging of the membrane signal and feature space of an extended shape feature set. (**A**) Top: Representative example of an animal cap region showing apical and basal Z-projections and an orthogonal XZ slice from the reconstructed high-resolution fixed-tissue dataset, generated by registration and combination of apical and basal image stacks. Bottom: representative example of apical and basal Z projections and orthogonal XZ slice from the final timepoint of a live-imaging experiment (Dataset 2). Scale bar: 30 µm. (**B**) Schematic of the extended membrane feature set used for hand-annotated cells and Live-imaging final timepoint comparison, including 2D membrane descriptors extracted from apical, central, and basal slices and 3D point-cloud–based descriptors for membrane and nucleus shapes. (**C**) Left: PCA of nuclear shape features from the same manually annotated fixed dataset, right: PCA of membrane shape features derived from manually annotated high-resolution fixed samples. (**D**) PCA of membrane shape features derived from automatically segmented cells at the final time point of live imaging (Dataset 2). (**E**, **F**) Correlation matrices of PCA loadings for membrane features from the fixed high-resolution dataset (**E**) and live-imaging final timepoint dataset (**F**).

To rule out whether biologically relevant morphological features, such as the characteristic apical morphology of radially intercalating cell types (as described by e.g., Deblandre et al, 1999; Walentek et al, 2014; Quigley et al, 2011; Stubbs et al, 2006), are obscured by the technical limitations of the live-imaging pipeline, we also performed an extended analysis of cell morphologies using fixed tissue. Using high-resolution fixed-tissue imaging from both the apical and basal sides of the explanted tissue, we reconstructed cell membrane and nuclei volumes with increased structural detail (Fig. EV3A). Manual annotation of cell and nuclei shapes was then performed to ensure cell shapes were captured in their entirety, and 2D shape features were also captured for the apical and basal sides of each membrane object (Fig. EV3B).

Although the separation of cell identity in PCA space based on membrane morphology was modestly improved in this dataset, particularly for basal cells, endpoint shape features alone remained insufficient to resolve fate (Fig. EV3C). Interestingly, the features explaining the largest fraction of variance relate more to the 2D than to the 3D shape descriptors, indicating that they capture meaningful differences between cells that the 3D descriptors are unable to capture (Fig. EV3E). In addition, a similar analysis of nuclear shapes, or cell shapes captured by automatic segmentation at the final timepoint of the live-imaging experiment, failed to reproduce the same separation, further motivating the need for improved live imaging and higher-quality segmentation of membrane shapes (Fig. EV3C,D).

### Morphodynamics-based multiclass classification of cell fate using XGBoost

**Figure 5:**
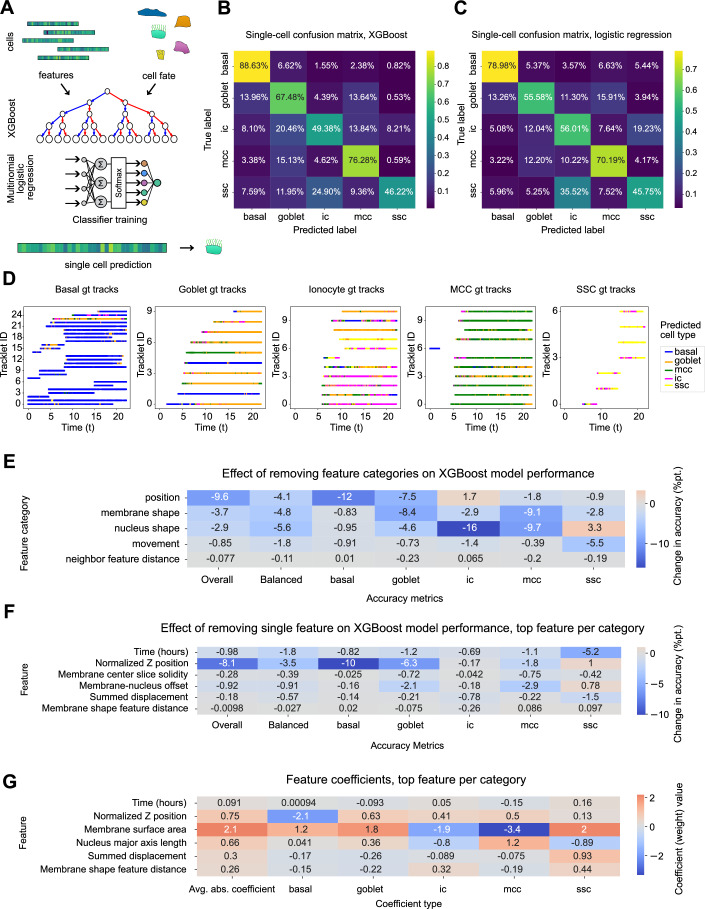
Classifying cell fate using XGBoost and logistic regression. (**A**) Feature vectors of single cells are used as independent features, and cell fate as a dependent feature, for training a classifier model, based on either XGBoost or multinomial (softmax) logistic regressor. Then, each cell in the test dataset is assigned a cell fate based on its feature vector. (**B**, **C**) Confusion matrices for the test dataset, cell-fate prediction vs. ground-truth, for the XGBoost and logistic regression models, respectively. (**D**) Example test dataset trajectories showing single-cell predictions per track. (**E**, **F**) Percent point-wise accuracy difference to baseline accuracy of the XGBoost model for leaving out a feature category (**E**) or a single feature (**F**, only the top feature per feature category is shown). (**G**) Feature coefficients for a logistic regressor model, only the top feature per category is shown. All calculations in (**B**, **C**, **E**, **F**, **G**) are averaged over 20 iterations of a randomized test–train split, model training, and prediction.

**Figure EV4:**
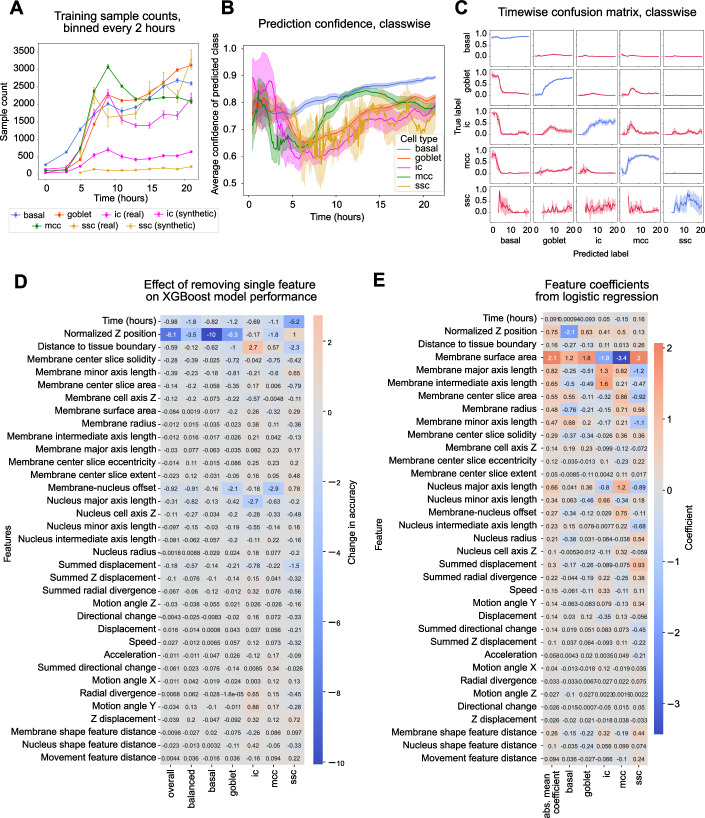
Classifier model performance analysis and feature importances. (**A**) Classifier training dataset sample counts across dataset time, binned every 2 h, mean across *n* = 20 independent iterations of randomized train–test sampling. Error bars represent the standard deviation (±s.d.) across iterations. For basal, goblet, and MCC classes, only real samples were used; for IC and SSC classes, some samples were synthesized using SMOTE oversampling (imbalanced-learn). (**B**) Mean and s.d. prediction confidence of predicted class of XGBoost-based predictions, colored by class. (**C**) Time-wise accuracy confusion matrix of XGBoost-based predictions. (**D**) Percent point-wise accuracy difference to baseline accuracy of the XGBoost model for leaving out a single feature. (**E**) Feature coefficients for a logistic regressor model. All calculations in (**B**–**E**) are averaged over 20 iterations of a randomized test–train split, model training, and prediction.

To assess whether combinations of morphodynamic features could jointly predict cell fate, we trained multivariate multiclass classifiers using XGBoost and a multinomial logistic regression model from scikit-learn (Fig. 5A) (Chen and Guestrin, 2016; Pedregosa et al, 2011). These models employ either ensemble learning (XGBoost) or linear decision boundaries (logistic regression) to predict final cell fates from morphodynamic feature vectors. Each cell at each time point was treated as an independent observation, and models were trained to predict endpoint fates derived from immunostaining-based backtracking. All cells in each track were thus assigned a cell type based on their final cell fate. In addition to morphodynamic features, we included absolute experimental time as an input variable, which improved prediction performance by 0.98% points in total accuracy and 1.8 percentage points in balanced accuracy (Fig. 5F) for the XGBoost model. Model performance was assessed over 20 independent random 80/20 train–test splits, stratified by cell type and time. Across these iterations, both models achieved similar performance: XGBoost reached a mean accuracy of 80.6% (±0.2%), balanced accuracy of 61.4% (±1.2%), and macro F1-score of 0.56, while logistic regression achieved 70.3% (±0.3%) accuracy, 62.9 ( ± 1.2%), and a macro F1-score of 0.45 (Fig. 5B,C). Prediction performance was highest for basal (F1 (XGBoost) = 0.90, F1 (logistic regression) = 0.85), goblet (F1 (XGBoost) = 0.72, F1 (logistic regression) = 0.64), and MCC (F1 (XGBoost) = 0.56, F1 (logistic regression) = 0.45) lineages, which are the major epithelial sublineages in the MCE. Minority classes, including ICs (F1 (XGBoost) = 0.34, F1 (logistic regression) = 0.27) and SSCs (F1 (XGBoost) = 0.25, F1 (logistic regression) = 0.06), remained difficult to classify, despite application of class balancing via a combination of SMOTE-based synthetic oversampling and undersampling (Fig. EV4A).

To evaluate how well classifier predictions reflected the overall differentiation process, we visualized single-cell predictions across trajectories (Fig. 5D). For basal, goblet, and MCC lineages, most predictions along a given trajectory consistently matched the true fate label, indicating that morphodynamic signals are relatively stable and informative at the trajectory level. These data suggest the potential for future models that incorporate full trajectory information for improved classification accuracy.

We next examined how classifier confidence and accuracy evolved over time. As expected, both metrics increased during development for most cell types, consistent with progressive fate specification and increased classwise morphodynamic separation (Fig. EV4B,C). For example, the mean classifier confidence across all cell types rose from 0.80 at ~5 h to 0.89 by ~22 h, accompanied by an increase in single-cell predictions’ class-balanced accuracy from 50.4 to 75.1%.

Interestingly, prediction confidence and accuracy for MCCs peaked at ~15 h (mean confidence = 0.82; accuracy = 80.6%) and declined thereafter (to 0.78 and 57.1%, respectively, by 22 h). These results suggest that MCCs reach a morphodynamic plateau before the final time points, after which increased overlap with goblet cell features may lead to ambiguity or phenotypic convergence (Fig. EV4C).

Early in development, predictions were heavily biased toward the basal cell class, which is both the most abundant and most consistently tracked from the start of imaging. This bias likely arises from both biological and technical factors: basal cells reside in the lower epithelial layer, which is most readily visualized early in development using our inverted microscopy setup, whereas other lineages become detectable only after tissue flattening improves imaging of apical cells.

To understand which features contributed most to prediction, we assessed feature importance via two complementary methods: leave-one-feature-out accuracy drops for XGBoost (Figs. 5E,F and EV4D) and coefficient magnitudes for the logistic regression model (Figs. 5G and EV4E). Across both models, positional features were consistently among the most informative. In particular, normalized Z-position had the greatest predictive value, likely reflecting known apico-basal differences between cell types. For example, goblet cells are restricted to the superficial epithelial layer, whereas basal stem cells remain confined to the basal-most compartment, and these invariant positional biases are well captured by Z-distribution alone. Removing the Z-position caused the largest performance drop in both models.

Interestingly, time was also a strong predictor, especially for SSCs, which emerge later in development. Excluding time as a feature led to a notable decrease in SSC classification accuracy, whereas it had only a minimal effect on predictions for more abundant lineages (Figs. 5F and EV4D). Simultaneously, while the removal of certain features, such as Z-position or membrane-nucleus offset, appeared to marginally improve classification accuracy for the SSC lineage (Fig. 5F), these changes were within a single percentage point and likely reflect high prediction variance in low-sample-size populations rather than robust biological trends.

Among morphological features, nucleus–membrane centroid offset emerged as the most predictive individual variable in the XGBoost model. In contrast, membrane surface area and major axis length were most influential for the logistic regression model (Fig. 5F,G). These differences likely reflect how the two models capture feature interactions: XGBoost models nonlinear interactions, whereas logistic regression relies on additive linear effects. The nucleus-membrane offset is particularly important for MCC prediction, as it may reflect changes in morphology as cells transition from a migratory shape within the plane of the tissue to a vertically oriented configuration during radial intercalation.

Overall, nuclear shape features were comparatively informative to membrane shape features, even though, to the human eye, cell types are more distinguishable by their membrane shape. This result is likely due to greater nuclear segmentation fidelity.

**Figure EV5:**
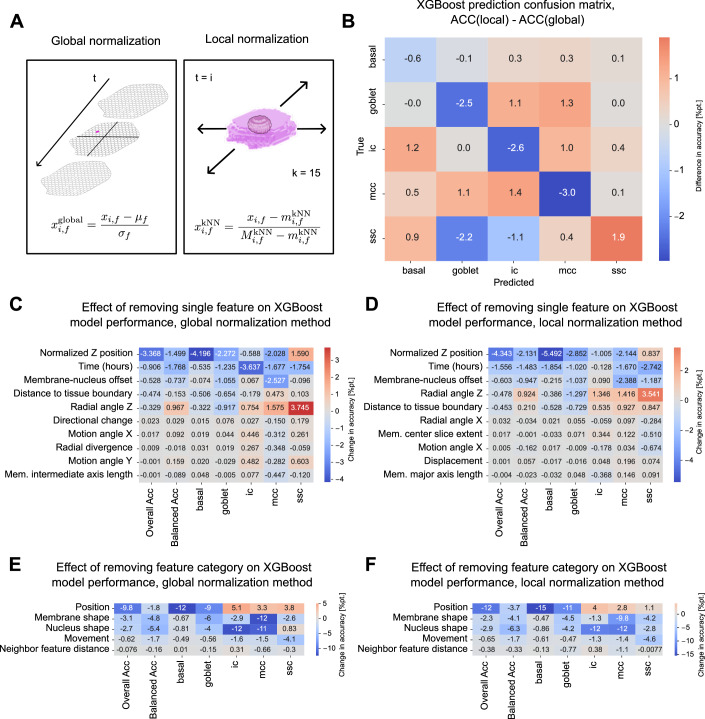
Effect of global versus local feature normalization on XGBoost model performance and feature importance. (**A**) Schematic depicting the compared normalization strategies. Left: In global normalization, cell features are standardized across all cells and all experimental timepoints. Right: In the local normalization strategy, *k* = 15 nearest neighbors in the cell experimental timepoint are used for min–max normalizing feature values. (**B**) Confusion matrix showing the difference in XGBoost prediction accuracy between the local and global normalization strategy. (**C**, **D**) Change in overall accuracy, balanced accuracy, and per cell-type accuracy upon removal of individual features under the global normalization strategy and under the local normalization strategy (**D**). The five highest- and lowest-accuracy features are shown in each heatmap. (**E**, **F**) Change in overall accuracy, balanced accuracy, and per cell-type accuracy upon removal of entire feature categories (position, membrane shape, nucleus shape, movement, neighbor feature distance) under the global normalization strategy (**E**) or the local normalization strategy (**F**).

Conversely, movement features—both instantaneous and time-aggregated—contributed the least to model performance (Fig. 5F). These results align with earlier observations that movement patterns in the developing epithelium are largely collective and not fate-specific. Importantly, although the mosaic patterning of the tissue raises the possibility that locally normalized features might accentuate subtle cell‑type differences, normalizing each feature relative to its spatiotemporal k‑nearest neighbors did not improve model performance nor increase the predictive value of any feature group (Fig. EV5B–F). Instead, the local normalization strategy produced feature‑importance profiles (Fig. EV5D,F) that were highly comparable to those obtained with global z‑score standardization described earlier (Fig. EV5C,E). These results show that the biologically informative variation underlying fate prediction resides at the global tissue scale rather than in local microenvironment‑dependent differences. Together, these analyses demonstrate that multivariate morphodynamic features contain predictive information about cell fate, especially when incorporating positional context and developmental time. However, classification performance remains limited for minority populations, and trajectory-level or temporally contextual models may be needed to fully resolve early fate specification.

## Discussion

In this study, we present a comprehensive experimental and computational pipeline for imaging, segmentation, tracking, and classification of single-cell morphodynamic behaviors during *Xenopus* mucociliary epithelium (MCE) development. We capture nucleus and membrane dynamics of single cells over time and link them to terminal cell fates via immunostaining, thus generating a continuous, high-dimensional phenotypic characterization of *Xenopus* embryonic MCE development. Our work provides a foundation for understanding how cell fate is linked to dynamic changes in shape, position, and movement within a complex, densely packed, and actively developing tissue.

### Cell morphodynamics as a continuous phenotype

Our study supports the idea that cell fate can be partially inferred from morphodynamic features alone. However, unlike in cultured cell systems— where shape-based classification of cell states can effectively resolve distinct drug treatments and cell types (De Vries et al, 2025), our in vivo data show that single cells exist in a highly overlapping but noisy feature space, especially early in development. These results are consistent with observations in other complex tissues, where unsupervised clustering based on cell shape features shows initial promise but continues to face significant challenges in reliably distinguishing cell types; In the anterior visceral endoderm, cell fate is still largely position-dependent despite regional shape (and behavior) trends (preprint: Stower et al, 2023), and in the inner ear, nucleus and cell shape could separate hair cells, but struggled to resolve supporting cell subtypes (Hewitt et al, 2024). Together, these studies underscore that densely packed, morphogenetically active tissues pose challenges for inferring fate from morphology alone, partly due to the limited resolution of 3D tissue imaging and partly due to cell shape similarity in such tissues.

Despite these challenges, our classifier models can extract meaningful signals of phenotypic state, particularly when examining time-resolved predictions across trajectories. Our data mirror developments in single-cell transcriptomics, where pseudotime helps supplement understanding of the developmental timeline when the data are based on stagewise snapshots (Lee et al, 2023). Trajectory-resolved phenotypic data could complement transcriptomics approaches, as it provides a more continuous view of cellular state transitions over time (Weinreb et al, 2018). Unlike scRNA-seq, which captures static snapshots without spatial context, or spatial transcriptomics, which may achieve cellular resolution, but at the expense of transcript depth, morphodynamic tracking provides dense temporal sampling with rich positional information embedded in tissue context. As such, it has the potential to address temporal and spatial gaps in transcriptomics-based fate mapping, offering a new, highly data-rich dimension to the “omics” view of tissue differentiation.

### Comparisons to related approaches

Efforts to track dynamic, developing 3D tissues in a high-throughput manner are ongoing (Amat et al, 2014; Shah et al, 2019; Wen et al, 2021; Bragantini et al, 2025a). Moreover, linking these tracks to single-cell fates to enable large-scale lineage tracing of tissues remains challenging. While lineage tracing embryos and embryonic tissues has been used to map single-cell fates (e.g., Wolff et al, 2018; Coquand et al, 2024), these efforts have not focused on detailed shape/movement data. Our approach emphasizes quantifying cell morphogenesis over time, offering new insights into how the combination of position, shape, and movement may predict fate decisions.

Similarly, while tracking of cell phenotypes via cell shape and movement has been recently studied in vitro (Copperman et al, 2023; Wiggins et al, 2023; Shannon et al, 2024; Dramé et al, 2025), our pipeline demonstrates that even in complex tissues, morphodynamic phenomics can be scaled and analyzed like other ‘omics’ approaches, thus proving the potential of the addition of “phenomics” data, recently demonstrated by Stower et al, 2023 (preprint).

### Limitations and data constraints

Our study was performed using *Xenopus laevis* tissue explants. While this system recapitulates the cell and tissue morphology as well as the cell type composition of the native epithelium, it does not fully reflect the in vivo context, and this limitation should be taken into account when interpreting our results.

Our segmentation and tracking pipeline is optimized for large-scale throughput, which can lead to incorrect lineage reconstruction or assignment, especially during divisions or in less optically accessible regions. In addition, imaging was performed using an inverted microscope, requiring acquisition through the basal cell layer of a dense, multilayered epithelium. Light scattering within the highly pigmented and vesicle-rich tissue further limits consistent resolution of the full apico-basal extent of cells, potentially attenuating subtle three-dimensional morphological features (Fig. EV3A). This likely biases our dataset toward better-segmented and longer-lived trajectories, such as basal cells. To mitigate this, we invested substantial effort in refining segmentation algorithms, generating manually annotated ground-truth data, and performing targeted manual corrections. As a result, our dataset offers a high-quality, representative view of diverse epithelial cell types. These efforts also establish a framework for generating reliable, high-throughput morphodynamic data in future studies with improved scalability and accuracy.

We recognize, however, that accurate 3D segmentation in dense, multilayered epithelia remains a persistent challenge, often leading to partial or ambiguous reconstruction of cell boundaries. Such inaccuracies can obscure biologically meaningful features, including subtle apical or basal morphologies associated with specific cell types.

Moreover, differences in imaging modalities, tissue preparation, and labeling efficiency may introduce biases in feature extraction and subsequent computational analyses. As a result, caution is warranted when interpreting shape-based classifiers or low-variance morphological descriptors, which may reflect limitations in data resolution rather than intrinsic biological similarity. Future advances in high-speed volumetric imaging, improved segmentation algorithms, and multimodal integration of live and fixed-tissue data will be essential to overcome these constraints and enable more faithful characterization of epithelial morphogenesis in vivo.

Our data underscore a central challenge in modeling morphodynamic phenotypes: cell types often fail to form distinct clusters in feature space, particularly at early developmental stages, making unsupervised methods insufficient for fate identification. Consequently, while instantaneous morphology may carry some information about cell state, fate becomes apparent only gradually through changes in shape, position, and movement. As a result, effective classification requires supervised models capable of learning more confined, time-dependent patterns—like CNNs or XGBoost—rather than relying on distinct feature space separation as in omics-based analyses. Our model achieves reasonable fate prediction despite feature overlap, suggesting that morphodynamic features encode consistent temporal signals, even if they are not readily visualized by unsupervised clustering.

### Broader implications and future directions

This work highlights the potential of a morphodynamic single-cell phenomics framework for studying live, developing tissues. By demonstrating that time-resolved cellular features can predict cell fate with meaningful accuracy, it underscores the power of combining high-throughput imaging with advanced computational analysis to investigate developmental mechanisms, tissue self-organization, and cellular responses to perturbation.

Importantly, our quantitative pipeline can be readily extended to other systems or combined with molecular perturbations. For instance, integrating morphodynamic data with single-cell transcriptomics or targeted perturbations of key signaling pathways could reveal causal links between gene expression, developmental signaling, and cell phenotype. Moreover, because the model generates time-resolved predictions for each cell based primarily on shape and position, it could also enhance tracking accuracy by optimizing for continuous cell fate prediction.

While single-cell morphodynamic features in tightly packed epithelial tissues may lack strong separability on their own, future strategies could exploit the inherent spatial patterning of developing tissues. Integrating information about neighboring cells and local context, such as differences in features relative to adjacent cells, patterns of cell–cell interaction, or local geometry, may reveal discriminative signatures that are obscured when analyzing cells in isolation. Such spatially informed models have the potential to substantially improve predictive power and yield deeper insights into the collective dynamics of tissue development.

Ultimately, this approach moves toward a more dynamic view of development, where cell fate is not just defined by signaling pathways but by a continuously changing trajectory through shape, movement, and position in a tissue context.

## Methods

**Table:** Reagents and tools table

Reagent/resource	Reference or source	Identifier or catalog number
**Experimental models**		
*Xenopus laevis*	Nasco, Wisconsin, Fort Atkinson, WI, USA	
**Antibodies**		
Alpha-Tubulin (11H10) Rabbit Monoclonal Antibody	Cell Signaling Technologies	2125
Anti-Acetylated Tubulin antibody, Mouse monoclonal	Sigma-Aldrich	T7451
Anti-p63 antibody [4A4]	Abcam	ab735
Goat anti-Rabbit IgG (H + L) Cross-Adsorbed Secondary Antibody, Alexa Fluor™ 488	ThermoFisher	A-11008
Goat anti-Mouse IgG (H + L) Secondary Antibody, Alexa Fluor 568, Invitrogen	ThermoFisher	A-11004
Donkey anti-Rabbit IgG (H + L) Highly Cross-Adsorbed Secondary Antibody, Alexa Fluor™ 647	ThermoFisher	A-31573
Goat anti-Mouse IgG (H + L) Cross-Adsorbed Secondary Antibody, Alexa Fluor™ 647	ThermoFisher	A-21235
**Oligonucleotides and other sequence-based reagents**		
Plasmids used for mRNA synthesis		
pCS2 + /mem-mNeonGreen	Lance Davidson lab	
pCS2 + /H2B-RFP	John Wallingford lab	
pCS2 + /E-Cadherin-3X-GFP	John Wallingford lab	
pCS2 + /C-Cadherin-GFP	John Wallingford lab	
**Chemicals, enzymes, and other reagents**		
NotI-HF restriction endonuclease	NEB	R3189S
QIAquick Gel Extraction Kit	Qiagen	28506
mMESSAGE mMACHINE™ SP6 Transcription Kit	Invitrogen	AM1340
Nuclease-Free Water (not DEPC-Treated)	Ambion	AM9932
Chorulon®	MSD Animal Health	
Bovine Fibronectin	Sigma-Aldrich	F4759
DAPI	Thermo Scientific	10374168
Lectin PNA From Arachis hypogaea (peanut), Alexa Fluor™ 568 Conjugate	Thermo Scientific	L32458
Lectin PNA From Arachis hypogaea (peanut), Alexa Fluor™ 488 Conjugate	Thermo Scientific	L21409
CasBlock™	ThermoFisher	008120
**Software**		
Zen Black/Zen Blue	Carl Zeiss Microscopy	
Fiji/ImageJ	Schindelin et al, 2012	
Python	Van Rossum & Drake, 2009	
**Other**		
LSM880 Laser scanning confocal microscope	Carl Zeiss Microscopy	
LSM980 Laser scanning confocal microscope	Carl Zeiss Microscopy	
Precision cover glasses thickness No. 1.5H, 25 mm ø	Superior Marienfeld	0117650
Attofluor™ Cell Chamber, for microscopy	Thermo Scientific	A7816

### mRNA synthesis

To transcribe mRNAs for fluorescent protein expression, pCS2 or pCS2+ plasmids were linearized with NotI-HFR (NEB), purified by a gel extraction kit (Qiagen), and then used as templates for in vitro transcription using the mMachine SP6 kit (Ambion). Synthesized mRNA was purified by LiCl precipitation, dried, and dissolved in RNase-free H2O (Ambion).

### *Xenopus laevis* husbandry and embryo manipulation

Wild-type *X. laevis* were obtained from Nasco, Wisconsin, Fort Atkinson, WI, USA. The Danish National Animal Ethics Committee has reviewed and approved all animal procedures, housing, and husbandry conditions under permit number 2017-15-0201-01237. Ovulation was induced in *X. laevis* adult females by injecting 500 U/animal of Human Chorionic Gonadotropin (Chorulon). Eggs were harvested and fertilized in vitro using macerated testes from male frogs in 1/3× Marc’s Modified Ringer’s (MMR) solution. After 2 h, the fertilized eggs were dejellied in a 3% cysteine solution (pH 8.0). Cleaving embryos were then washed and reared in 1/3× MMR solution. For mRNA microinjection, embryos were transferred to a 2% Ficoll in 1/3× MMR solution at the 4-cell stage and injected in both ventral blastomeres twice with 100 pg H2B-RFP and 50 pg 3xGFP-E-Cadherin and 100 pg GFP-C-Cadherin or 100 pg mem-mNeonGreen encoding mRNA constructs, 10 nl per injection. All RNA constructs were diluted in RNase-free H2O (Ambion). H2B-RFP was used in all experiments to label nuclei, and coinjected with either mem-mNeonGreen or a combination of 3x-GFP-E-Cadherin and GFP-C-Cadherin for membrane labeling. After injection, embryos were incubated at 13 °C until NF stages 8–9 for animal cap cutting.

### Animal cap dissection and culturing

Fibronectin (FN) coverslips were prepared by pipetting a 100-µl droplet of 100 µg/ml bovine plasma fibronectin (Sigma-Aldrich) on a 25-mm 1.5 H coverslip (Superior Marienfeld) and incubating o/n RT. Animal caps were cut from dechorionated embryos in 1/3× MMR, washed in Danilchik’s for Amy medium (DFA), and transferred into an imaging chamber fitted with the FN-coated coverslip in DFA. To allow for tissue attachment to the coverslip, animal caps were placed deep in the cell layer toward fibronectin, affixed using a coverslip chip and silicone grease, pressing the tissue down lightly for ~1 h. After attachment, the coverslip chip was removed, and live imaging was set up immediately. During live-imaging experiments, animal caps were cultured while imaging RT, in parallel with intact sibling embryos maintained under identical conditions next to the microscope. Developmental timing in explants was aligned to in vivo stages by morphologically staging sibling embryos according to Nieuwkoop and Faber criteria at the beginning and end of each imaging experiment. For animal caps used for fixed imaging, caps were cultured at 18–20 °C with intact sibling embryos in the same conditions until MEMFA fixation of both caps and embryos at the same timepoint.

### Confocal microscopy and live-imaging setup

Images of explanted animal caps were acquired with confocal laser scanning inverted microscopes Zeiss LSM880 and LSM980 with Airyscan2 detector equipped with a ×40 C-Apochromat W autocorr M27 water immersion objective (NA  =  1.2, working distance = 0.28 mm) (Carl Zeiss Microscopy). All imaging was performed at room temperature, and images were acquired in the regular confocal mode, using excitation lasers of 488 nm for mem-mNeonGreen or 3x-EGFP-E-Cadherin and GFP-C-Cadherin, and 561 nm for H2B-GFP. The obtained tiles were stitched using ZEN Black software (Carl Zeiss Microscopy).

Time-lapse imaging was performed using Experimental Designer mode in ZEN Black software to allow for multiple tile and time interval setups. To accommodate tissue growth within the field of view during the experiment, images were acquired using 3 × 3 tiling at ~0–5 h, 4 × 4 tiling at ~5–10 h, and 5 × 5 tiling until the experiment ended at ~16–22 h. Tiles were acquired with a 10% overlap for all tilings. The time interval was chosen based on the tiling to minimize tracking error and enable reliable cell tracking, using 120, 200, or 300 s intervals, respectively. An optical resolution of 0.69 ×0.69 ×2.0 µm/pixel (XYZ) was used for all live-imaging experiments.

### Post-live-imaging immunostaining

After live imaging, the DFA medium was aspirated, and 2 ml of 3.7% PFA in 1× PBS was added directly to the imaging chamber. Tissue fixation was monitored by acquiring confocal images using the previously described settings. The sample coverslip was transferred to a plastic petri dish, and fixing was continued for a total of 30 min. Immunostaining was further continued as explained in (Willsey, 2021), except for omitting sample H_2_O_2_ bleaching. Primary antibodies used were specific for acetylated-α-tubulin (from rabbit, 1:400, Cell Signalling Technologies) and Tp-63 (from mouse, 1:200, Abcam). Secondary antibodies used were anti-rabbit Alexa-488 or Alexa-647 (from goat or donkey, 1:400, Thermo Scientific) and anti-mouse Alexa-568 or Alexa-647 (from goat, 1:400, Thermo Scientific). Simultaneously with secondary antibodies, samples were incubated in 1:1000 lectin PNA Alexa 568 or Alexa 488 (Thermo Scientific) and 1:1000 DAPI (Thermo Scientific). The sample was placed back in an imaging chamber in 1× PBS and imaged using the same settings as the last timeframe of live imaging, adjusting for 4-channel imaging using a combination of 405, 488, 561, and 639 nm excitation lasers, and setting Z optical sectioning to 1 µm.

### Fixed image acquisition

To obtain high-resolution images of cell morphology in animal caps and embryonic skin, explanted animal caps from embryos injected with mem-mNeonGreen and H2B-RFP mRNAs were incubated at room temperature until sibling reference embryos reached Nieuwkoop and Faber (NF) stages 11, 27, or 32. Both explanted caps and reference embryos were fixed in MEMFA for 2 h and then rinsed several times with 1× PBS over a total of 30 min. Samples were imaged in 1× PBS.

For imaging of the embryonic multiciliated epithelium (MCE), the fixed embryonic epidermis was carefully peeled from the embryo using a combination of forceps and injection needles. Confocal microscopy was performed as described above, except that individual tiled Z-stacks were acquired with an optical resolution of 0.208 × 0.208 × 0.88 µm/pixel (XYZ). Each sample was imaged from both the apical and basal sides, with the coverslip facing the camera.

For NF stage 27 and 32 animal caps, samples were additionally immunostained for cell-type markers as described above and imaged with the apical side facing the coverslip, enabling annotation of MCE cell types.

### Image processing overview

The image-processing pipeline is shown in Fig. 1, and details are discussed in paragraphs below. In short, tiled time-lapse images were exported as 8-bit TIFF files and combined into a time-lapse by matching 3 × 3 and 4 × 4 tiled image canvas sizes to a 5 × 5 tiled image size in ImageJ. Z stacks were tilt corrected using a custom ImageJ macro, and if notable drift was present for the dataset, XYZ drift was corrected using the Fast4DReg ImageJ plugin (Pylvänäinen et al, 2023). Membrane channel images were denoised using a custom-trained CARE model in the CSBDeep Python package (Weigert et al, 2018). Cell nuclei on the nuclei channel were segmented using a custom-trained 3D StarDist model. 3D cell membrane volumes were constructed by segmentation using a custom CellPose 2D mode on the membrane channel, after which 2D slices were stitched into 3D objects using a custom Python script. Segmented nuclei were tracked using TrackMate LAP linker, and trajectories were further corrected by enforcing trajectory branching points using the TrackMate-Oneat plugin on ImageJ. Trajectory data were further processed using the NapaTrackMater Python package, combining cell lineage history with single-cell shape (based on 3D measurements of segmented nucleus and cell membrane objects) and movement and tissue position (based on nuclei objects’ *XYZ* coordinates).

### Image processing of the high-resolution fixed images and manual annotation of cell shapes

To computationally reconstruct high-fidelity, full-thickness images of the animal cap, tiled Z-stacks of NF stage 27 and 32 samples were acquired from both the apical and basal sides. The resulting stacks were aligned using the BigWarp tool within the BigDataViewer plugin for ImageJ. The aligned stacks were then treated as separate channels in ImageJ and maximum intensity projected to generate a single combined image. This procedure was performed for both the membrane and nuclear signal channels. The corresponding immunostained image was subsequently aligned with the reconstructed image to enable cell-type annotation, as described above. Cells annotated by type were then manually segmented for membrane and nuclear morphology using the Napari Labels tool.

### Nucleus segmentation

To segment nuclei in 3D, we began by training a custom model using the StarDist 3D framework (Weigert et al, 2020), optimized for instance segmentation of star-convex shapes in volumetric data. For training, data for annotating 3D nuclear masks were selected from representative ROIs across different developmental timepoints. Augmentations were applied to training patches prior to training. We integrated StarDist segmentation into our VollSeg package, which first finds tissue region of interest from raw nucleus data based on UNET segmentation of Z maximum projected nucleus channels per timepoint, reducing the data input for prediction, and improving the pre-segmentation normalization step on images where tissue area takes up a small portion of the whole image area.

The trained model was applied to all volumetric datasets to segment nuclei in the nuclear channel using the VollSeg framework. To assess segmentation quality, we compared predicted nuclear masks with ground-truth manual annotations. All nuclear segmentations were performed in batch using GPU acceleration via TensorFlow.

### Membrane segmentation

We trained a Cellpose neural network model by utilizing their human-in-the-loop training workflow (Stringer et al, 2021; Pachitariu and Stringer, 2022), using the existing “cyto2” model as a backbone. To generate a training dataset, representative 2D ROIs were selected across different developmental time points. These frames were manually annotated to generate training data. Both labeling and training were performed in the CellPose GUI to generate a custom CellPose model, which was then used on the experimental datasets with a flow threshold of 1.0, a cell probability threshold of −1.0, and a cell diameter of 33.2. We stitched 2D slices into 3D objects using the IoU stitching method from CellPose, using an IoU threshold of 0.6, post-processing each achieved 3D object by using the initial objects’ center slices as object seeds, assigning the rest of the 2D slices to the seed with the highest IoU, maximizing overlap between consecutive slices. Furthermore, residual objects of thickness below 2 slices were discarded. Every image dataset’s membrane channel was batch-segmented using the custom model via the Cellpose command-line interface through PyTorch, after which the above-mentioned postprocessing was applied.

### Segmentation quality measures

We evaluated the segmentation performance of the nucleus and membrane using the F1-score of binarized images, and three variations of the Jaccard Index. The standard Jaccard Index was computed from binarized masks as the ratio of the intersection to the union over all foreground pixels. The Sparse Jaccard Index matched individual ground-truth and predicted objects by computing pairwise intersection-over-union (IoU) scores only for label pairs with overlapping pixels, then averaging the best nonzero IoUs for each ground-truth object. The Object at Ground Truth Centroid (OaGTC) Jaccard Index measured the IoU between each ground-truth object and the predicted object located at its centroid, averaging the results across all ground-truth objects with valid matches. Calculations were averaged across multiple annotated ground-truth images (Appendix Table S2).

### Tracking quality measures

Automated tracking performance (without or with cell division correction) was first benchmarked against automatically generated, manually annotated (“silver” ground-truth) cell tracks on a data ROI (Appendix Fig. S3A) using the Python package “ctc_metrics”, which implements standardized Cell Tracking Challenge (CTC) metrics (Maška et al, 2023; Kaiser et al, 2025). We computed key measures such as detection accuracy (DET), tracking accuracy (TRA), association accuracy (LNK), cell divisions (CT), mitotic branch correctness (BC), and additional metrics like false positives/negatives (TF) and cell cycle annotation accuracy (CCA).

In addition, to evaluate automated tracking performance of our tracking + cell division correction method on complete trajectories, individual manually annotated tracks from a complete dataset (Appendix Fig. S3B) were used as ground-truth and compared against automated tracking results. Each manual track was associated with one or more automated tracks, and transitions between automated track IDs within a single manual track were counted as linking errors. The duration of each manual track and the average duration of its associated automated segments were computed to assess fragmentation. Overall metrics included the average number of linking errors per manual track, average track duration, and the global average duration of automated segments. Additionally, the number of manual and automated cell divisions was quantified, enabling comparison of division detection accuracy.

### Shape feature computation from point clouds and membrane masks

After converting each segmentation label into a binary mask and extracting its surface using scikit-image’s “marching_cubes” algorithm (van der Walt et al, 2014), we obtain a triangulated surface mesh. The vertices of this mesh are treated as a 3D point cloud. From this representation, we extract several shape features, including surface area, eccentricity, and a characteristic radius.

To estimate the surface area of an object, we compute the convex hull of its surface point cloud using scipy.spatial ConvexHull function (Virtanen et al, 2020). The convex hull is the smallest convex polyhedron that encloses all points in the cloud. The total area of the triangular faces of this hull provides an approximation of the object’s surface area. To quantify the anisotropy of the shape, we compute the covariance matrix of the 3D point cloud. This 3 × 3 matrix encodes the spread of points along each axis. Performing eigenvalue decomposition gives the principal directions and the variances along them:

$$\Sigma =\frac{1}{N-1}{\sum }_{i=1}^{N}\left(\vec{{p}_{i}}-\bar{\vec{p}}\right){\left(\vec{{p}_{i}}-\bar{\vec{p}}\right)}^{T}\Rightarrow \sum \vec{{v}_{k}}=\lambda \vec{{v}_{k}}$$

The square roots of the eigenvalues, referred to as eccentricities, measure the object’s spatial extent along each principal axis. Their relative magnitudes indicate how elongated or isotropic the shape is.

To obtain a scalar measure of object scale, we compute the geometric mean of the eigenvalues of the covariance matrix:

$${R}_{{eff}}={\left({\lambda }_{1}{\lambda }_{2}{\lambda }_{3}\right)}^{1/3}$$

This value captures the volumetric spread of the object in all directions and is rotationally invariant. This effective radius provides a single, interpretable descriptor of the object’s overall size, and complements the more detailed eccentricity and surface area metrics.

In addition, to get representative 2D measures from each membrane object, centroids were computed for each unique membrane object in the 3D dataset. The center slice—defined as the z-slice nearest to the rounded z-coordinate of the centroid—was cropped for each object. From these 2D center slices, morphological features including area, Feret diameter, extent, eccentricity, and solidity were quantified using the “regionprops” function from the scikit-image Python package (van der Walt et al, 2014). To assess the difference between automated and high-fidelity manual segmentations, these 2D measurements were also performed on the most apical and most basal slices of each 3D object (two slices for the high-fidelity segmentations, projected together into a single 2D slice).

### Nuclear tracking

To track segmented nuclei over time, we used the Fiji plugin TrackMate 7 (Ershov et al, 2022), allowing us to input integer-labeled segmentation masks directly as objects using the Label Image Detector module. For temporal linking of spots, we applied the Linear Assignment Problem (LAP) tracker, with parameters optimized to accommodate our imaging frame rate and observed nuclear movement, specifically 16 µm linking max distance, 15 µm gap closing max distance, and 3 max frame gap, disabling track splitting and merging, but applying TrackMate-Oneat branching correction (see details below).

The resulting track XML was used as input for our NapaTrackMater Python-based module, which calculates single-cell shape features for nucleus and membrane objects associated with the tracks and additional movement features based on nucleus-tracking lineage information. For each channel, we generated a “master XML” file that combines trajectory XYZT information with single-cell features. This file stores tracks and features, unwrapping them into result CSV files for plotting and further data analysis when needed.

For manual curation of TrackMate-generated tracks, we used Mastodon, an extension of the MaMuT (Wolff et al, 2018) tracking platform based on the BigDataViewer plugin in Fiji (Pietzsch et al, 2015). We input the tracks on the dataset converted to an H5 file and manually corrected cell lineages for as long as the cell was able to be visually followed. For backtracking, we input the manually annotated cell type XYZ locations (see details below) into the final frame of the Mastodon dataset and assigned the manually curated trajectory of the corresponding final-frame cell to its respective cell type. Within this annotation framework, morphological features for downstream analyses were extracted from automatically segmented objects. Membrane and nuclear segmentations overlapping with the XYZT coordinates of manually annotated tracks were assigned to the respective cells. Cell time points without a corresponding segmentation label were excluded from further analysis.

### Division detection with TrackMate-Oneat

We developed a pipeline that leverages deep learning-based action classification to detect cell division events and uses these predictions to improve nucleus tracking. A key component of this system is the Oneat classifier, which was trained to distinguish mitotic from non-mitotic cells based on short temporal sequences of image data (Appendix Fig. S6A). For training, the 3D+t nucleus channel of a dataset was manually annotated for division events. Specifically, mitotic events are annotated on Napari by clicking on the center of the dividing nucleus in a microscopy time series. For each annotated division, a 64 × 64 × 8 voxel crop of three time frames was extracted around the clicked location, centered both spatially and temporally on the mitotic event. This creates a positive training sample. To create negative (non-mitotic) samples, a corresponding number of randomly selected locations are extracted from non-dividing nuclei. These negative and positive samples are used to train the model in a supervised fashion, optimizing a binary classification loss to distinguish mitosis from non-mitosis using a DenseNet-based architecture (Huang et al, 2017) (Appendix Fig. S6B).

For predicting division events from whole datasets, the trained Oneat model processes each object identified from a pre-generated nucleus segmentation. For each segmented nucleus at every time point, a temporal window is constructed by cropping 64 × 64 × 8 voxels for three frames centered on the nucleus XYZ centroid. These volumes are passed through the Oneat model, which classifies each central frame as either mitotic or non-mitotic.

To combine division events with dataset tracking data, the TZYX coordinates of predicted mitotic nuclei were recorded in a CSV file, which was used as input to the TrackMate-Oneat extension of TrackMate. This step uses the predicted division locations to impose a branching point on a trajectory. Suitable daughter cells for mitotic cells are searched within a 16.5 µm radius of the mother cell, and linking is optimized using a Jaqaman linker algorithm (Jaqaman et al, 2008). This biologically informed relinking improves the completeness and accuracy of lineage tracking, especially in datasets with frequent cell divisions.

To avoid spurious links and ensure geometric plausibility, the pipeline also incorporates the Mitosis Angular Region of Interest (MARI) principle. This constraint limits the search for daughter cells to an angular region from the mother cell’s nucleus principal axis of a fit ellipsoid. Candidate daughter spots were defined as those within a radial distance *r* of the mother spot *m* = (*x*_*m*_, *y*_*m*_) whose displacement vector *s* - *m*, with *s* = (*x*_*s*_, *y*_*s*_) the candidate spot position, formed an unsigned angle

$$\theta =\left(\frac{{|a}\cdot \left(s-m\right)|}{\left|\left|a\right|\right|s-{m||}}\right)$$

with the mother’s principal axis *a* not exceeding a threshold *θ*_max_ set by the user.

By restricting candidate daughters to fall within a defined angular region of interest, this method eliminates improbable pairings and enhances the biological realism of the reconstructed lineages. This constraint is especially important in dense tissues, where purely distance-based linking can lead to incorrect associations.

### Cell final fate labeling

To annotate the cell types at the end based on actual cell fates, the final frame of live acquisition and the 3D acquired image of the IF-stained sample were aligned using the BigWarp tool in the BigDataViewer ImageJ plugin. Registration points were identified by comparing the nuclei channels (H2B for the live image and DAPI for the IF image). The warp settings were then applied to all channels of the IF image. Cell type XYZ locations were then manually annotated using the Napari Points tool using the logic in Table 1 (Fig. 4B).

**Table 1:** Identifying features of cell types for manual annotation based on cell immunolabelling.

Staining feature	Cell type label
The cell has prominent acetylated ɑ-tubulin staining	MCC
The cell’s nucleus has prominent Tp63 staining	basal
The cell has prominent and uniform apical lectin staining	goblet
The cell is small and has granular lectin staining	SSC
The cell clearly has none of the above	IC

### Principal components analysis (PCA)

To reduce dimensionality and visualize feature trends, we performed Principal Components Analysis (PCA) using the “sklearn.decomposition.PCA” implementation from the scikit-learn Python package. For each feature category (membrane shape, nucleus shape, position, movement, and all features), standardized feature values were extracted. PCA was fit and transformed on the selected features, and the resulting principal components (PC1 and PC2) were used for downstream analyses and visualization.

The feature contribution to the principal components of each PCA fitting was assessed by calculating the Spearman rank correlation between each feature and the two principal components (PC1 and PC2) from the corresponding feature category PCA. The correlation matrix was computed using “pandas.DataFrame.corr(method = “spearman”)” (McKinney, 2010), and features were ranked by the absolute value of their correlation with PC1. The top-ranked features per category were selected as representatives for further visualization and interpretation.

For principal component analysis of live-imaging datasets, all cells containing the feature categories of interest were used for fitting the feature space. Sample sizes were *n* = 521,695 cells for Dataset 1 (Fig. EV1A) and *n* = 486,120 cells for Dataset 2 (Figs. 3D–F and EV1B–D). For analysis of high-resolution fixed sample, a total of 50 cells (membrane and nuclei shapes) per MCE cell type (except for class SSC, *n* = 33) were manually annotated for volumetric reconstruction of animal caps corresponding to NF stage 27 and 32, yielding paired membrane and nuclear shapes. Shape descriptors were calculated as described above, and PCA was applied separately to the membrane- and nucleus-derived feature matrices. For a comparison with the live-imaging pipeline, up to 50 cells per cell type (basal cells *n* = 50, goblet cells *n* = 50, IC *n* = 18, MCC *n* = 50, SSC *n* = 29) were randomly picked from the final timepoint of Dataset 2, their membrane segmentations extracted, and the same extended shape feature set calculated for the selected objects for a membrane shape feature space fitting.

### Feature-time correlation analysis

For each cell trajectory, standardized features were correlated with experimental time. Tracks were included if their time span was at least 1 h to ensure sufficient temporal variation. For each track, Spearman’s rank correlation coefficient (ρ) was calculated between each standardized feature and the corresponding time in hours, using “scipy.stats.spearmanr” from the SciPy Python package.

The resulting per-track correlation coefficients for each feature were aggregated across tracks to obtain a distribution of ρ values per feature. To assess whether features were, on average, significantly correlated with time, a Wilcoxon signed-rank test was performed on raw ρ values. *P* values were adjusted for multiple testing using the Benjamini–Hochberg false discovery rate (FDR) procedure. Correlation distributions were visualized as boxplots, and statistical significance was indicated by asterisks placed above the corresponding feature’s box, denoting features with FDR-corrected *P* < 0.001.

### Feature separability scores

To quantify cell-type separability in PCA embeddings, several metrics were computed at each time point (Table 2). These metrics were calculated for each feature category and visualized over time to assess how well cell types could be separated in the reduced PCA space.

**Table 2:** Separability metrics for feature category comparison.

Metric name	Definition
KNN accuracy	The classification total accuracy of a k-nearest neighbors classifier (“sklearn.neighbors.KNeighborsClassifier”) trained to predict cell type from PC1 and PC2.
K-Means ARI	The agreement between cell type labels and k-means clustering assignments (“sklearn.cluster.KMeans”), measured using “sklearn.metrics.adjusted_rand_score”.
K-Means NMI	The mutual information between cell type labels and k-means clusters, using “sklearn.metrics.normalized_mutual_info_score”.
ASW	The silhouette score for cell type clusters in PCA space, using “sklearn.metrics.silhouette_score”.

### Data pre-processing for classification

Prior to classification, cell trajectory data were filtered to retain only time points with complete feature and cell-fate information. A curated set of non-redundant features (Pearson cross-correlation <0.9) was selected from the measured features. Features were standardized using z-score normalization. Rows with missing values were dropped. For model training and testing, a single dataset was used for the 5-class XGBoost or logistic regression classifier (see details below). In both cases, tracks were split such that no cell (and its corresponding track across time) appeared in both training and testing sets.

For the XGBoost model, class imbalance was addressed using a combination of undersampling (basal and goblet classes) and SMOTE-based oversampling of ionocyte and SSC classes. Undersampling was stratified over time by dividing the continuous time variable (“t_hours”) into 10 quantile-based bins (each approximately 2 h), preserving the temporal distribution of samples as much as possible. Within each bin, class samples were drawn proportionally so that each class contributed an equal number of examples overall.

Subsequently, the minority classes were oversampled using the “SMOTENC” class from the imbalanced-learn Python package (Chawla et al, 2002; Lemaître et al, 2017). SMOTENC (Synthetic Minority Over-sampling Technique for Nominal and Continuous features) extends the SMOTE algorithm to handle datasets with both categorical and continuous variables. It generates synthetic samples for minority classes by linearly interpolating between a true sample and its nearest true neighbors in feature space for continuous variables, using the binned time as a categorical variable. Oversampling was performed until the number of synthetic samples in each minority class matched the undersampled target size, yielding a fully balanced dataset across all five cell types.

### Single-cell fate prediction

To predict cell fate from individual time points, we trained a multiclass XGBoost classifier using single-cell features extracted from each frame of each tracked cell. Each frame was treated as an independent sample and labeled by the final immunostaining cell type of its parent track. Preprocessed feature vectors were used as the independent variables for training, with experimental time (“t_hours”) included as an additional feature for prediction.

For model training, the dataset was split into training and test sets in a stratified manner across both time points and cell types. Class imbalance was addressed as described above. Model performance was evaluated using accuracy (*ACC*) and balanced accuracy (*BACC*), where

$${ACC}=\frac{{TP}+{TN}}{{TP}+{TN}+{FP}+{FN}}$$

$${BACC}=\frac{1}{K}{\sum }_{k=1}^{K}\frac{T{P}_{k}}{T{P}_{k}+F{N}_{k}}{{{\rm{for}}}} \, {k} \, {{{\rm{classes}}}},$$

and a confusion matrix showing per-class true/false-positive percentages. All comparative calculations were performed by comparing predicted and ground-truth single observations' cell type labels, using “accuracy_score”, “balanced_accuracy_score”, and “confusion_matrix” classes from the scikit-learn Python package, and averaged across 20 independent iterations with random resampling to reduce variability. Additionally, prediction confidence was estimated using the class probabilities output by the XGBoost model via the “predict_proba” function. For each cell, we recorded the predicted class probability (i.e., the model’s estimated probability of its top prediction). These confidence scores were averaged within each cell type and time point for each iteration, then averaged across all iterations. Finally, a 1-h rolling average was applied to visualize temporal trends in prediction confidence.

To assess the contribution of individual features to the XGBoost model, we performed an analysis by iteratively retraining the model with each feature / feature category removed and measuring changes in accuracy metrics. This was also applied iteratively across 20 independent iterations, taking the mean change in balanced accuracy as the metric to sort the features.

For comparison, we also trained a logistic regression classifier using the same input data, using the “LogisticRegression” class from the scikit-learn Python package. This model was implemented with L1 regularization and trained using the “saga” solver with a maximum of 1000 iterations. The logistic regression results were used for comparison with a classwise confusion matrix; additionally, feature weights were output from the trained model to assess feature importance. Model performance assessment was performed across 20 iterations and averaged across iterations, similarly to XGBoost performance assessment.

### Comparison of normalization strategy on XGBoost performance

To assess how feature normalization affects classification performance and feature importance, we compared two preprocessing strategies applied to the same single‑cell dataset prior to XGBoost model training. In addition to the global normalization approach described above, we tried a local normalization approach. Each cell’s feature values were normalized relative to the distribution of its *k* nearest spatial neighbors at the same timepoint:

$${x}_{i,f}^{{kNN}}=\frac{{x}_{i,f}-{m}_{i,f}^{{kNN}}}{{M}_{i,f}^{{kNN}}-{m}_{i,f}^{{kNN}}}$$

Here, *x*_*if*_ denotes the raw value of feature *f* for cell *i*. The term ${kNN}$ refers to the set of the *k* = 15 nearest spatial neighbors of cell *i* among all cells recorded at the same experimental timepoint, determined using Euclidean distance in 3D coordinate space. The value of feature *f* was then rescaled using a local min–max transform based on kNN neighbors (${m}_{i,f}^{{kNN}}$ denotes the local minimum, ${M}_{i,f}^{{kNN}}$ denotes the local maximum), such that each locally normalized feature reflected the position of the cell within its immediate spatiotemporal context. For both normalization strategies, the resulting feature matrices were processed through the identical training pipeline described above, including track‑wise train/test splits, temporal stratification, undersampling, and SMOTE‑based oversampling, and training of a 5‑class XGBoost classifier. Model performance was compared between the two normalization schemes using accuracy, balanced accuracy, and confusion matrices averaged over 20 independent iterations. To quantify the contributions of individual features and feature categories, we performed leave-one-feature-out (LOFO) and leave-one-category-out (LOCO) analyses under both normalization strategies.

### Blinding

No blinding was done.

## Supplementary information

Peer Review File

Appendix

Movie EV1

Movie EV2

Movie EV3

Movie EV4

Movie EV5

Movie EV6

Movie EV7

Expanded View Figures

## Data Availability

The datasets and computer code produced in this study are available in the following databases: The code for the image data processing workflow: Github (https://github.com/kapoorlab/CopenhagenWorkflow). Code for data postprocessing, plotting, and analysis: Github (https://github.com/Sedzinski-Lab/trackomics/tree/v1_paper/). Models used for processing the data: Huggingface (https://huggingface.co/datasets/KapoorLabs-Copenhagen/Xenopus_Models/tree/main). Source microscopy data: BioImage Data Archive S-BIAD2969. Source data for producing figures: BioStudies database S-BSST2777. The source data of this paper are collected in the following database record: biostudies:S-SCDT-10_1038-S44320-026-00212-x.
