# Supplementary material for: Single-cell morphodynamics predict cell fate decisions during mucociliary epithelial differentiation
Source: Mol Syst Biol. 2026 May 11;22(7):1040–69. doi: 10.1038/s44320-026-00212-x (PMC13328729; doi:10.1038/s44320-026-00212-x)
Supplement: Supplementary file 2 — Appendix [file 44320_2026_212_MOESM2_ESM.pdf]

Appendix for

Single-cell morphodynamics predict cell fate decisions during mucociliary epithelial differentiation.

Table of contents

Appendix Figure S1 1
Appendix Figure S2 3
Appendix Figure S3 4
Appendix Figure S4 6
Appendix Figure S5 7
Appendix Figure S6 8
Appendix Table S1 9
Appendix Table S2 10
Appendix Table S3 11
Appendix Table S4 12

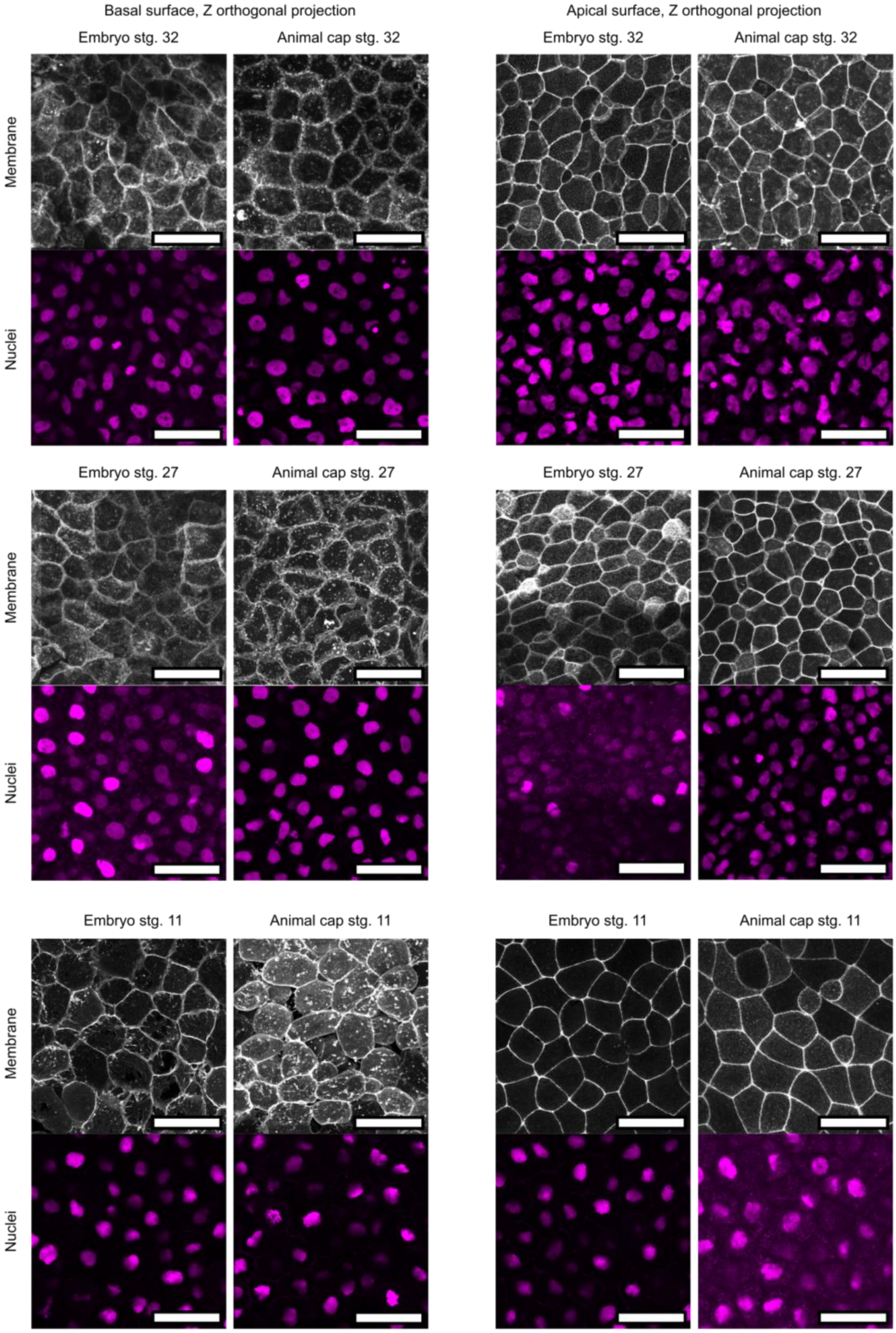

Appendix Figure S1. Comparison of cell and nuclear shapes in embryos and animal caps. Embryonic MCE from embryos in NF stages 32, 27, and 11, and corresponding animal caps, imaged from the apical and basal surfaces and orthogonally projected in Z. Membrane labelling: mem-mNeonGreen, nuclei labelling: H2B-RFP. Scale bar: 50 µm.

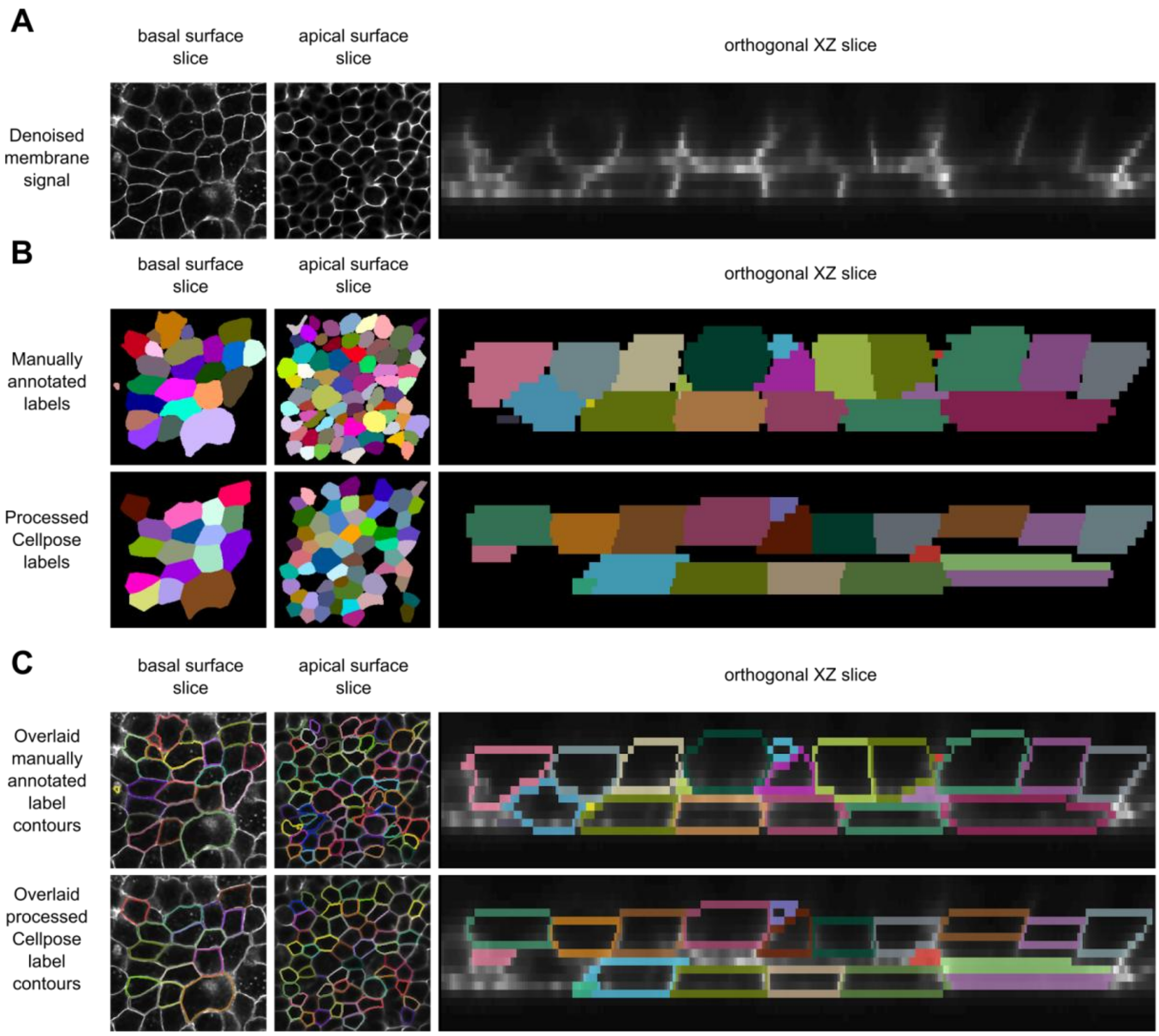

Appendix Figure S2. Comparison of Cellpose segmentation to membrane signal and ground truth annotation labels. Representative slices of the image stacks used for membrane segmentation quality measures in Table S2. **A.** Membrane signal used for manual annotations and Cellpose segmentation. **B.** Colorized labelling of ground truth, manually annotated labels, and processed Cellpose labels. **C.** Labels overlaid on membrane signal.

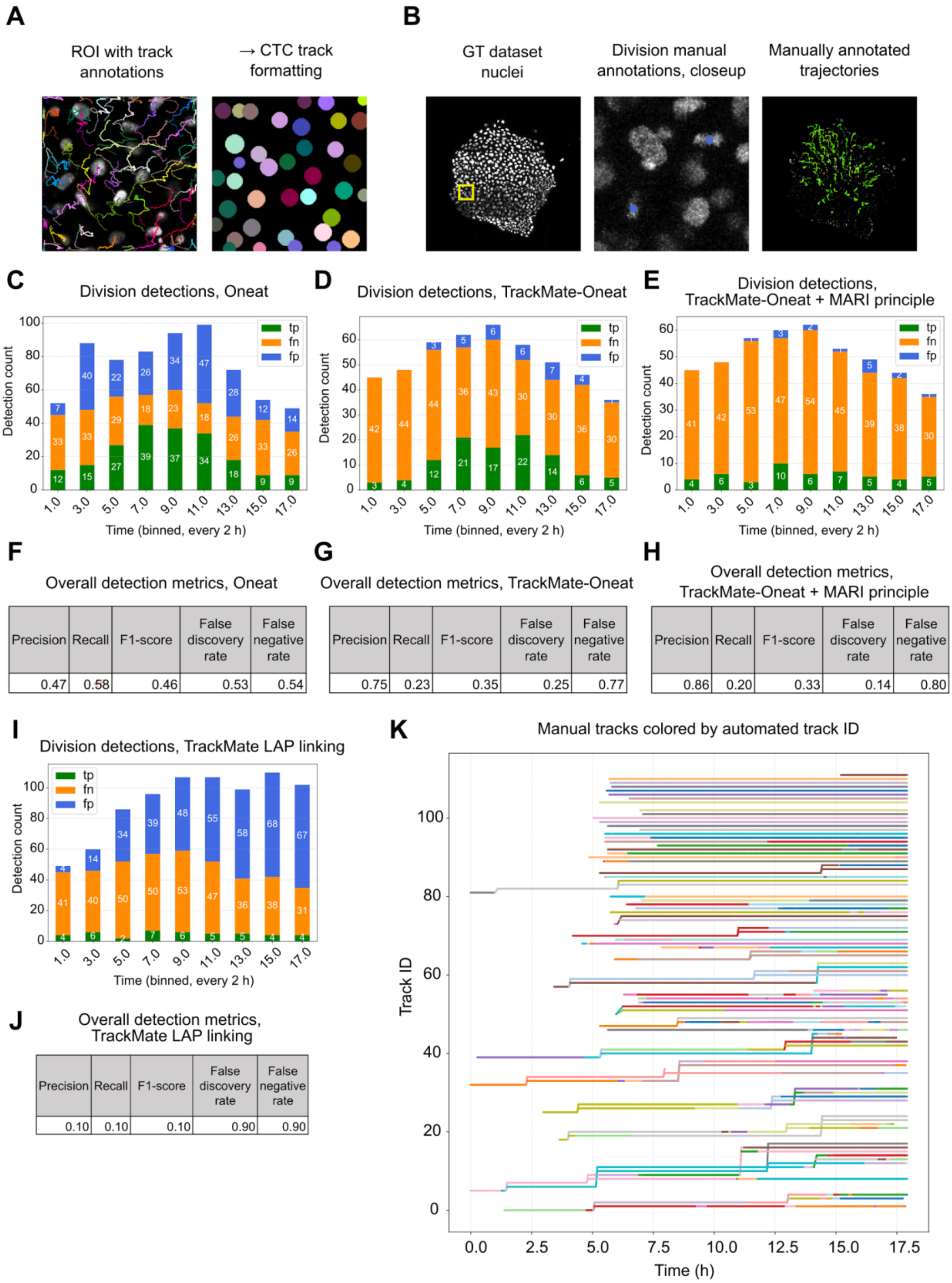

F Overall detection metrics, Oneat

Precision	Recall	F1-score	False discovery rate	False negative rate
0.47	0.58	0.46	0.53	0.54

G Overall detection metrics, TrackMate-Oneat

Precision	Recall	F1-score	False discovery rate	False negative rate
0.75	0.23	0.35	0.25	0.77

H Overall detection metrics, TrackMate-Oneat + MARI principle

Precision	Recall	F1-score	False discovery rate	False negative rate
0.86	0.20	0.33	0.14	0.80

J Overall detection metrics, TrackMate LAP linking

Precision	Recall	F1-score	False discovery rate	False negative rate
0.10	0.10	0.10	0.90	0.90

Appendix Figure S3. Quality metrics for tracking and division detection. A-B. Cell Tracking Challenge format quality estimations in Table S3 are based on an ROI of a dataset (A), where each cell is manually tracked for as long as it appears in the ROI. Manual track annotations are formatted in CTC format, to which automated tracking with TrackMate is then compared to estimate tracking quality. Division metrics in C-J are based on a dataset where each cell division is manually annotated (B). In this dataset, selected tracks are also annotated and compared to automated tracking in Table S3 and K. **C-E, I.** Division detections for Oneat (not connected with tracks), TrackMate-Oneat (Oneat divisions connected with TrackMate tracks), TrackMate-Oneat + MARI principle (TrackMate-Oneat with max boundary set for angle between mother cell and daughter cells), and TrackMate "native" track splitting, enabled in TrackMate LAP linking algorithm. **F-H, J.** Corresponding detection metrics. **K.** Manually annotated ground truth tracks colorized by the Track ID assigned by automatic tracking used for the experiments. C, D, E, I: tp = true positive, fn = false negative, fp = false positive.

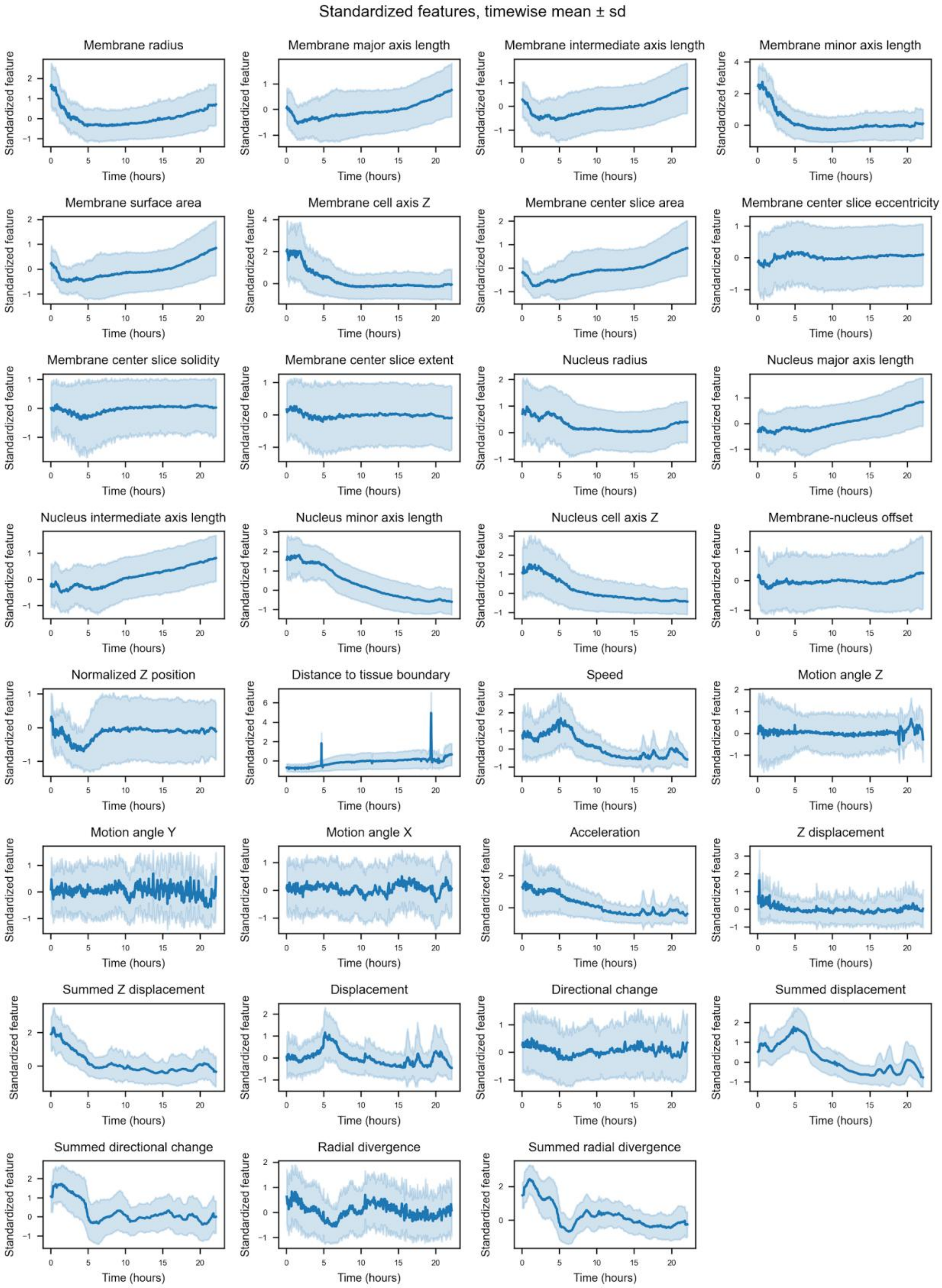

Appendix Figure S4. Mean and s.d. of all features across time, dataset 2.

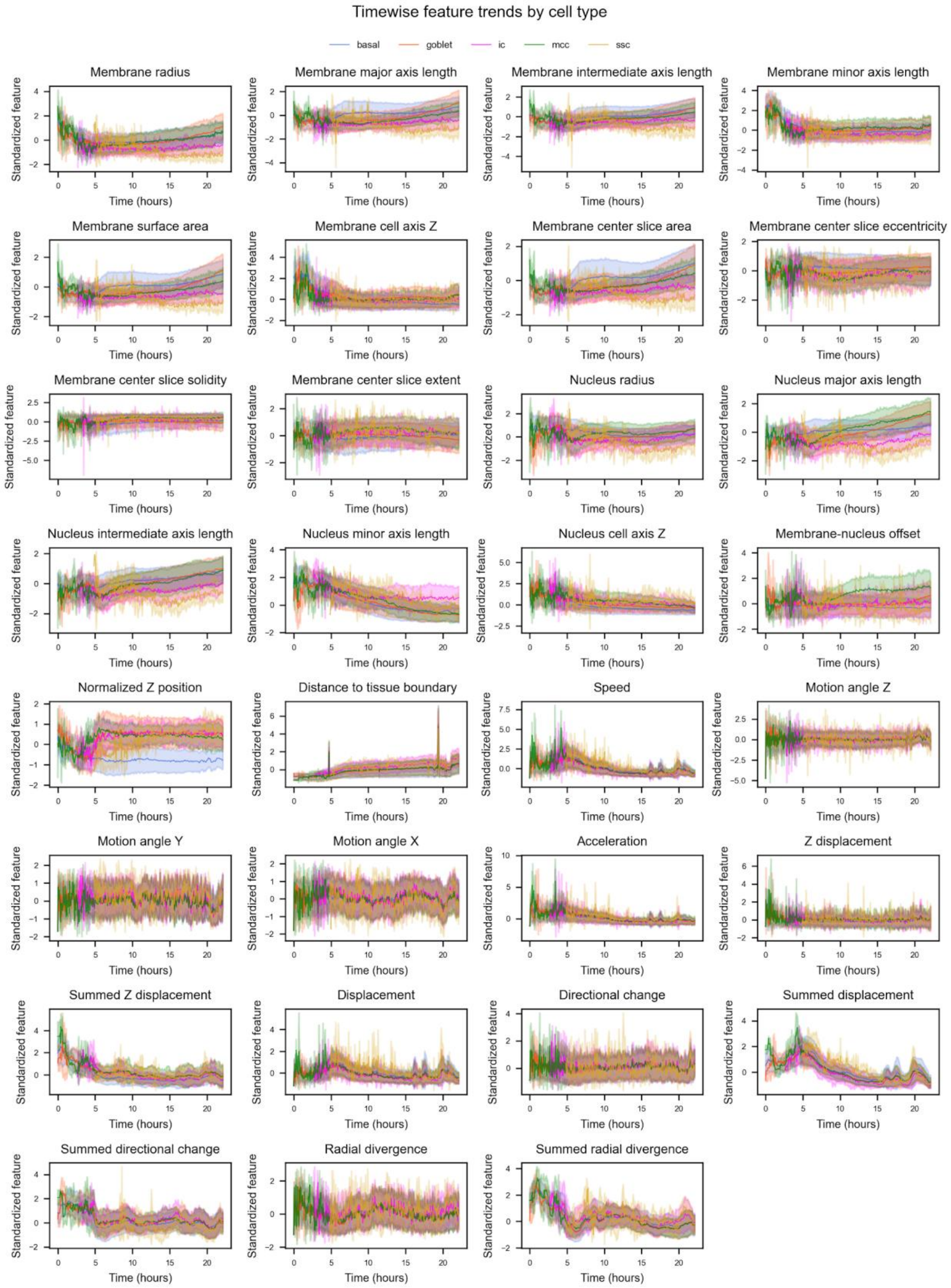

Appendix Figure S5. Mean and s.d. of all features of cell type annotated cells across time, colored by cell type, dataset 2.

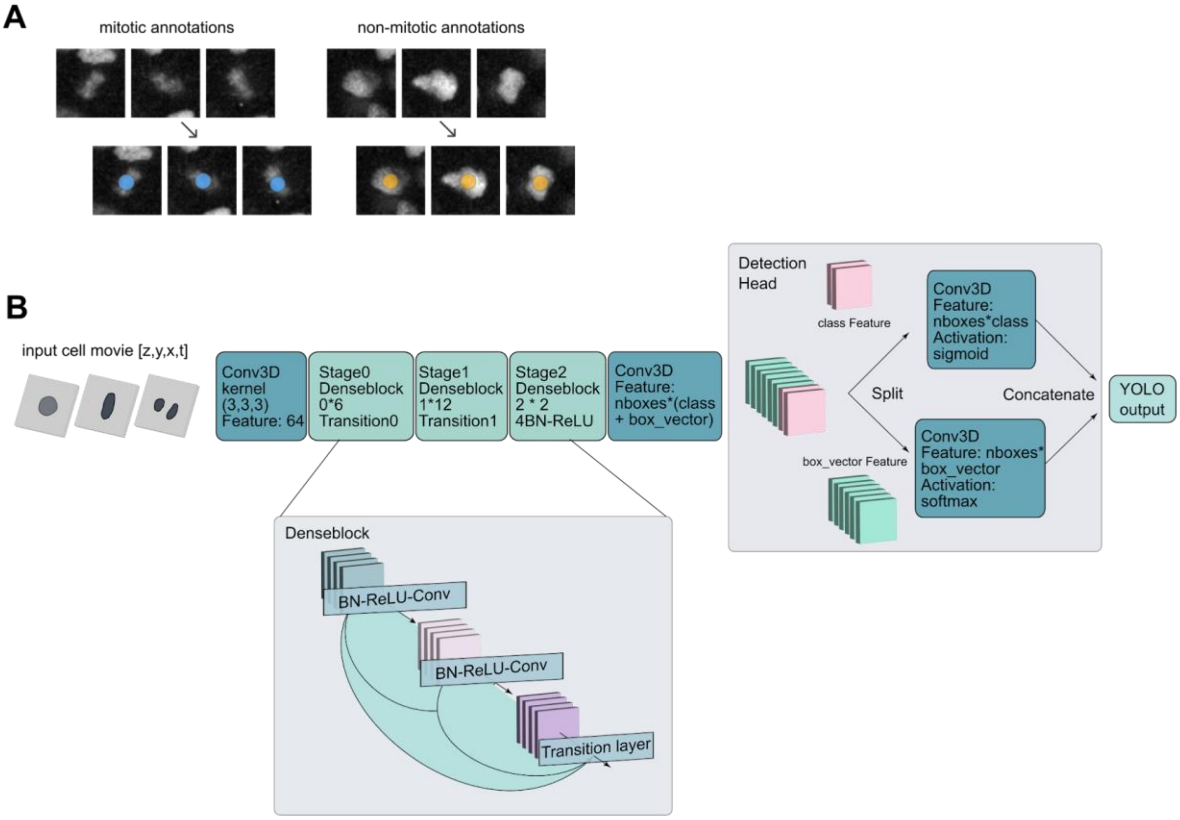

Appendix Figure S6. Oneat model structure. A. Training data annotations in Napari for training a mitosis classifier. **B.** CNN architecture. The input data consists of 3 timepoints, an 8 x 64 x 64-pixel crop centered on the annotation ZYX+t centroid. The output is the probabilities for classification as mitotic or non-mitotic.

Appendix Table S1: Experiment overview

Experiment n	n cells	n tracks	Experiment duration (h)	Mean track duration (h)	Channels	Used for experiments
1	663262	4254	15.95	9.56	Membrane, nuclei	Fig. 2, EV1
2	746561	7583	22.11	6.69	Membrane, nuclei	Fig. 1, 2, 3, 4, 5, EV1, EV2, EV3, EV4, EV5, App. S4, App. S5
3	822760	8907	20.18	6.15	Membrane, nuclei	Fig. 2

Appendix Table S2: Segmentation quality control measures

StarDist nucleus segmentation trained on own 3D labeled data

n gt images	Binary F1-score	Binary Jaccard index	Sparse Jaccard index	n gt objects	n predicted objects
10 x (200 x 200 x 20 voxels)<br>2 x (400 x 400 x 17 voxels)	0.821	0.699	0.643	886	984

Cellpose membrane segmentation trained on own 2D labeled data, 2D slice quality estimation (from 3D labeled objects)

n gt images	Binary F1-score	Binary Jaccard index	Sparse Jaccard index	OaGTC Jaccard index	n gt slices	n predicted slices
5 x (250 x 250 x 19 voxels)	0.679	0.552	0.618	0.724	2173	1788

Cellpose membrane segmentation trained on own 2D labeled data, native 3D stitch method (IoU threshold 0.6)

n gt images	Binary F1-score	Binary Jaccard index	Sparse Jaccard index	OaGTC Jaccard index	n gt objects	n predicted objects
5 x (250 x 250 x 19 voxels)	0.698	0.552	0.496	0.519	339	1059

Cellpose membrane segmentation trained on own 2D labeled data, custom 3D stitch method

n gt images	Binary F1-score	Binary Jaccard index	Sparse Jaccard index	OaGTC Jaccard index	n gt objects	n predicted objects
5 x (250 x 250 x 19 voxels)	0.700	0.549	0.485	0.553	339	366

Appendix Table S3: Tracking quality control measures

Cell Tracking Challenge format metrics of comparison against "silver" ground truth, TrackMate LAP tracking with splitting enabled

Detection accuracy	Tracking accuracy	Linking accuracy	Track fractions	Complete tracks	Branching correctness	Cell cycle accuracy
0.943	0.940	0.919	0.888	0.413	0.122	N.A.

Cell Tracking Challenge format metrics of comparison against "silver" ground truth, TrackMate LAP tracking + cell divisions from TrackMate-Oneat

Detection accuracy	Tracking accuracy	Linking accuracy	Track fractions	Complete tracks	Branching correctness	Cell cycle accuracy
0.943	0.940	0.917	0.880	0.408	0.328	N.A.

Comparison to full dataset, fully annotated divisions and selected manually corrected ground truth tracks

n manually edited tracks	Mean gt track duration (h)	Mean duration of correctly tracked segment (h)	Mean n linking errors per track	n gt divisions in manual tracks	n gt divisions in automated tracks
51	12.09	3.12	4.10	36	14

Appendix Table S4: Single cell features

**Movement (dynamic) measures for cell p in timepoint i, $p_i = (x_i, y_i, z_i)$**				
**Measure**	**Feature name**	**Description**	**Unit**	**Equation**
XYZ Displacement	Displacement	Vector displacement of the cell between timepoints $i-1$ and i	μm	$\vec{d_i} = \vec{p}_i - \vec{p}_{i-1} = (x_i - x_{i-1},\ y_i - y_{i-1},\ z_i - z_{i-1})$
Speed	Speed	Instantaneous speed of cell, where $\Delta t = t_i - t_{i-1}$	μm/sec	$v_i = d(p_{i-1}, p_i)/\Delta t$
Acceleration	Acceleration	Instantaneous acceleration of the cell, where $\Delta t = t_i - t_{i-1}$	μm/sec^2	$a_i = v_i - v_{i-1}/\Delta t$
Motion angle X	Motion_Angle_X	Angle between the movement vector and the X-axis	deg	$\theta_X = \cos^{-1}\left(\frac{v_x}{\lvert\mathbf{v}\rvert}\right)$, where $\mathbf{v}_{xyz} = \mathbf{p}_i - \mathbf{p}_{i-1} = (v_x, v_y, v_z)$
Motion angle Y	Motion_Angle_Y	Angle between the movement vector and the y-axis	deg	$\theta_Y = \cos^{-1}\left(\frac{v_y}{\lvert\mathbf{v}\rvert}\right)$, where $\mathbf{v}_{xyz} = \mathbf{p}_i - \mathbf{p}_{i-1} = (v_x, v_y, v_z)$
Motion angle Z	Motion_Angle_Z	Angle between the movement vector and the Z-axis	deg	$\theta_Z = \cos^{-1}\left(\frac{v_z}{\lvert\mathbf{v}\rvert}\right)$, where $\mathbf{v}_{xyz} = \mathbf{p}_i - \mathbf{p}_{i-1} = (v_x, v_y, v_z)$
Absolute Z displacement	Displacement_Z_Abs	Change in cell position along the Z-axis between timepoints $i-1$ and i	μm	$\Delta z_i = \lvert z_i - z_{i-1}\rvert$
Directional change	Directional_Change	Change in movement direction (XY plane) between consecutive timepoints	deg	$\Delta\theta_i = \theta_i - \theta_{i-1}$, where θ is the XY movement angle
Motion radial divergence	angle_difference	Absolute angle between the XY movement direction and the vector from tissue centroid to cell position	deg	$\phi_i = \left\lvert\theta_i^{\mathrm{XY}} - \theta_i^{\mathrm{centroid}}\right\rvert$, normalized to $[-180°, 180°]$, where $\theta^{\mathrm{XY}} i = \arctan 2(y_i - yi - 1, x_i - x_{i-1})$ and $\theta_i^{\mathrm{centroid}} = \arctan 2(y_i - y_c, x_i - x_c)$, where (x_c, y_c) is the tissue centroid at t_i
Summed displacement (0.5 hr)	Displacement_summed	Total displacement within a ± 0.25 hr window centered at current timepoint	μm	$s_i = \sum_{j \in W_i} d_j$, where W_i is the set of points in the 0.5 hr window around t_i
Summed Z displacement (0.5 hr)	Displacement_Z_summed	Total Z-axis displacement within a ± 0.25 hr window centered at current timepoint	μm	$s_i^Z = \sum_{j \in W_i} \Delta z_j$, where W_i is the set of points in the 0.5 hr window around t_i
Summed directional change (0.5 hr)	Directional_Change_summed	Total absolute directional change (in XY plane) within a ± 0.25 hr window	deg	$s_{\theta_i} = \sum_{j \in W_i} \lvert\Delta\theta_j\rvert$, where W_i spans 0.5 hr centered at t_i
Summed radial divergence (0.5 hr)	angle_difference_summed	Total radial divergence within a ± 0.25 hr window	deg	$s_{\phi_i} = \sum_{j \in W_i} \phi_j$, where W_i spans 0.5 hr centered at t_i

Shape 3D measures for membrane and nucleus objects

Measure	Feature name in code	Description	Unit	Notes
Radius	Radius	Geometric mean of the eigenvalues of the covariance matrix	μm	$R_{\text{eff}} = (\lambda_1 \lambda_2 \lambda_3)^{1/3}$ from object covariance eigenvalues
Surface area	Surface_Area	Summed up convex hull triangulation area	μm^2	from SciPy 'ConvexHull' function
Major axis length	Eccentricity_Comp_First	Largest spread of the point cloud along the principal axis	A.U.	$e_1 = \sqrt{\lambda_1}$, where λ_1 is the largest eigenvalue of Σ_P
Intermediate axis length	Eccentricity_Comp_Second	Intermediate spread along the second principal axis	A.U.	$e_2 = \sqrt{\lambda_2}$, where λ_2 is the second largest eigenvalue of Σ_P
Minor axis length	Eccentricity_Comp_Third	Smallest spread along the third principal axis	A.U.	$e_3 = \sqrt{\lambda_3}$, where λ_3 is the smallest eigenvalue of Σ_P
Cell Axis X	Cell_Axis_X	Angle between the first principal axis of the cell and the X-axis	deg	$\theta_X = \cos^{-1}\left(\frac{\mathbf{e}_1 \cdot \mathbf{u}_X}{\lvert\mathbf{e}_1\rvert \lvert\mathbf{u}_X\rvert}\right)$
Cell Axis Y	Cell_Axis_Y	Angle between the first principal axis of the cell and the Y-axis	deg	$\theta_Y = \cos^{-1}\left(\frac{\mathbf{e}_1 \cdot \mathbf{u}_Y}{\lvert\mathbf{e}_1\rvert \lvert\mathbf{u}_Y\rvert}\right)$
Cell Axis Z	Cell_Axis_Z	Angle between the first principal axis of the cell and the Z-axis	deg	$\theta_Z = \cos^{-1}\left(\frac{\mathbf{e}_1 \cdot \mathbf{u}_Z}{\lvert\mathbf{e}_1\rvert \lvert\mathbf{u}_Z\rvert}\right)$
Membrane-nucleus-offset / offset to cell centroid	mem_nuc_offset	Distance between nucleus object centroid and nucleus object centroid	μm	

Shape 2D measures for membrane object center slices (From skimage.measure.regionprops)

Measure	Feature name	Description	Unit	Notes
2D Area	mem_2d_area	Area of the membrane object in 2D projection	μm^2	
2D Eccentricity	mem_2d_eccentricity	Eccentricity of ellipse that has same 2nd-moments as membrane	A.U.	Value between 0 (circle) and 1 (line)
2D Solidity	mem_2d_solidity	Ratio of object area to convex hull area	A.U.	$= \frac{\text{Area}}{\text{Convex Hull Area}}$
2D Extent	mem_2d_extent	Ratio of object area to bounding box area	A.U.	$= \frac{\text{Area}}{\text{Bounding Box Area}}$

Positional features for nucleus objects

Measure	Feature name	Description	Unit	Notes
Normalized Z position	POSITION_Z_norm	Cell's Z position normalized relative to nearby neighbors within a 150 μm XY radius	A.U.	$z_i^{\text{norm}} = \frac{z_i - \min(z_j)}{\max(z_j) - \min(z_j)}$
Distance to mask boundary	Distance_Cell_Mask	Distance from cell centroid to closest point on segmented tissue mask	μm	$d_{\text{mask}} = \min_{x \in \text{Mask}} \lVert \mathbf{p}_{\text{cell}} - x \rVert$
