# Supplementary material for: Single-cell morphodynamics predict cell fate decisions during mucociliary epithelial differentiation
Source: Mol Syst Biol. 2026 May 11;22(7):1040–69. doi: 10.1038/s44320-026-00212-x (PMC13328729; doi:10.1038/s44320-026-00212-x)
Supplement: Supplementary file 10 — Expanded View Figures [file 44320_2026_212_MOESM10_ESM.pdf]

# Expanded View Figures

**Figure EV1. Supplemental data for principal component analysis.**

(**A**) PCA feature spaces of dataset 1. (**B**) Individual cell trajectories in feature spaces fit for dataset 2. (**C**) Timewise positional features and individual trajectories' positional features for dataset 2. (**D**) All features' correlation with all feature PC1 and PC2.

© The Author(s)

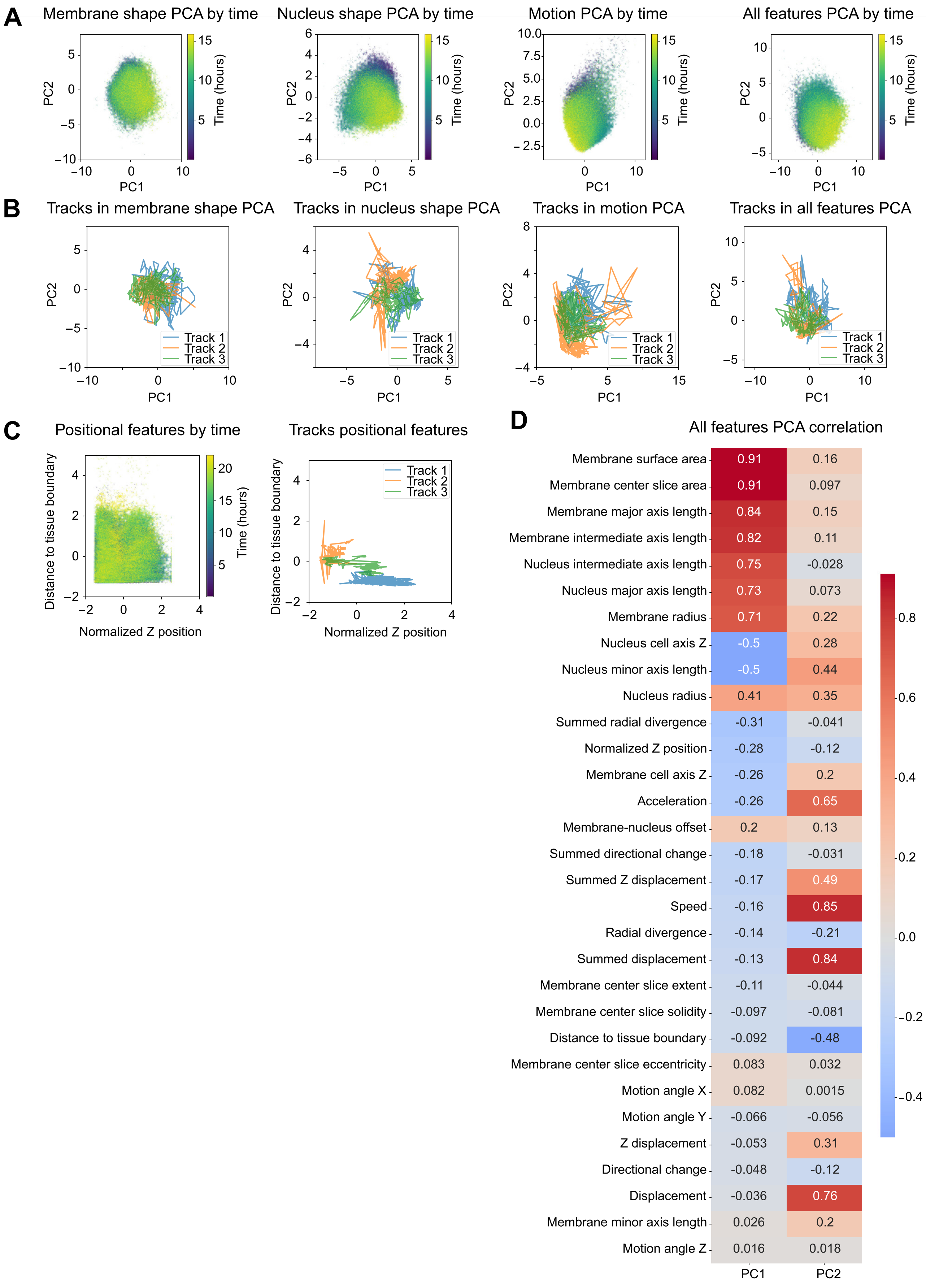
A
Membrane shape PCA by time
Nucleus shape PCA by time
Motion PCA by time
All features PCA by time
PC1
PC2
Time (hours)
B
Tracks in membrane shape PCA
Tracks in nucleus shape PCA
Tracks in motion PCA
Tracks in all features PCA
Track 1
Track 2
Track 3
C
Positional features by time
Tracks positional features
Distance to tissue boundary
Normalized Z position
D
All features PCA correlation
Membrane surface area 0.91 0.16
Membrane center slice area 0.91 0.097
Membrane major axis length 0.84 0.15
Membrane intermediate axis length 0.82 0.11
Nucleus intermediate axis length 0.75 -0.028
Nucleus major axis length 0.73 0.073
Membrane radius 0.71 0.22
Nucleus cell axis Z -0.5 0.28
Nucleus minor axis length -0.5 0.44
Nucleus radius 0.41 0.35
Summed radial divergence -0.31 -0.041
Normalized Z position -0.28 -0.12
Membrane cell axis Z -0.26 0.2
Acceleration -0.26 0.65
Membrane-nucleus offset 0.2 0.13
Summed directional change -0.18 -0.031
Summed Z displacement -0.17 0.49
Speed -0.16 0.85
Radial divergence -0.14 -0.21
Summed displacement -0.13 0.84
Membrane center slice extent -0.11 -0.044
Membrane center slice solidity -0.097 -0.081
Distance to tissue boundary -0.092 -0.48
Membrane center slice eccentricity 0.083 0.032
Motion angle X 0.082 0.0015
Motion angle Y -0.066 -0.056
Z displacement -0.053 0.31
Directional change -0.048 -0.12
Displacement -0.036 0.76
Membrane minor axis length 0.026 0.2
Motion angle Z 0.016 0.018
PC1
PC2

 © The Author(s)

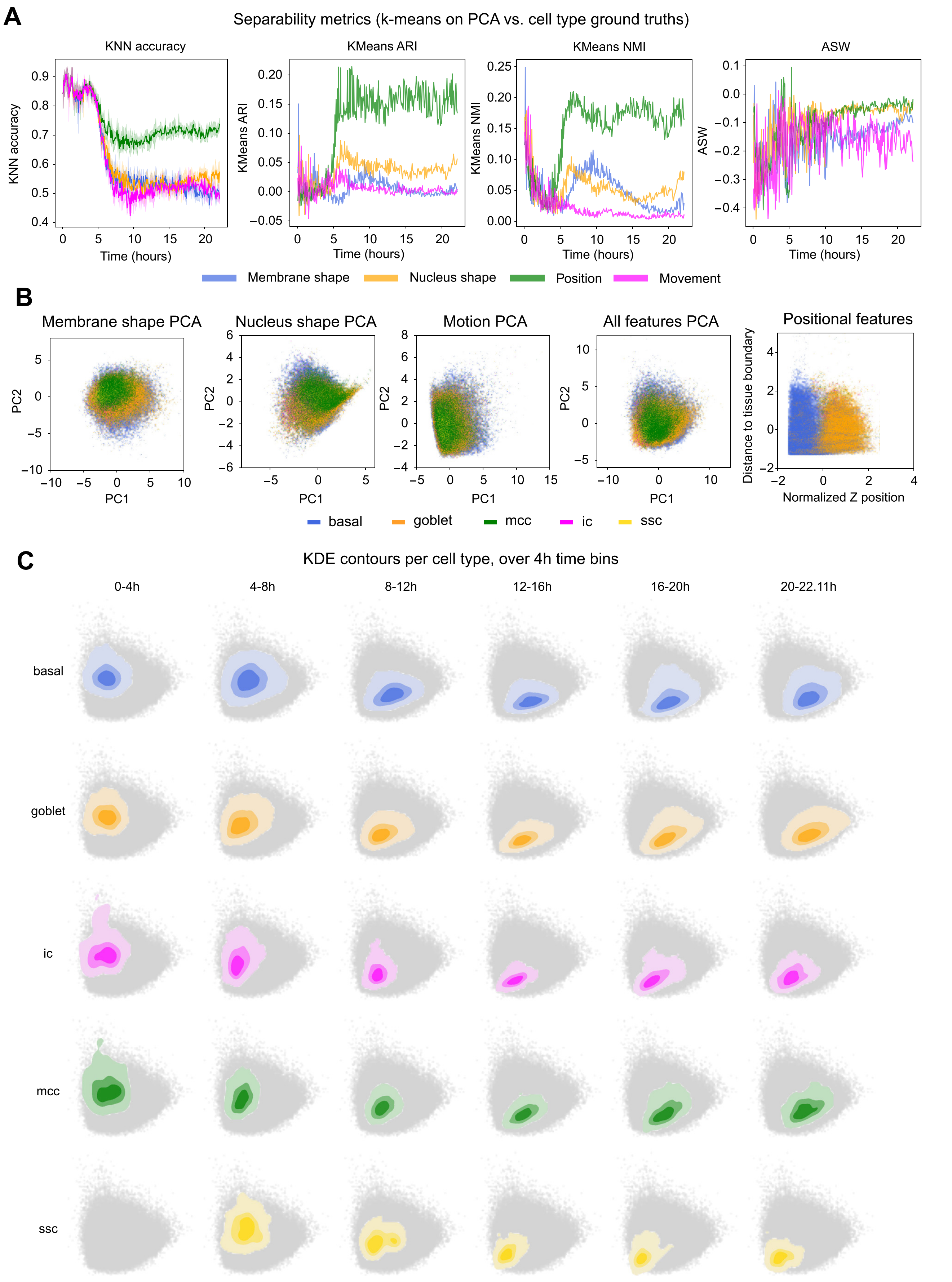
A
Separability metrics (k-means on PCA vs. cell type ground truths)
KNN accuracy
KMeans ARI
KMeans NMI
ASW
Time (hours)
Membrane shape
Nucleus shape
Position
Movement
B
Membrane shape PCA
Nucleus shape PCA
Motion PCA
All features PCA
Positional features
PC1
PC2
Distance to tissue boundary
Normalized Z position
basal
goblet
mcc
ic
ssc
C
KDE contours per cell type, over 4h time bins
0-4h
4-8h
8-12h
12-16h
16-20h
20-22.11h
basal
goblet
ic
mcc
ssc

© The Author(s) 

**Figure EV2. Backtracked cell types in PCA feature space.**

(**A**) Separability metrics for feature categories. Mean accuracy score (KNN accuracy) based on KNearestClassifier (scikit-learn) of different feature categories, where $k = 5$, Adjusted Rand Index score (KMeans ARI), Normalized Mutual Information score (KMeans NMI) for comparing k-means clustering of selected feature spaces (corresponding to (**B**)) with ground-truth cell type labels. Average silhouette width (ASW) is calculated for different feature categories against ground-truth cell-type labels. (**B**) Cell types labeled in feature spaces corresponding to Fig. 3D and Appendix Fig. S2C. (**C**) Kernel density estimation of time windowed cells in the all features PCA space, estimated per cell type.

 © The Author(s)

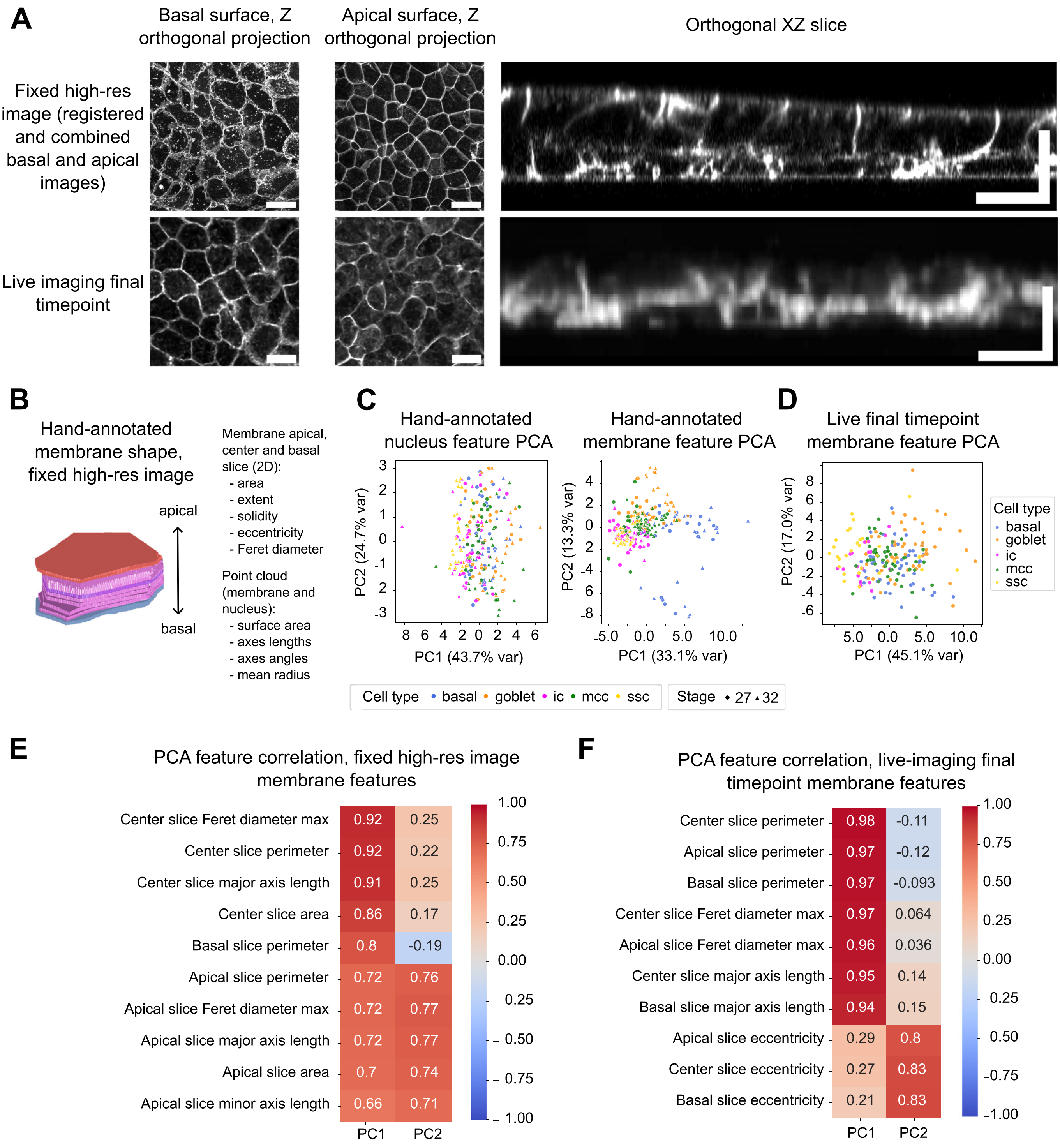


**Figure EV3. Comparison of high-resolution imaging and live imaging of the membrane signal and feature space of an extended shape feature set.**

(**A**) Top: Representative example of an animal cap region showing apical and basal Z-projections and an orthogonal XZ slice from the reconstructed high-resolution fixed-tissue dataset, generated by registration and combination of apical and basal image stacks. Bottom: representative example of apical and basal Z projections and orthogonal XZ slice from the final timepoint of a live-imaging experiment (Dataset 2). Scale bar: 30 µm. (**B**) Schematic of the extended membrane feature set used for hand-annotated cells and Live-imaging final timepoint comparison, including 2D membrane descriptors extracted from apical, central, and basal slices and 3D point-cloud–based descriptors for membrane and nucleus shapes. (**C**) Left: PCA of nuclear shape features from the same manually annotated fixed dataset, right: PCA of membrane shape features derived from manually annotated high-resolution fixed samples. (**D**) PCA of membrane shape features derived from automatically segmented cells at the final time point of live imaging (Dataset 2). (**E**, **F**) Correlation matrices of PCA loadings for membrane features from the fixed high-resolution dataset (**E**) and live-imaging final timepoint dataset (**F**).

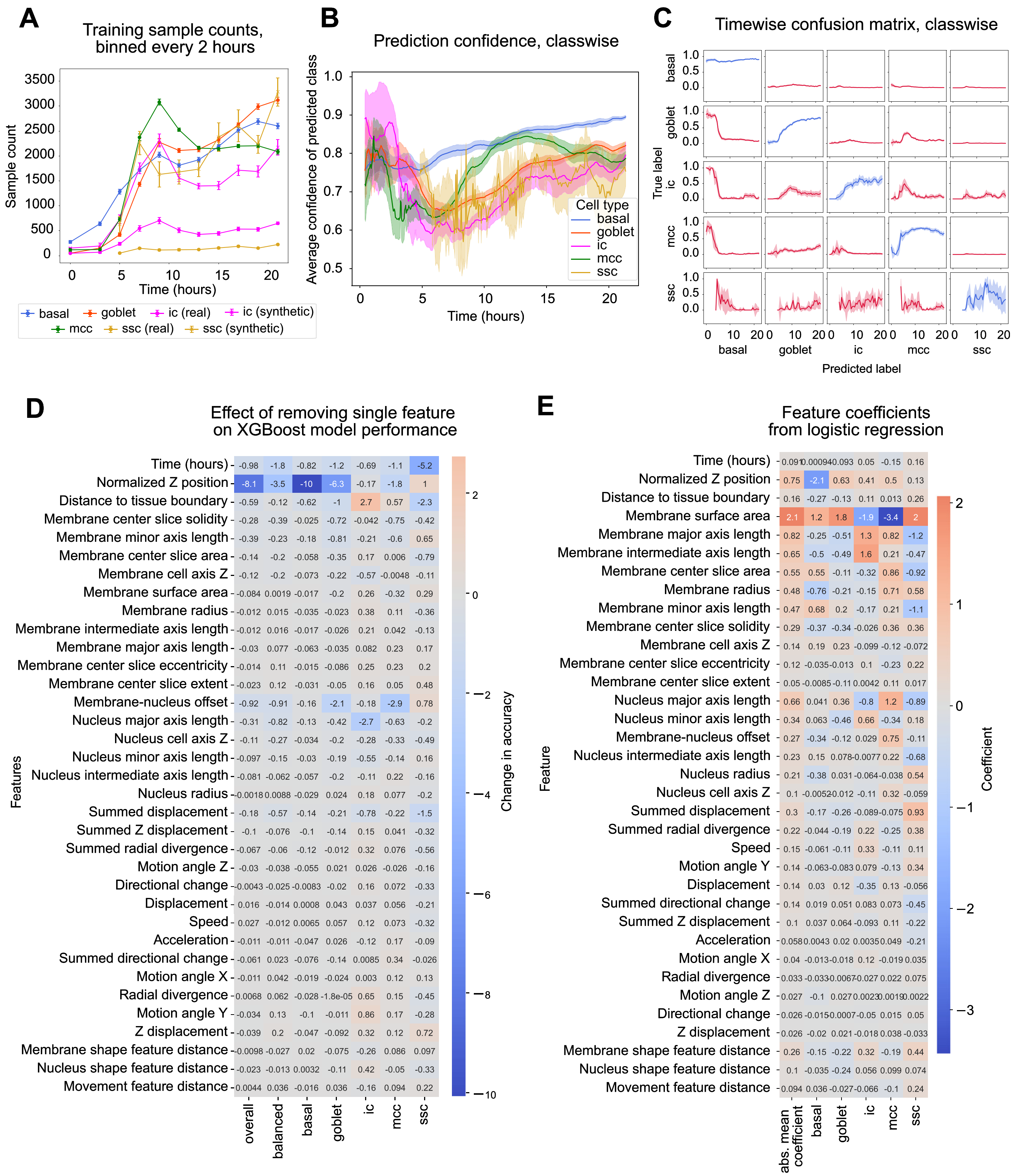
A
Training sample counts, binned every 2 hours
Sample count
Time (hours)
basal
goblet
ic (real)
ic (synthetic)
mcc
ssc (real)
ssc (synthetic)
B
Prediction confidence, classwise
Average confidence of predicted class
Time (hours)
Cell type
basal
goblet
ic
mcc
ssc
C
Timewise confusion matrix, classwise
True label
basal
goblet
ic
mcc
ssc
Predicted label
D
Effect of removing single feature on XGBoost model performance
Features
Change in accuracy
Time (hours)
Normalized Z position
Distance to tissue boundary
Membrane center slice solidity
Membrane minor axis length
Membrane center slice area
Membrane cell axis Z
Membrane surface area
Membrane radius
Membrane intermediate axis length
Membrane major axis length
Membrane center slice eccentricity
Membrane center slice extent
Membrane-nucleus offset
Nucleus major axis length
Nucleus cell axis Z
Nucleus minor axis length
Nucleus intermediate axis length
Nucleus radius
Summed displacement
Summed Z displacement
Summed radial divergence
Motion angle Z
Directional change
Displacement
Speed
Acceleration
Summed directional change
Motion angle X
Radial divergence
Motion angle Y
Z displacement
Membrane shape feature distance
Nucleus shape feature distance
Movement feature distance
overall
balanced
basal
goblet
ic
mcc
ssc
E
Feature coefficients from logistic regression
Feature
Coefficient
Time (hours)
Normalized Z position
Distance to tissue boundary
Membrane surface area
Membrane major axis length
Membrane intermediate axis length
Membrane center slice area
Membrane radius
Membrane minor axis length
Membrane center slice solidity
Membrane cell axis Z
Membrane center slice eccentricity
Membrane center slice extent
Nucleus major axis length
Nucleus minor axis length
Membrane-nucleus offset
Nucleus intermediate axis length
Nucleus radius
Nucleus cell axis Z
Summed displacement
Summed radial divergence
Speed
Motion angle Y
Displacement
Summed directional change
Summed Z displacement
Acceleration
Motion angle X
Radial divergence
Motion angle Z
Directional change
Z displacement
Membrane shape feature distance
Nucleus shape feature distance
Movement feature distance
abs. mean coefficient
basal
goblet
ic
mcc
ssc

 © The Author(s)

**Figure EV4. Classifier model performance analysis and feature importances.**

(**A**) Classifier training dataset sample counts across dataset time, binned every 2 h, mean across $n = 20$ independent iterations of randomized train–test sampling. Error bars represent the standard deviation (±s.d.) across iterations. For basal, goblet, and MCC classes, only real samples were used; for IC and SSC classes, some samples were synthesized using SMOTE oversampling (imbalanced-learn). (**B**) Mean and s.d. prediction confidence of predicted class of XGBoost-based predictions, colored by class. (**C**) Time-wise accuracy confusion matrix of XGBoost-based predictions. (**D**) Percent point-wise accuracy difference to baseline accuracy of the XGBoost model for leaving out a single feature. (**E**) Feature coefficients for a logistic regressor model. All calculations in (**B**–**E**) are averaged over 20 iterations of a randomized test–train split, model training, and prediction.

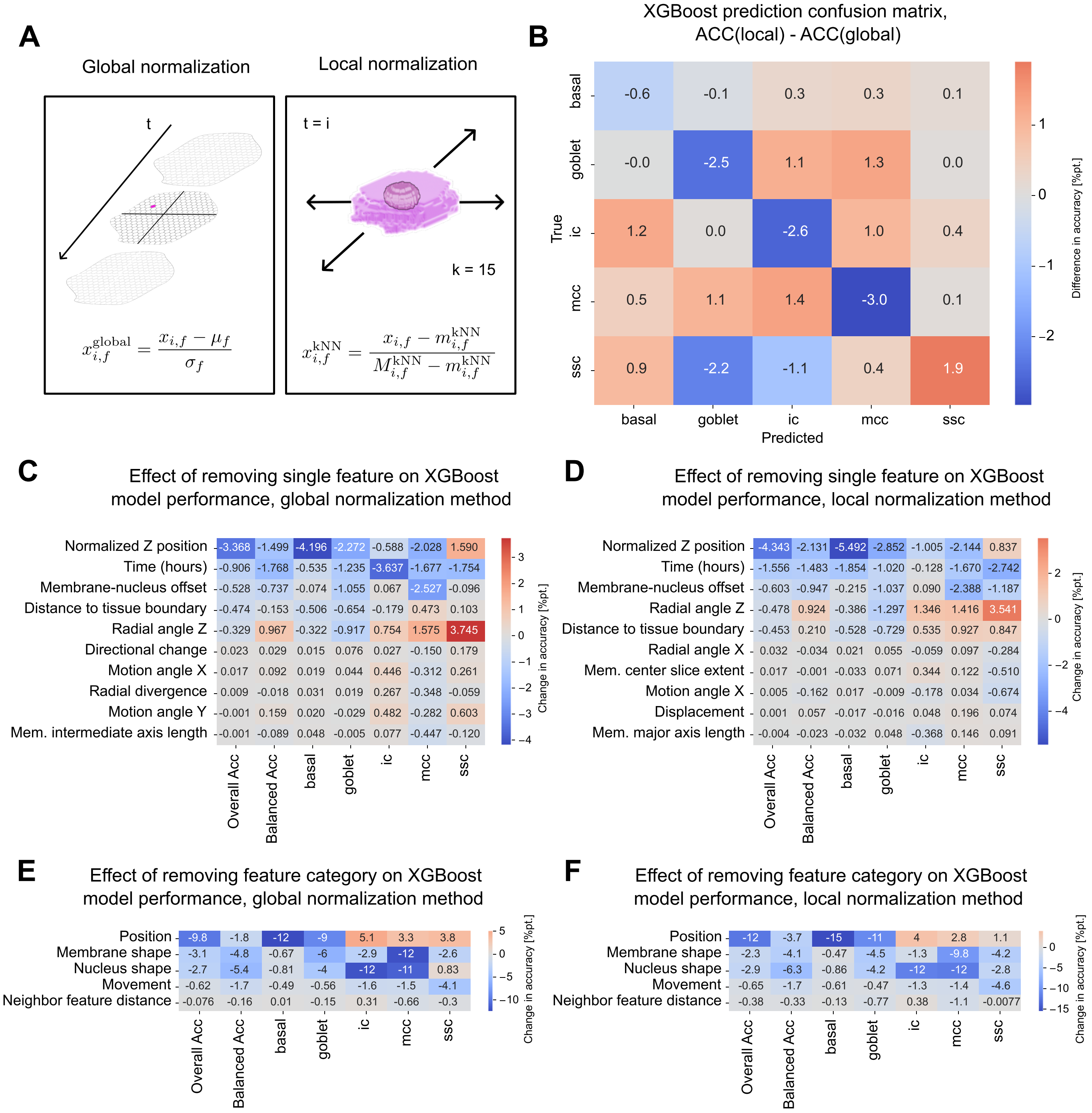


**Figure EV5. Effect of global versus local feature normalization on XGBoost model performance and feature importance.**

(**A**) Schematic depicting the compared normalization strategies. Left: In global normalization, cell features are standardized across all cells and all experimental timepoints. Right: In the local normalization strategy, $k = 15$ nearest neighbors in the cell experimental timepoint are used for min–max normalizing feature values. (**B**) Confusion matrix showing the difference in XGBoost prediction accuracy between the local and global normalization strategy. (**C, D**) Change in overall accuracy, balanced accuracy, and per cell-type accuracy upon removal of individual features under the global normalization strategy and under the local normalization strategy (**D**). The five highest- and lowest-accuracy features are shown in each heatmap. (**E, F**) Change in overall accuracy, balanced accuracy, and per cell-type accuracy upon removal of entire feature categories (position, membrane shape, nucleus shape, movement, neighbor feature distance) under the global normalization strategy (**E**) or the local normalization strategy (**F**).

 © The Author(s)
