# Supplementary material for: Single-cell morphodynamics predict cell fate decisions during mucociliary epithelial differentiation
Source: Mol Syst Biol. 2026 May 11;22(7):1040–69. doi: 10.1038/s44320-026-00212-x (PMC13328729; doi:10.1038/s44320-026-00212-x)
Supplement: Supplementary file 1 — Peer Review File [file 44320_2026_212_MOESM1_ESM.pdf]

molecular systems biology

Single-cell morphodynamics predict cell fate decisions during mucociliary epithelial differentiation

Mari Tolonen, Ziwei Xu, Ozgur Beker, Varun Kapoor, Bianca Dumitrascu, and Jakub Sedzinski

Corresponding author(s): Jakub Sedzinski (jakub.sedzinski@sund.ku.dk) , Bianca Dumitrascu (bmd2151@columbia.edu)

Review Timeline:

Submission Date:	17th Sep 25
Editorial Decision:	17th Oct 25
Revision Received:	2nd Mar 26
Editorial Decision:	2nd Apr 26
Revision Received:	10th Apr 26
Accepted:	16th Apr 26

Editor: Poonam Bheda

Transaction Report:

(Note: With the exception of the correction of typographical or spelling errors that could be a source of ambiguity, letters and reports are not edited. Depending on transfer agreements, referee reports obtained elsewhere may or may not be included in this compilation. Referee reports are anonymous unless the Referee chooses to sign their reports.)

17th Oct 2025

Manuscript Number: MSB-2025-13344
Title: Single-cell morphodynamics predict cell fate decisions during mucociliary epithelial differentiation

Dear Dr Sedzinski,

Thank you for the submission of your manuscript to Molecular Systems Biology. We have now received feedback from the two/three reviewers who agreed to evaluate your manuscript. As you will see from the reports below, the referees acknowledge the interest of the study and are overall supporting publication of your work pending appropriate revisions.

I think that the recommendations of the reviewers are rather clear and I therefore do not see the need to repeat the comments listed below. All issues raised would need to be satisfactorily addressed. Please let me know in case you would like to discuss in further detail any of the any of the reviewer comments or your proposed revisions, I would be happy to schedule a call.

When submitting your revised manuscript, please carefully review the instructions that follow below. We perform an initial quality control of all revised manuscripts before re-review; failure to include requested items will delay the evaluation of your revision.

We require:

1) A .docx formatted version of the manuscript text (including legends for main figures, EV figures and tables). Please make sure that the changes are highlighted to be clearly visible. Alternatively you may choose to submit your manuscript as a LaTeX file.

2) Individual production quality figure files as .eps, .tif, .jpg (one file per figure). For guidance, download the 'Figure Guide PDF' (https://www.embopress.org/page/journal/17574684/authorguide#figureformat).

3) At EMBO Press we ask authors to provide source data for the main figures. Our source data coordinator will contact you to discuss which figure panels we would need source data for and will also provide you with helpful tips on how to upload and organize the files.

4) A .docx formatted letter INCLUDING the reviewers' reports and your detailed point-by-point responses to their comments. As part of the EMBO Press transparent editorial process, the point-by-point response is part of the Peer Review File (PRF), which will be published alongside your paper.

5) A complete author checklist, which you can download from our author guidelines (https://www.embopress.org/page/journal/17574684/authorguide#submissionofrevisions). Please insert information in the checklist that is also reflected in the manuscript. The completed author checklist will also be part of the PRF.

6) Please note that all corresponding authors are required to supply an ORCID ID for their name upon submission of a revised manuscript.

7) It is mandatory to include a 'Data Availability' section after the Materials and Methods. Before submitting your revision, primary datasets produced in this study need to be deposited in an appropriate public database, and the accession numbers and database listed under 'Data Availability'. Please remember to provide a reviewer password if the datasets are not yet public (see https://www.embopress.org/page/journal/17574684/authorguide#dataavailability).

In case you have no data that requires deposition in a public database, please state so in this section as follows: "This study includes no data deposited in external repositories". Note that the Data Availability Section is restricted to new primary data that are part of this study.

8) All Materials and Methods need to be described in the main text using our 'Structured Methods' format, which is required for all research articles. According to this format, the Methods section includes a Reagents and Tools Table (listing key reagents, experimental models, software and relevant equipment and including their sources and relevant identifiers) followed by a Methods and Protocols section describing the methods using a step-by-step protocol format. The aim is to facilitate adoption of the methodologies across labs. Please upload the Reagents and Tools table as a separate document when submitting your revised manuscript. More information on how to adhere to this format as well as a downloadable template (.docx) for the Reagents and Tools Table can be found in our author guidelines:
https://www.embopress.org/page/journal/17444292/authorguide#structuredmethods

An example of a Method paper with Structured Methods can be found here:
https://www.embopress.org/doi/10.15252/msb.20178071.

9) For data quantification: please specify the name of the statistical test used to generate error bars and p-values, the number (n) of independent experiments (specify technical or biological replicates) underlying each data point and the test used to calculate p-values in each figure legend. The figure legends should contain a basic description of n, p-values and the test applied. Graphs must include a description of the bars and the error bars (s.d., s.e.m.). Please provide exact p-values (in either the figure or figure legend).

10) Our journal encourages inclusion of *data citations in the reference list* to directly cite datasets that were re-used and obtained from public databases. Data citations in the article text are distinct from normal bibliographical citations and should directly link to the database records from which the data can be accessed. In the main text, data citations are formatted as follows: "Data ref: Smith et al, 2001" or "Data ref: NCBI Sequence Read Archive PRJNA342805, 2017". In the Reference list, data citations must be labeled with "[DATASET]". A data reference must provide the database name, accession number/identifiers and a resolvable link to the landing page from which the data can be accessed at the end of the reference. Further instructions are available at .

11) We replaced Supplementary Information with Expanded View (EV) Figures and Tables that are collapsible/expandable online. EV Figures should be cited as 'Figure EV1, Figure EV2" etc... in the text and their respective legends should be included in the main text after the legends of regular figures.
- For the figures that you do NOT wish to display as Expanded View figures, they should be bundled together with their legends in a single PDF file called *Appendix*, which should start with a short Table of Content. Appendix figures should be referred to in the main text as: "Appendix Figure S1, Appendix Figure S2" etc.
- Additional Tables/Datasets should be labeled and referred to as Table EV1, Dataset EV1, etc. Legends should be provided in a separate tab in case of .xls files. Alternatively, the legend can be supplied as a separate text file (README) and zipped together with the Table/Dataset file.
See detailed instructions here:
https://www.embopress.org/page/journal/17574684/authorguide#expandedview

12) Author contributions: CRediT has replaced the traditional author contributions section because it offers a systematic machine-readable author contributions format that allows for more effective research assessment. Please remove the Authors Contributions from the manuscript and use the free text boxes beneath each contributing author's name in our system to add specific details on the author's contribution. More information is available in our guide to authors.

13) Disclosure statement and competing interests: We updated our journal's competing interests policy in January 2022 and request authors to consider both actual and perceived competing interests. Please review the policy https://www.embopress.org/competing-interests and update your competing interests if necessary.

14) Every published paper now includes a 'Synopsis' to further enhance discoverability. Synopses are displayed on the journal webpage and are freely accessible to all readers. They include a short stand first (maximum of 300 characters, including space) as well as 2-5 one-sentences bullet points that summarizes the paper. Please write the bullet points to summarize the key NEW findings. They should be designed to be complementary to the abstract - i.e. not repeat the same text. We encourage inclusion of key acronyms and quantitative information (maximum of 30 words / bullet point). Please use the passive voice. Please attach these in a separate file or send them by email, we will incorporate them accordingly.

Please also suggest a striking image or visual abstract to illustrate your article as a PNG file 550 px wide x 300-600 px high.

Please note that these would be the final versions and changes during proofing are usually not allowed.

15) As part of the EMBO Publications transparent editorial process initiative (see our policy here: https://www.embopress.org/transparent-process#Review_Process), Molecular Systems Biology will publish online a Peer Review File (PRF) to accompany accepted manuscripts.
In the event of acceptance, this file will be published in conjunction with your paper and will include the anonymous referee reports, your point-by-point response and all pertinent correspondence relating to the manuscript. Let us know whether you agree with the publication of the PRF and as here, if you want to remove or not any figures from it prior to publication.
Please note that the Author checklist will be published at the end of the PRF.

Molecular Systems Biology has a "scooping protection" policy, whereby similar findings that are published by others during review or revision are not a criterion for rejection. Should you decide to submit a revised version, I do ask that you get in touch after three months if you have not completed it, to update us on the status.

Yours sincerely,

Poonam Bheda, PhD
Scientific Editor
Molecular Systems Biology

Reviewer #1:

The manuscript from Tolonen et al. addresses the exciting challenges and advances that are associated with using cell shape or morphodynamics to predict cell fate in a complex developing epithelium. They utilize the "simplified" complex tissue of the mucociliated epithelium of the developing Xenopus embryo that can be excised and cultured in vitro allowing for high-quality long-term imaging. Importantly this tissue contains multiple distinct cell types for which they have good markers. Using this system they generate movies of tissue development and use a wide range of sophisticated analysis to track the cell size, shape, nuclear size and shape as well as overall movement. To do this they had to develop and integrate a number of new computational analyses including tracking individual cells over a long-time range when cell divisions are constantly occurring. This paper is a tour de force that addresses an important and timely question and the manuscript is extremely well written making a complex analysis very digestible. Additionally it provides the community with some very useful computational tools for 3D segmentation and tracking. They perform an impressive number of comparative analyses and generate a pipeline that is quite competent at identifying certain cell types. Importantly, the authors do a particularly good job of not overstating their findings and weaving the challenges and advances throughout the paper. In a day when too many labs overstate their findings the authors should be commended for the honest portrayal of their overall process. While they have clearly not solved everything related to morphodynamics or phenomics this paper represents an impressive advance in the field that will be of interest to a wide range of groups addressing similar questions. I have very few concerns and am supportive of publication.

Comments:

While I do not doubt the model at all, it would be nice to have an end point comparison of cell shapes in explants versus embryos.

I was under the impression that MCCs were quite distinct in 3D shape having a bit of a "pear shape" relative to the other cells. This does not come across in the morphometric analysis (e.g. 4E). Is this not true or is it less striking in the explained tissue?

The similarities in cell movement suggests that all cells are swept up in the global movement of the tissue. Could useful information be gleaned from normalizing the movement of MCCs, ICs, and SCCs relative to the general morphogenic movements as measured from goblets cells and/or basal cells?

Minor:
In figure 1C it would be nice for the image to label some of the cells.

While it was fairly easy to guess, in figure S1 it was not defined what tp, fn or fp were referring to. Please specify.

In numerical descriptions a , and . were often swapped out. Just be consistent. E.g.... (PC1: 17.6%, PC2: 10.7%, PC3: 8,9%).

Reviewer #2:

Summary

In this article, Tolonen and colleagues introduce a novel computational and experimental framework to analyze cell state transitions. This work introduces a morphodynamics analysis pipeline based on live-cell imaging and machine learning which aims to determine cell fate based on the single cell features extracted by the pipeline. It uses the differentiation of the Xenopus mucociliary epithelium as a model, a rapidly developing tissue composed of multiple specialized cell types.

The authors segmented and tracked individual cells over time in a live 3D tissue, quantifying dynamic features such as cell shape, nuclear morphology, movement, and spatial positioning. At the end of a live microscopy experiment, the sample is fixed and immunostaining markers are used to assign the true cell types to cells. By backtracking to live imaging data, they can assess the predictive power of the morphodynamics features they measured, and at what time they are determinant.

Despite high variability in individual features, supervised machine learning models successfully predicted cell fate with moderate accuracy, especially for abundant cell types like basal, goblet, and multiciliated cells. Key discriminative features included normalized Z-position, membrane-nucleus offset, and experimental time, while cell shape and movement features contributed minimally to prediction power.

The study highlights the challenges of distinguishing cell types based solely on instantaneous morphodynamic features, as cells

in densely packed tissues exhibit overlapping and noisy phenotypic profiles. The authors note that such 3D, densely packed samples, are more challenging for this approach than 2D cell cultures. By leveraging supervised learning and trajectory data, the researchers were able to extract meaningful patterns in morphodynamics that predict or at least correlate with final cell fate.

General remarks

The article is really well written and well articulated. The findings heavily rely on mastering a wide range of methods, from live tissue imaging to image processing and machine learning. Importantly, their choice is properly justified and the reasoning behind what prompted them is transparent in the paper. The image data is not readily available (only after request) but the image analysis and machine learning pipelines are open source and reasonably documented publicly on github. The images and movies are stunning. The figures are well organized and readable. I only have two main comments and a few minor points. I can only recommend the acceptance of this article.

Main comments

1/ 3D segmentation inaccuracies might hide morphology features importance

The findings are that morphodynamics alone are partly predictive in this tissue. In particular the authors find that cell shape descriptors have a lower feature importance than Z-position, time and membrane-nucleus offset. This is discussed in the article, particularly in the light of similar approaches in 2D cell cultures, where shape-based profiling achieves a very good predictive power. The authors stress that in the 3D tissues, the cells are densely packed, and hence, their shape appear all similar, and that in light microscopy, the resolution along the Z axis is limited.

However the low importance of shape features - in this paper and in others studying 3D samples - might also be the consequence of 3D segmentation inaccuracies. As noted by the authors, 3D cell segmentation in dense tissues is especially difficult and the tools currently available can yield results that are a far cry from what we have in 2D. There is the possibility that some morphological features are important for cell fate prediction, but that their importance is hidden by widely imperfect segmentation.

For instance, the authors elected to perform 3D cell segmentation in a slice by slice manner. Each 2D Z-slice is segmented with cellpose, then the resulting 2D masks are stitched together. This approach is effective but in our experience generates segmentation masks that have a pronounced staircase pattern (visible in this work in e.g. Supplemental Movie 7), and do not properly segment the cell at its poles. Indeed, the authors find a lower segmentation quality for cell segmentation than for nucleus segmentation, with a Jaccard index around 50%. Could this be enough to drown an important morphological feature in noise?

The article mentions this possibility. In the last part of the Results section, the authors note that nuclear shape features are more informative than cell shape features, and that this is likely the cause of a better segmentation fidelity of the former. It would be beneficial for the authors to address this point further in the Discussion section. Additionally, authors could visually assess the accuracy of cell segmentation by comparing it directly to the actual cell membrane signal. Such a problem affects all 3D studies but to our knowledge is seldom discussed. In the section titled "Segmentation and tracking pipeline optimized for developing MCE," the authors state: "Despite these limitations, segmentation accuracy was sufficient to support reliable quantification of cell shape and dynamics for downstream analyses." However, this claim would be strengthened by providing quantitative or qualitative evidence to substantiate it.

2/ Importance of relative cell movement features

The cell movement features seem to be all measured with respect to the global coordinate system (absolute displacement, speed, etc). Inasmuch, the authors state that the movement features contributed little to cell fate prediction, likely due to collective migration within the tissue.

But what about the relative cell movement, with respect to the collective migration? If we define a local tissue movement on the scale of about 15 cells, do we have single cell movements, measurement relative to this local tissue movement, that could be predictive?

Minor points

- In the 'Comparisons to related approaches', I could not help thinking of an article which use backtracking in a similar manner to

predict the fate of infected cells:
https://www.biorxiv.org/content/10.1101/2024.10.28.620606v1

- In the Materials and Methods section, the Membrane segmentation paragraph, I suggest replacing "using their 'cyto2' model" with "using the 'cyto2' model".

Reviewer #3:

Tolonen and colleagues present in the manuscript "Single-cell morphodynamics predict cell fate decisions during mucociliary epithelial differentiation" a novel analysis pipeline to facilitate omics-scale morphometric analysis of the mucociliary epithelium of developing frog epidermis. They developed long-term live cell imaging of the explanted epithelium, nuclei and membrane segmentation approaches, a predictive algorithm to identify and track cell divisions as well as approaches to extract morphological and motility features of tracked cells over developmental time. Then they use computational approaches to comprehensively analyze morphodynamics of cells and cell groups within the datasets. Finally, they use some "classic" and some machine learning approaches to use morphodynamic features for cell fate prediction aided by end-point IF-based assignment of cell identities and backtracking of cells towards their origin during epithelium development. While the prediction accuracy varies across cell types in the epithelium and some cell types remain hard to distinguish from each other, the overall approach and pipeline are impressive and will be useful for a potentially large group of researchers moving forward. Interestingly, one key message of the presented work is that within complex tissues in an in vivo like state (such as epidermis explants used here), cell morphology and behavior seem to carry limited predictive potential, likely due to aspects regarding cell packing as well as similarities of cell behaviors during early phases of development (where they rather behave like a collective than distinguishable and separated groups of fated cells). This is an important piece of information, particularly in comparison to more artificial in vitro and organoid work published where morphology and cell behaviors can be used in some cases to predict cell identities. Overall, this is impressive work, presented and illustrated well. The methods will be very useful for a large audience of readers and the overall conclusions provide important new insights regarding setup, use and interpretation of omics-style morphometric analyses.
There are some remaining aspects that will require the author's attention before publication, but I am optimistic that they will be able to address the following remarks during revision. These remarks are pointed towards the biological aspects and context of the work rather than the computational approach, since I am not a specialist regarding the latter.

Major points:
Developmental lineages, cell positions and behaviors have been described in the model system by the authors and other groups. However, little of that knowledge has been used to contextualize the morphometric analysis, and few studies are even cited and taken into consideration at all. Expanding on that front could improve this work and the interpretation of data in form of hypothesis testing.
1.) Particularly, it is known that goblet cells are developing from the outer layer while basal and intercalating cells are generated from deep layer epithelial cells. This likely affects the predictability of cell fates, which is best for these goblet cells as well as basal cells that are born and remain in the deep layer. However, this aspect (where these cells are "born") is largely omitted. REF: e.g. PMID: 16728476, PMID: 28095645, PMID: 10518489
2.) Furthermore, it has been described that MCCs engage in active migration-repulsion behavior in vivo to facilitate even spacing of MCCs across the epithelium, which clearly distinguishes MCCs from other intercalating cell types. However, neither is the study cited, nor is this aspect tested or discussed although MCCs seem even spaced as shown in Fig. 4B. REF: PMID: 33756121
3.) After intercalation, MCCs undergo extensive and active apical surface expansion, while in ICs and SSCs the apical surface size remains relatively large, and goblet cells derived from the outer layer show a large surface area from the very beginning. Again, this seems so obvious to analyze for this reviewer, that it is a surprise it has not been addressed in more detail. REF: e.g. PMID: 26766441, PMID: 24598162
4.) The epidermis shows a remarkable degree of consistent cell type proportionality, where MCCs, ICs and SSCs are produced in approximately equal numbers. However, it seems like ICs and SSCs are relatively reduced in numbers as compared to MCCs in this system. Is this a problem of cell type identification or is this a result of the culture conditions? And how might this impact the analysis and interpretation overall? REF: e.g. PMID: 40086446, PMID: 30151772, PMID: 31553905

Additionally, there are some things that seem to require clarification:
1.) What happens in the analysis when cell divisions are not captured correctly? Are these cells/objects excluded? Same true for other analysis problems/artifacts (e.g. bad segmentation of membranes, unclear cell identities, etc.)? It is mentioned that only 33% of cells were successfully tracked, how does that affect analysis and what happens to the 66% of information lost?
2.) It is unclear to me which embryonic stages match the stages of the explant development. 22h (length of the movie) after st. 8/9 cutting explants seems a bit short for full development of the epidermis. Please clarify if embryos were used to stage match explants or how this was determined.
3.) It is not fully clear to me if time-overlapping movies were taken to generate full developmental time coverage. Please clarify.

4.) The authors mention that cells undergoing cell division maintain cell fates and both daughter cells have been assigned the same identity in the analysis. But this is followed by "data not shown" statement. Please explain and provide the data or rather remove from the manuscript.

Minor points:
1.) Top page 3: The authors state that ECM stiffness regulates cell fates "independent" of genetic information, while I would argue that it regulates it in cooperation with genetic information and status of the cells.
2.) The authors use MCE for mucociliary epithelium throughout the manuscript. I would suggest to be specific and rename to mucociliary epidermis, as there are additional mucociliary epithelia in the organism.
3.) Page 5, first paragraph of results: The authors state that explants were cut from "superficial ectoderm". This term is confusing and suggests that only an apical portion has been used. However, it is the animal located ectoderm that is removed for culture.
4.) The authors use the term ground truth a lot. Please specify in the given contexts what is specifically meant (from my reading I could only place cell type identities derived by IF as one such ground truth, but in other cases I wasn't sure what they meant).

We thank the reviewers for their thoughtful and enthusiastic comments, which have substantially improved the clarity and overall quality of the revised manuscript. In response to their suggestions, we have clarified several key aspects of our work and revised the text describing the morphodynamics of cell fate acquisition to more clearly articulate the biological implications of our findings. We believe these revisions have strengthened the study's conceptual coherence. Below, we provide an itemized response to each comment, and all corresponding revisions are clearly marked in the manuscript.

Reviewer #1

The manuscript from Tolonen et al. addresses the exciting challenges and advances that are associated with using cell shape or morphodynamics to predict cell fate in a complex developing epithelium. They utilize the "simplified" complex tissue of the mucociliated epithelium of the developing Xenopus embryo that can be excised and cultured in vitro allowing for high-quality long-term imaging. Importantly this tissue contains multiple distinct cell types for which they have good markers. Using this system they generate movies of tissue development and use a wide range of sophisticated analysis to track the cell size, shape, nuclear size and shape as well as overall movement. To do this they had to develop and integrate a number of new computational analyses including tracking individual cells over a long-time range when cell divisions are constantly occurring. This paper is a tour de force that addresses an important and timely question and the manuscript is extremely well written making a complex analysis very digestible. Additionally it provides the community with some very useful computational tools for 3D segmentation and tracking. They perform an impressive number of comparative analyses and generate a pipeline that is quite competent at identifying certain cell types. Importantly, the authors do a particularly good job of not overstating their findings and weaving the challenges and advances throughout the paper. In a day when too many labs overstate their findings the authors should be commended for the honest portrayal of their overall process. While they have clearly not solved everything related to morphodynamics or phenomics this paper represents an impressive advance in the field that will be of interest to a wide range of groups addressing similar questions. I have very few concerns and am supportive of publication.

We thank the reviewer for their very positive and encouraging comments. We are pleased that they found our work clear, rigorous, and of broad interest to the field.

1. While I do not doubt the model at all, it would be nice to have an end point comparison of cell shapes in explants versus embryos.

We thank the reviewer for this helpful and constructive suggestion. In response, we have now included an endpoint comparison of cell and nuclear morphology between explants and embryos at different developmental stages, assessed from both the apical and basal sides of the tissue (new Appendix Figure S1). This analysis confirms that the explant system faithfully recapitulates the overall *in vivo* tissue architecture and cellular morphology.

While this comparison is qualitative, the gross tissue organization, cell shape, and nuclear morphology are highly comparable between the two systems.

We are currently conducting a detailed quantitative spatiotemporal analysis comparing animal cap explants and *in vivo* tissue to systematically align morphodynamic features and developmental timing between the two systems. Given the scope of this analysis, we believe it warrants a dedicated study, and we would be pleased to report these results in a future publication.

2. I was under the impression that MCCs were quite distinct in 3D shape having a bit of a "pear shape" relative to the other cells. This does not come across in the morphometric analysis (e.g. 4E). Is this not true or is it less striking in the explained tissue?

We thank the reviewer for this insightful comment. Indeed, *in vivo* multiciliated cells (MCCs) display a characteristic pear-shaped three-dimensional morphology. In our explant preparations, this morphology remains present but appears less pronounced, likely due to partial flattening of the tissue during culture and imaging.

In addition, our imaging is performed using an inverted microscope, which requires imaging through the basal cell layer. The pear-shaped geometry is most evident during radial intercalation, when MCCs transition from the inner layer to the outer epithelium and exhibit a broadened basal region together with a comparatively narrower apical domain prior to full apical expansion. However, due to light scattering in the highly pigmented and vesicle-rich tissue, we are unable to reliably image the full thickness of the epithelium, particularly at early developmental stages. Consequently, during radial intercalation, we cannot consistently resolve the entire MCC volume, especially the apical domain that contributes most prominently to the classic “pear-like” appearance.

Therefore, while MCCs do undergo the expected apico-basal morphological remodeling, the full 3D asymmetry is partially attenuated in our dataset and may not be fully captured by the reconstructed volumetric segmentations. We have now clarified this limitation in the “*Limitations and Data Constraints*” section supported by a new Figure EV3A, and additionally addressed this point in our response to Reviewer 3 (Point 3).

3. The similarities in cell movement suggests that all cells are swept up in the global movement of the tissue. Could useful information be gleaned from normalizing the movement of MCCs, ICs, and SCCs relative to the general morphogenic movements as measured from goblets cells and/or basal cells?

We thank the reviewer for this excellent and constructive suggestion. Indeed, the patterned nature of the MCE suggests that local differences in measured features (not only movement features) could reveal differences between cell types. In response, we explored this possibility by

normalizing each cell's features to its local neighborhood to capture local differences among cells (new Figure EV5).

Specifically, we performed both global and local normalisation of all cell features and applied supervised classification with XGBoost, similarly to Figure 5. When comparing the confusion matrices and feature importances, both approaches yielded consistent results. Importantly, even after applying either local or global normalisation, movement-related features contributed only minimally to cell fate determination.

These results are now presented in a new Extended View Figure (Figure EV5), and the analysis has been incorporated into the main text. We have also expanded the Methods section to provide a detailed description of both normalisation procedures.

4. In figure 1C it would be nice for the image to label some of the cells.

We thank the reviewer for this helpful suggestion. We have now added labels identifying the major cell types in Figure 1C to improve clarity.

5. While it was fairly easy to guess, in figure S1 it was not defined what tp, fn or fp were referring to. Please specify.

We thank the reviewer for noticing this omission. We have now defined *tp*, *fp*, and *fn* (true positive, false positive, and false negative, respectively) in the legend of Appendix Figure S3 (previously Figure S1).

6. In numerical descriptions a , and . were often swapped out. Just be consistent. E.g.... (PC1: 17.6%, PC2: 10.7%, PC3: 8,9%).

We thank the reviewer for pointing this out. We have carefully checked the manuscript and corrected all instances to ensure consistent use of decimal points.

Reviewer #2

In this article, Tolonen and colleagues introduce a novel computational and experimental framework to analyze cell state transitions. This work introduces a morphodynamics analysis pipeline based on live-cell imaging and machine learning which aims to determine cell fate based on the single cell features extracted by the pipeline. It uses the differentiation of the Xenopus mucociliary epithelium as a model, a rapidly developing tissue composed of multiple specialized cell types.

The authors segmented and tracked individual cells over time in a live 3D tissue, quantifying dynamic features such as cell shape, nuclear morphology, movement, and spatial positioning. At the end of a live microscopy experiment, the sample is fixed and immunostaining markers are used to assign the true cell types to cells. By backtracking to live imaging data, they can assess the predictive power of the morphodynamics features they measured, and at what time they are determinant.

Despite high variability in individual features, supervised machine learning models successfully predicted cell fate with moderate accuracy, especially for abundant cell types like basal, goblet, and multiciliated cells. Key discriminative features included normalized Z-position, membrane-nucleus offset, and experimental time, while cell shape and movement features contributed minimally to prediction power.

The study highlights the challenges of distinguishing cell types based solely on instantaneous morphodynamic features, as cells in densely packed tissues exhibit overlapping and noisy phenotypic profiles. The authors note that such 3D, densely packed samples, are more challenging for this approach than 2D cell cultures. By leveraging supervised learning and trajectory data, the researchers were able to extract meaningful patterns in morphodynamics that predict or at least correlate with final cell fate.

The article is really well written and well articulated. The findings heavily rely on mastering a wide range of methods, from live tissue imaging to image processing and machine learning. Importantly, their choice is properly justified and the reasoning behind what prompted them is transparent in the paper. The image data is not readily available (only after request) but the image analysis and machine learning pipelines are open source and reasonably documented publicly on github. The images and movies are stunning. The figures are well organized and readable. I only have two main comments and a few minor points. I can only recommend the acceptance of this article.

We thank the reviewer for their very positive and encouraging evaluation of our work. We greatly appreciate the recognition of the methodological breadth, clarity of presentation, and transparency of our approach. We are pleased that the reviewer found the manuscript well written, with clear and informative analyses and figures.

1. 3D segmentation inaccuracies might hide morphology features importance

The findings are that morphodynamics alone are partly predictive in this tissue. In particular the authors find that cell shape descriptors have a lower feature importance than Z-position, time and membrane-nucleus offset. This is discussed in the article, particularly in the light of similar approaches in 2D cell cultures, where shape-based profiling achieves a very good predictive

power. The authors stress that in the 3D tissues, the cells are densely packed, and hence, their shape appear all similar, and that in light microscopy, the resolution along the Z axis is limited.

However the low importance of shape features - in this paper and in others studying 3D samples - might also be the consequence of 3D segmentation inaccuracies. As noted by the authors, 3D cell segmentation in dense tissues is especially difficult and the tools currently available can yield results that are a far cry from what we have in 2D. There is the possibility that some morphological features are important for cell fate prediction, but that their importance is hidden by widely imperfect segmentation.

For instance, the authors elected to perform 3D cell segmentation in a slice by slice manner. Each 2D Z-slice is segmented with cellpose, then the resulting 2D masks are stitched together. This approach is effective but in our experience generates segmentation masks that have a pronounced staircase pattern (visible in this work in e.g. Supplemental Movie 7), and do not properly segment the cell at its poles. Indeed, the authors find a lower segmentation quality for cell segmentation than for nucleus segmentation, with a Jaccard index around 50%. Could this be enough to drown an important morphological feature in noise?

The article mentions this possibility. In the last part of the Results section, the authors note that nuclear shape features are more informative than cell shape features, and that this is likely the cause of a better segmentation fidelity of the former. It would be beneficial for the authors to address this point further in the Discussion section. Additionally, authors could visually assess the accuracy of cell segmentation by comparing it directly to the actual cell membrane signal. Such a problem affects all 3D studies but to our knowledge is seldom discussed. In the section titled "Segmentation and tracking pipeline optimized for developing MCE," the authors state: "Despite these limitations, segmentation accuracy was sufficient to support reliable quantification of cell shape and dynamics for downstream analyses." However, this claim would be strengthened by providing quantitative or qualitative evidence to substantiate it.

We thank the reviewer for this excellent and thoughtful comment. We fully agree that segmentation inaccuracies in 3D tissues can obscure or underestimate the contribution of morphological features. We have therefore expanded the “*Limitations and Data Constraints*” section to explicitly address this limitation and its potential impact on the relative contribution of cell shape descriptors.

To directly address the reviewer’s concern that imperfect membrane segmentation could mask biologically relevant morphological features, we performed high-resolution imaging of fixed tissue at fully differentiated developmental stages, corresponding to the end time point of our live-imaging experiments. The tissue was imaged from both the apical and basal sides, and the two views were computationally aligned to reconstruct the full volumetric architecture.

In this dataset, we manually annotated 50 cells for each cell type and quantified a comprehensive set of membrane- and nucleus-based morphological descriptors. We then projected these features into the PCA space to determine whether high-resolution volumetric morphology could better separate cell identities than features extracted from the final frame of live imaging.

Interestingly, although separation of cell identities was modestly improved in the high-resolution dataset, particularly for basal cells, the overall populations remained largely overlapping. The PCA feature correlation matrix further revealed that, in high-resolution images, PC1 is predominantly driven by central slice features and PC2 by apical features. In contrast, in the lower-resolution live dataset, feature contributions to PC1 and PC2 are more mixed. This difference is consistent with the improved spatial fidelity of the fixed high-resolution imaging.

Importantly, however, even with high-resolution volumetric segmentation, membrane and nuclear morphological features alone were insufficient to robustly separate cell identities. These findings therefore strengthen our conclusion that dynamic morphological trajectories over time, rather than static endpoint morphology, are critical for distinguishing cell fate.

We have now included this comparison between high-resolution fixed imaging and live imaging in a new Extended View Figure (Figure EV3) and have described the corresponding analysis in the revised manuscript.

2. Importance of relative cell movement features

The cell movement features seem to be all measured with respect to the global coordinate system (absolute displacement, speed, etc). Inasmuch, the authors state that the movement features contributed little to cell fate prediction, likely due to collective migration within the tissue.

But what about the relative cell movement, with respect to the collective migration? If we define a local tissue movement on the scale of about 15 cells, do we have single cell movements, measurement relative to this local tissue movement, that could be predictive?

We thank the reviewer for this insightful suggestion. In line with the related comment from Reviewer 1, we have now compared global and local normalisation of cell features to reflect local feature differences, including movement features, in the classification performance. Specifically, we used the same train-test datasets with features normalized with two different strategies: (i) a globally normalized representation, where the cell's features are normalized to all cells in all experimental timepoints, and (ii) a locally normalized representation, in which each cell's features are mix-max normalized relative to its spatiotemporal k-nearest neighbors (k = 15). For both normalization schemes, we performed similar iterative training and prediction with XGBoost and recomputed confusion matrices and feature importances for individual features and feature categories (position, membrane shape, nucleus shape, movement, neighbor feature distance). Interestingly, both normalisation strategies yielded consistent results.

Importantly, even after applying both global and local normalisation, movement-related features contributed only minimally to cell fate determination, confirming that our conclusions are robust to the choice of normalisation approach.

These results are now presented in a new Extended View Figure (Figure EV5), incorporated into the main text, and the *Methods* section has been expanded to provide a detailed description of both normalisation procedures.

3. In the 'Comparisons to related approaches', I could not help thinking of an article which use backtracking in a similar manner to predict the fate of infected cells: https://www.biorxiv.org/content/10.1101/2024.10.28.620606v1

We thank the reviewer for highlighting this relevant work. We appreciate the reference to the preprint, which has since been published in *Nature Communications* and employs backtracking of live-imaging data to predict the fate of infected cells.

We have now included a citation to the published study in the "*Comparisons to Related Approaches*".

4. In the Materials and Methods section, the Membrane segmentation paragraph, I suggest replacing "using their 'cyto2' model" with "using the 'cyto2' model".

We thank the reviewer for this helpful suggestion. We have corrected the phrasing in the Materials and Methods section to "using the existing cyto2 model."

Reviewer #3

Tolonen and colleagues present in the manuscript "Single-cell morphodynamics predict cell fate decisions during mucociliary epithelial differentiation" a novel analysis pipeline to facilitate omics-scale morphometric analysis of the mucociliary epithelium of developing frog epidermis. They developed long-term live cell imaging of the explanted epithelium, nuclei and membrane segmentation approaches, a predictive algorithm to identify and track cell divisions as well as approaches to extract morphological and motility features of tracked cells over developmental time. Then they use computational approaches to comprehensively analyze morphodynamics of cells and cell groups within the datasets. Finally, they use some "classic" and some machine learning approaches to use morphodynamic features for cell fate prediction aided by end-point IF-based assignment of cell identities and backtracking of cells towards their origin during epithelium development. While the prediction accuracy varies across cell types in the epithelium and some cell types remain hard to distinguish from each other, the overall approach and pipeline are impressive and will be useful for a potentially large group of researchers moving forward. Interestingly, one key message of the presented work is that within complex tissues in an in vivo like state (such as epidermis explants used here), cell morphology and behavior seem to carry

limited predictive potential, likely due to aspects regarding cell packing as well as similarities of cell behaviors during early phases of development (where they rather behave like a collective than distinguishable and separated groups of fated cells). This is an important piece of information, particularly in comparison to more artificial in vitro and organoid work published where morphology and cell behaviors can be used in some cases to predict cell identities. Overall, this is impressive work, presented and illustrated well. The methods will be very useful for a large audience of readers and the overall conclusions provide important new insights regarding setup, use and interpretation of omics-style morphometric analyses.
There are some remaining aspects that will require the author's attention before publication, but I am optimistic that they will be able to address the following remarks during revision. These remarks are pointed towards the biological aspects and context of the work rather than the computational approach, since I am not a specialist regarding the latter.

We thank the reviewer for their very positive and encouraging evaluation of our work. We greatly appreciate their recognition of the methodological advances, the biological insights, and the potential impact of our morphodynamics analysis pipeline. We also thank the reviewer for their constructive remarks, which have helped us further improve the manuscript's biological context and clarity.

1. Developmental lineages, cell positions and behaviors have been described in the model system by the authors and other groups. However, little of that knowledge has been used to contextualize the morphometric analysis, and few studies are even cited and taken into consideration at all. Expanding on that front could improve this work and the interpretation of data in form of hypothesis testing.
Particularly, it is known that goblet cells are developing from the outer layer while basal and intercalating cells are generated from deep layer epithelial cells. This likely affects the predictability of cell fates, which is best for these goblet cells as well as basal cells that are born and remain in the deep layer. However, this aspect (where these cells are "born") is largely omitted. REF: e.g. PMID: 16728476, PMID: 28095645, PMID: 10518489

We thank the reviewer for this valuable comment and for highlighting the importance of integrating known developmental lineage relationships into our interpretation of the results. We have now expanded the *Introduction* to better contextualize our morphometric analysis in light of established lineage trajectories in the *Xenopus* mucociliary epidermis. We have also added the suggested references to support this discussion. We also describe in the *Results* section how the origin of goblet, basal, and intercalating cells from distinct epithelial layers likely contributes to their differing predictability in our fate analysis.

2. Furthermore, it has been described that MCCs engage in active migration-repulsion behavior in vivo to facilitate even spacing of MCCs across the epithelium, which clearly distinguishes

MCCs from other intercalating cell types. However, neither is the study cited, nor is this aspect tested or discussed although MCCs seem even spaced as shown in Fig. 4B. REF: PMID: 33756121

We thank the reviewer for highlighting the study by Chuyen et al. (PMID: 33756121), which describes a repulsion-based mechanism contributing to the even spacing of MCCs during epithelial development. We agree that this represents an important and well-established aspect of MCC biology, and we have now cited this work in the Introduction.

While our dataset shows progressive spatial separation of MCCs consistent with their dispersed organization, dissecting the underlying repulsion mechanism is beyond the scope of the present study. Our work focuses on large-scale morphodynamic profiling and fate prediction rather than on resolving fine-scale contact-dependent interactions. Moreover, the imaging configuration and temporal resolution used here do not allow direct quantification of repulsion dynamics at the level described by Chuyen et al. Importantly, our quantitative analysis further shows that neither global cell movement nor movement normalized to the local neighborhood contributes significantly to cell fate prediction, indicating that motion-related features play only a limited role in distinguishing MCCs from other intercalating lineages within the analyzed time window.

3. After intercalation, MCCs undergo extensive and active apical surface expansion, while in ICs and SSCs the apical surface size remains relatively large, and goblet cells derived from the outer layer show a large surface area from the very beginning. Again, this seems so obvious to analyze for this reviewer, that it is a surprise it has not been addressed in more detail. REF: e.g. PMID: 26766441, PMID: 24598162

We thank the reviewer for highlighting the importance of apical surface expansion in MCC differentiation and for pointing us to the relevant studies, which we now cite in the Introduction. We agree that apical domain expansion is a defining morphological feature of intercalating MCCs and represents a key aspect of their differentiation.

In our live-imaging dataset, however, accurate quantification of full apical surface dynamics was limited by the imaging configuration. Because imaging was performed on an inverted microscope through a dense, multilayered, pigment- and vesicle-rich tissue, consistent reconstruction of the complete apico-basal extent of intercalating cells, particularly at early stages, was not always feasible. As a result, volumetric measurements of apical expansion during radial intercalation are partially constrained in the live dataset.

To address this limitation, in line with our response to Reviewer 2, we performed complementary high-resolution imaging of fixed tissue in both apical and basal orientations, followed by computational alignment to reconstruct the full volumetric morphology. In this dataset, we quantified apical surface area and other 2D morphological descriptors across cell types. Consistent with previous reports, apical domain size and associated 2D shape measures loaded

strongly on the first principal component and contributed substantially to separation between MCCs and other MCE cell types in the principal component space. However, even when considered alone or combined with additional membrane and nuclear features, static apical morphology was insufficient to fully segregate cell identities in the 2D feature space.

These findings support our central conclusion: while apical expansion is a prominent and biologically meaningful feature of MCC differentiation, static morphological descriptors alone do not robustly resolve cell fate without incorporating temporal morphodynamic information. We have expanded the "*Limitations and Data Constraints*" section to clarify these points, included the results in a new Extended View Figure (Figure EV3) and an accompanying results section, and now explicitly reference the suggested studies.

4. The epidermis shows a remarkable degree of consistent cell type proportionality, where MCCs, ICs and SSCs are produced in approximately equal numbers. However, it seems like ICs and SSCs are relatively reduced in numbers as compared to MCCs in this system. Is this a problem of cell type identification or is this a result of the culture conditions? And how might this impact the analysis and interpretation overall? REF: e.g. PMID: 40086446, PMID: 30151772, PMID: 31553905

We thank the reviewer for this important point and for highlighting the relative proportions of cell types in our explant system. Indeed, as noted, ionocytes (ICs) and small secretory cells (SSCs) appear to be less frequent than multiciliated cells (MCCs). This difference most likely reflects culture-related biases inherent to the explant system, rather than misidentification of cell types.

Importantly, this compositional difference does not impact the performance of our predictive model. The model was trained using a class-balanced strategy that accounts for inherent differences in cell-type abundance, thereby preventing overrepresentation of the dominant population and ensuring robust prediction of less frequent cell types.

5. What happens in the analysis when cell divisions are not captured correctly? Are these cells/objects excluded? Same true for other analysis problems/artifacts (e.g. bad segmentation of membranes, unclear cell identities, etc.)? It is mentioned that only 33% of cells were successfully tracked, how does that affect analysis and what happens to the 66% of information lost?

We thank the reviewer for this important question regarding tracking and data completeness. For quantitative analyses showing cell type and for our predictive model evaluation, we used a manually curated ground-truth where cells were manually tracked for as long as they were visible in the time-lapse for the annotator. Specifically, the two major lineages, basal and goblet cells, were assigned final fates based on endpoint immunostaining at the last time point, but their automated tracks were used to generate the training datasets. All endpoint cell type annotated

MCC, IC, and SSC lineages were manually tracked to ensure correct lineage reconstruction before use in any cell-type–resolved analysis.
For these manually annotated tracks, cells for which an automated segmentation was missing were omitted from the downstream analyses. Regardless, the manual annotation ensures that only tracks with correct divisions and identity assignments were included in the downstream quantitative analyses. We have now clarified this point in the *Methods* section, *Nuclear tracking*.

While our quantitative analyses and performance estimates are based strictly on this high-confidence, manually curated ground-truth set, the trained classifier operates on single-cell feature vectors and could in principle be applied to less reliably tracked cells as well. We view such large-scale, fully automated fate prediction on all segmented cells as an important direction for future work, which will require further model refinement and improved accuracy, particularly for minority lineages such as ICs and SSCs. We have added a statement in the *Discussion* addressing potential biases introduced by incomplete tracking and clarified how these limitations were mitigated.

6. It is unclear to me which embryonic stages match the stages of the explant development. 22h (length of the movie) after st. 8/9 cutting explants seems a bit short for full development of the epidermis. Please clarify if embryos were used to stage match explants or how this was determined.

We thank the reviewer for this helpful comment. We have now clarified in the *Methods* section how developmental timing in explants was determined and how it was aligned with *in vivo* embryonic stages.

Explants were derived from stage 8/9 embryos according to the Nieuwkoop and Faber (NF) staging system and cultured at room temperature (RT). To ensure accurate temporal alignment, intact sibling embryos were cultured in parallel under identical conditions and morphologically staged at the beginning and end of the experiment. Explant development was matched to that of these sibling embryos using established morphological criteria, allowing us to correlate the 22-hour imaging period with the corresponding *in vivo* developmental stages. Under these conditions, epidermal differentiation proceeds comparably to intact embryos, reaching stages consistent with mature mucociliary epidermis formation within the recorded timeframe.

7. It is not fully clear to me if time-overlapping movies were taken to generate full developmental time coverage. Please clarify.

We thank the reviewer for this helpful comment. Each time-lapse recording is an individual, independent movie that captures the full developmental progression of an explant. The different recordings thus correspond to separate experiments rather than overlapping sequences, and

together they provide consistent coverage of mucociliary epithelial differentiation across multiple samples. The overview of the movies is provided in Table S1.

8. The authors mention that cells undergoing cell division maintain cell fates and both daughter cells have been assigned the same identity in the analysis. But this is followed by "data not shown" statement. Please explain and provide the data or rather remove from the manuscript.

We thank the reviewer for pointing this out. We agree that statements referring to “data not shown” are not appropriate. The sentence on cell division and fate maintenance has been removed from the manuscript, as it is not central to the study's main conclusions. The revised text avoids any unsupported claims.

9. Top page 3: The authors state that ECM stiffness regulates cell fates "independent" of genetic information, while I would argue that it regulates it in cooperation with genetic information and status of the cells.

We thank the reviewer for this important comment. We agree that the original wording was overly strong. We did not intend to imply that ECM stiffness acts independently of genetic information, but rather that it can modulate cell fate decisions in a manner that is not solely determined by genetic programs.

We have revised the text to clarify that ECM stiffness regulates cell fate in cooperation with genetic and intracellular regulatory states, thereby better reflecting the interplay between mechanical cues and gene expression programs.

10. The authors use MCE for mucociliary epithelium throughout the manuscript. I would suggest to be specific and rename to mucociliary epidermis, as there are additional mucociliary epithelia in the organism.

We thank the reviewer for this thoughtful suggestion. We agree that the developing *Xenopus* mucociliary tissue corresponds anatomically to the embryonic epidermis. However, we have chosen to retain the term “mucociliary epithelium (MCE)” because it is widely used in the field to describe this model system and emphasizes its functional and cellular similarities to other mucociliary epithelia, including the airway. To avoid potential ambiguity, we now clarify this explicitly in the *Introduction*.

11. Page 5, first paragraph of results: The authors state that explants were cut from "superficial ectoderm". This term is confusing and suggests that only an apical portion has been used. However, it is the animal located ectoderm that is removed for culture.

We thank the reviewer for pointing out this potential source of confusion. We agree that the term "superficial ectoderm" may be misleading in this context. What we refer to are animal cap explants derived from the animal pole ectoderm, which include the full thickness of the ectodermal layer rather than only an apical portion. We have revised the wording throughout the manuscript to use the more precise term "animal cap ectoderm" to avoid ambiguity.

12. The authors use the term ground truth a lot. Please specify in the given contexts what is specifically meant (from my reading I could only place cell type identities derived by IF as one such ground truth, but in other cases I wasn't sure what they meant).

We thank the reviewer for this important comment. We agree that the term "ground truth" was used too broadly and may have been ambiguous in different parts of the manuscript.

In the context of cell fate prediction, "ground truth" refers specifically to experimentally validated cell-type identities derived from endpoint immunofluorescence staining and subsequent manual annotation. These immunofluorescence-based cell-identity assignments serve as reference labels for training and evaluating classification models.

In other contexts (e.g., segmentation and tracking validation), "ground truth" refers to manually annotated reference datasets used to benchmark algorithm performance.

To avoid confusion, we have revised the relevant sections of the manuscript with this distinction in mind and clarified the wording accordingly.

2nd Apr 2026

Manuscript Number: MSB-2025-13344R
Title: Single-cell morphodynamics predict cell fate decisions during mucociliary epithelial differentiation

Dear Dr Sedzinski,

Thank you for the submission of your revised manuscript to Molecular Systems Biology. We have now received the enclosed reports from the referees that were asked to re-assess it. As you will see the reviewers are now globally supportive and I am pleased to inform you that we will be able to accept your manuscript pending the following final amendments and appropriate response to reviewers:

1) We require all corresponding authors to link their ORCID ID to their profile in our system. An email with instructions was sent on March 5th to Dr. Bianca Dumitrascu, but the profile has not yet been updated. Please ensure that the ORCID ID is now linked. In case additional help/instructions are needed, please ask Dr. Dumitrascu to contact us directly.
2) In the main manuscript file, please reduce keywords to max. 5.
3) Please format the Data availability section according to the example below.
"The datasets and computer code produced in this study are available in the following databases:
- Chip-Seq data: Gene Expression Omnibus GSE46748 (https://www.ncbi.nlm.nih.gov/geo/query/acc.cgi?acc=GSE46748)
- Modeling computer scripts: GitHub (https://github.com/SysBioChalmers/GECKO/releases/tag/v1.0)
- [data type]: [full name of the resource] [accession number/identifier] ([doi or URL or identifiers.org/DATABASE:ACCESSION])"
- Please remove the sections "Lead contact" and "Materials availability"; in addition, please only include the heading "Data availability" and not "Data and code availability" (despite the fact that available code is also listed in this section).
4) Please remove the sections "Lead contact" and "Materials availability"; in addition, please only include the heading "Data availability" and not "Data and code availability" (despite the fact that available code is also listed in this section).
5) Please include a README file on trackomics Github with practical use instructions for potential future users of your code.
6) Please remove the author contributions from the manuscript and specify author contributions in our submission system. CRediT has replaced the traditional author contributions section because it offers a systematic machine-readable author contributions format that allows for more effective research assessment. You are encouraged to use the free text boxes beneath each contributing author's name to add specific details on the author's contribution. More information is available in our guide to authors: https://link.springer.com/journal/44321/submission-guidelines#authorshipguidelines
7) In the Methods, please take care of the following:
- Please ensure that a statement on whether or not blinding was done is included in the Methods even if no blinding was done. Please also be sure to update the Author Checklist with this information and where it can be found in the manuscript.
- Please move the AI declaration so that it is placed in the Methods section.
8) Please place individual sections of the manuscript in the following order: Title page - Abstract & Keywords - Introduction - Results - Discussion - Methods - Data Availability - Acknowledgements - Disclosure and Competing Interests Statement - References - Figure Legends - Main Tables with legends - Expanded View Figure Legends.
9) For the figures and figure legends, please take care of the following:
- Please make sure to update the callouts of all figures in the main manuscript text. Please correct the callout for Appendix Figure S6 (currently it is Appendix Figure 6). In addition, a callout is missing for Figure 2E.
- Please note that the box plots need to be defined in terms of minima, maxima, centre, bounds of box and whiskers, and percentile in the legend of figure 3G
- Please note that information related to n is missing in the legends of figures 3G, EV4 A
- Please note that the error bars are not defined in the legend of figure EV4 A.
- Please note that the scale bar needs to be defined for figure 1C
- Please note that scale bar and its definition are missing for figure 4C
10) Please zip the movie files with their legends that have been provided in a separate text file (and remove these legends from the manuscript). There should be one movie per folder with the nomenclature Movie EV1, etc.
11) In the Appendix file please remove the author list and affiliations from the cover page, just include "Appendix for + manuscript title". Please also update the nomenclature of the tables to be labeled as Appendix Table S1, S2 etc. instead of Table S1, S2 etc (callouts in the manuscript are fine though).
12) Synopsis:
- Synopsis image: Please provide the synopsis image in JPEG, TIFF, or PNG format. In addition, a recent change to our Production pipeline requires that synopses images be provided as a high-resolution image with the exact dimensions 550 pixels wide x 300 pixels high.
- Please remove the synopsis text from the main manuscript file and provide it as a a separate word document.
- Please check your synopsis text and image before submission with your revised manuscript. Please be aware that in the proof stage minor corrections only are allowed (e.g., typos).
13) As part of the EMBO Publications transparent editorial process initiative (see our policy here: https://www.embopress.org/transparent-process#Review_Process), Molecular Systems Biology will publish online a Peer Review File (PRF) to accompany accepted manuscripts. This file will be published in conjunction with your paper and will include

the anonymous referee reports, your point-by-point response and all pertinent correspondence relating to the manuscript. Let us know whether you agree with the publication of the PRF and as here, if you want to remove or not any figures from it prior to publication. Please note that the Authors checklist will be published at the end of the PRF.
14) After your paper is published, we may promote it on social media. If you have any handles or hashtags for Bluesky you would like included, please let us know.
15) Please provide a point-by-point letter INCLUDING my comments as well as the reviewer's reports and your detailed responses (as Word file).

I look forward to reading a new revised version of your manuscript as soon as possible.

Yours sincerely,

Poonam Bheda, PhD
Senior Scientific Editor
Molecular Systems Biology

Reviewer #1:

The authors have appropriately addressed any concerns that I had and I am in full support of publication for this beautiful paper.

Reviewer #2:

I thank the author for the work they put in this revision. All my points are addressed in a very satisfactory manner. In particular I am thankful for the new discussion item in 'Limitations and Data constraints`, backed up by the Figure EV3. It helps frame the predictive power of the computational approach within a scope conferred by the accuracy of imaging and segmentation.

I recommend accepting this manuscript as it is now.

Reviewer #3:

In this revised manuscript the authors have put significant effort to address the reviewer's points. Overall, this has improved the presented work. The technical advances for live cell imaging analysis of cell in a densly packed tissue are really great in this work and it should be published. There are still some points for which clarification or adjustment of wording would be beneficial in my opinion.

1) Given that overall dynamic morphometrics seem to be limited regarding the prediction of cell fates, I feel that the title is an over claim, as de facto the moprhodynamics can not do this reliably. The best predictor still remains the place from which cells emerge (outer or deep layer) after the additional analysis. It would be good if this was represented in the title.

2) The same is true for the text. E.g. "While single features showed high noise and low separability of ground-truth cell types, supervised machine learning revealed that integrating time-resolved features robustly predicts final cell fate. Gradient-boosted trees and multinomial logistic regression achieved moderate but consistent accuracy, especially for abundant epithelial lineages."
The analysis does not support the claim that it robustly predicts cell fate - in the next sentence they write "moderate accuracy". While the wording is of course debatable, perhaps it would be good to tone down a bit.

3) And the same is true for cell division detection and tracking. They claim "providing robust coverage for downstream analyses of morphodynamics". However, in Figure S3 C-I, they show that >50% of cases are false positives or false negatives. The correct cases are always a minority. While I understand the challenge, the wording is again giving an impression of robustness that I don't see supported by their analysis.

4) Regarding the end point comparison, the authors answer to reviewer 1 question:
"While I do not doubt the model at all, it would be nice to have an end point comparison of cell shapes in explants versus embryos.

We thank the reviewer for this helpful and constructive suggestion. In response, we have now included an endpoint comparison of cell and nuclear morphology between explants and embryos at different developmental stages, assessed from both the apical

and basal sides of the tissue (new Appendix Figure S1). This analysis confirms that the explant system faithfully recapitulates the overall in vivo tissue architecture and cellular morphology."

However, the images in S1 do not really address this, as there are clear differences regarding apical morphology (less intercalating cells, particularly ICs and SSCs not really apparent, while clearly visible in the embryo images). Without a better description/quantification, the statement that they faithfully recapitulate cellular and tissue morphology is a bit misleading. The issue of differences in cell types detected (less ICs and SSCs in their analysis) is also not fully described and instead they state that MCCs are a more abundant cell type. I do not find this sufficiently transparent.

5) Overall, it is still puzzling to me that neither local mobility, nor final apical surface size seem to distinguish cell types in the analysis. These are clearly different between cell types. For the mobility aspect, I would have expected an approach where the drift is canceled out, but from what I understand a different approach was taken. In my opinion it should be transparently stated that these differences exist between other studies and this "high-throughput" approach and delineate what the advantages/use cases are for the different methods.
Similar for the apical surface size/morphology. Particularly as the biggest confusion seem to be between ICs and SSCs (Figure 5B/C), both cell types have in common that they have small apical surfaces and conical shape in vivo. So final cell shape seems to have quite an influence. This seems to contradict the author's statements. Looking at the PCA EV4 D, the different cell types do actually separate (not worse than many RNA-seq PCAs or plots from flow cytometry). This would be even more clear when "abundant" cell types basal and goblet were removed from the plot. This would be justified as they can be tracked and predicted better than the intercalating cell types and do not change positioning between outer and inner layer. Why not taking such a 2-step approach? The authors might have good reasons, but this should be at least discussed.

6) In Figure 4B, it would be good to label which markers were used for IF and in the IF images (upper row) the location of the identified cell types should be indicted for clarity.

7) "To ensure correct representation of cell lineages, we manually curated trajectories for MCCs (n = 156), ICs (n = 45), and SSCs (n = 11), which are less abundant and more morphologically variable cell types."
This statement is only true for their analysis, but not in the embryo. But it reads like a fact, which I find problematic.

8) In Figure 5E: What does it mean that removing Z-position and memebrane-nucleus offset improves classification of SSCs? These are the only two cases where removing features really improves the analysis but it is not explained or discussed.

9) Please explain the rational for this approach: "The plugin optionally applies the MARI (Mitosis Angular Region of Interest) principle, which filters division events to retain only those in which daughter cells emerge perpendicular to the mother cell's nuclear major axis (see Materials and methods)."

2nd Apr 2026

Manuscript Number: MSB-2025-13344R
Title: Single-cell morphodynamics predict cell fate decisions during mucociliary epithelial differentiation

Dear Dr Sedzinski,

Thank you for the submission of your revised manuscript to Molecular Systems Biology. We have now received the enclosed reports from the referees that were asked to re-assess it. As you will see the reviewers are now globally supportive and I am pleased to inform you that we will be able to accept your manuscript pending the following final amendments and appropriate response to reviewers.

We thank the editor and reviewers for their thoughtful and constructive comments, which have helped us further improve the manuscript. We are glad that the overall opinions support the publication of the manuscript. We provide our detailed responses to all editorial and reviewer comments below.

1) We require all corresponding authors to link their ORCID ID to their profile in our system. An email with instructions was sent on March 5th to Dr. Bianca Dumitrascu, but the profile has not yet been updated. Please ensure that the ORCID ID is now linked. In case additional help/instructions are needed, please ask Dr. Dumitrascu to contact us directly.

The author confirmed that the issue has been resolved.

2) In the main manuscript file, please reduce keywords to max. 5.

We have reduced the number of keywords to 5.

3) Please format the Data availability section according to the example below.
"The datasets and computer code produced in this study are available in the following databases:
- Chip-Seq data: Gene Expression Omnibus GSE46748 (https://www.ncbi.nlm.nih.gov/geo/query/acc.cgi?acc=GSE46748)
- Modeling computer scripts: GitHub (https://github.com/SysBioChalmers/GECKO/releases/tag/v1.0)
- [data type]: [full name of the resource] [accession number/identifier] ([doi or URL or identifiers.org/DATABASE:ACCESSION])"
- Please remove the sections "Lead contact" and "Materials availability"; in addition, please only include the heading "Data availability" and not "Data and code availability" (despite the fact that available code is also listed in this section).

We have resolved these issues.

4) Please remove the sections "Lead contact" and "Materials availability"; in addition, please only include the heading "Data availability" and not "Data and code availability" (despite the fact that available code is also listed in this section).

We have resolved these issues.

5) Please include a README file on trackomics Github with practical use instructions for potential future users of your code.

We have included the README file on GitHub.

6) Please remove the author contributions from the manuscript and specify author contributions in our submission system. CRediT has replaced the traditional author contributions section because it offers a systematic machine-readable author contributions format that allows for more effective research assessment. You are encouraged to use the free text boxes beneath each contributing author's name to add specific details on the author's contribution. More information is available in our guide to authors: https://link.springer.com/journal/44321/submission-guidelines#authorshipguidelines

We have removed the author contributions from the manuscript.

7) In the Methods, please take care of the following:
- Please ensure that a statement on whether or not blinding was done is included in the Methods even if no blinding was done. Please also be sure to update the Author Checklist with this information and where it can be found in the manuscript.

We have included information on blinding in the Method section and have updated the Author Checklist.

- Please move the AI declaration so that it is placed in the Methods section.

We have moved the AI decoration to the Method section.

8) Please place individual sections of the manuscript in the following order: Title page - Abstract & Keywords - Introduction - Results - Discussion - Methods - Data Availability - Acknowledgements - Disclosure and Competing Interests Statement - References - Figure Legends - Main Tables with legends - Expanded View Figure Legends.

We have structured the manuscript according to the provided instructions.

9) For the figures and figure legends, please take care of the following:
- Please make sure to update the callouts of all figures in the main manuscript text. Please correct the callout for Appendix Figure S6 (currently it is Appendix Figure 6). In addition, a callout is missing for Figure 2E.
- Please note that the box plots need to be defined in terms of minima, maxima, centre, bounds of box and whiskers, and percentile in the legend of figure 3G
- Please note that information related to n is missing in the legends of figures 3G, EV4 A
- Please note that the error bars are not defined in the legend of figure EV4 A.
- Please note that the scale bar needs to be defined for figure 1C
- Please note that scale bar and its definition are missing for figure 4C

We have resolved all these issues.

10) Please zip the movie files with their legends that have been provided in a separate text file (and remove these legends from the manuscript). There should be one movie per folder with the nomenclature Movie EV1, etc.

We have prepared the movie field according to the instructions.

11) In the Appendix file please remove the author list and affiliations from the cover page, just include "Appendix for + manuscript title". Please also update the nomenclature of the tables to be labeled as Appendix Table S1, S2 etc. instead of Table S1, S2 etc (callouts in the manuscript are fine though).

We have resolved these issues.

12) Synopsis:
- Synopsis image: Please provide the synopsis image in JPEG, TIFF, or PNG format. In addition, a recent change to our Production pipeline requires that synopses images be provided as a high-resolution image with the exact dimensions 550 pixels wide x 300 pixels high.
- Please remove the synopsis text from the main manuscript file and provide it as a a separate word document.
- Please check your synopsis text and image before submission with your revised manuscript. Please be aware that in the proof stage minor corrections only are allowed (e.g., typos).

We have resolved these issues.

13) As part of the EMBO Publications transparent editorial process initiative (see our policy here: https://www.embopress.org/transparent-process#Review_Process), Molecular Systems Biology will publish online a Peer Review File (PRF) to accompany accepted manuscripts. This file will be published in conjunction with your paper and will include the anonymous referee reports, your point-by-point response and all pertinent correspondence relating to the manuscript. Let us know whether you agree with the publication of the PRF and as here, if you want to remove or not any figures from it prior to publication. Please note that the Authors checklist will be published at the end of the PRF.

We agree to publish the PRF file and keep all its content.

14) After your paper is published, we may promote it on social media. If you have any handles or hashtags for Bluesky you would like included, please let us know.

Thank you for this promotion. Please use #phenomics, #morphodynamics, #cellfate, #xenopus and handles: @kuba-sedzinski.bsky.social

15) Please provide a point-by-point letter INCLUDING my comments as well as the reviewer's reports and your detailed responses (as Word file).

We have provided answers to all the editor's and reviewers' questions.

Reviewer #1:

The authors have appropriately addressed any concerns that I had and I am in full support of publication for this beautiful paper.

We sincerely thank the reviewer for their positive and encouraging assessment of our work, and for their support of its publication. We greatly appreciate the time and effort invested in evaluating our manuscript.

Reviewer #2:

I thank the author for the work they put in this revision. All my points are addressed in a very satisfactory manner. In particular I am thankful for the new discussion item in 'Limitations and Data constraints`, backed up by the Figure EV3. It helps frame the predictive power of the computational approach within a scope conferred by the accuracy of imaging and segmentation.

I recommend accepting this manuscript as it is now.

We thank the reviewer for their careful reassessment and supportive recommendation. We are pleased that the added discussion in "Limitations and Data constraints" and the supporting analysis (Figure EV3) addressed the reviewer's concerns.

Reviewer #3:

In this revised manuscript the authors have put significant effort to address the reviewer's points. Overall, this has improved the presented work. The technical advances for live cell imaging analysis of cell in a densly packed tissue are really great in this work and it should be published. There are still some points for which clarification or adjustment of wording would be beneficial in my opinion.

1) Given that overall dynamic morphometrics seem to be limited regarding the prediction of cell fates, I feel that the title is an over claim, as de facto the moprhodynamics can not do this reliably. The best predictor still remains the place from which cells emerge (outer or deep layer) after the additional analysis. It would be good if this was represented in the title.

2) The same is true for the text. E.g. "While single features showed high noise and low separability of ground-truth cell types, supervised machine learning revealed that integrating time-resolved features robustly predicts final cell fate. Gradient-boosted trees and multinomial logistic regression achieved moderate but consistent accuracy, especially for abundant epithelial lineages."

The analysis does not support the claim that it robustly predicts cell fate - in the next sentence they write "moderate accuracy". While the wording is of course debatable, perhaps it would be good to tone down a bit.

We thank the reviewer for this important point and agree that the title and abstract should not encourage overinterpretation. In the original abstract, the phrase "robustly predicts" could be interpreted as implying uniformly strong predictive performance, which is not supported by our data, particularly for minority lineages. We have therefore removed the term "robustly" from the manuscript to avoid overstating the predictive performance and revised the abstract to state that integrating time-resolved features improves prediction, with models achieving moderate but consistent accuracy, particularly for abundant epithelial lineages.

We respectfully prefer to retain the current title ("Single-cell morphodynamics predict cell fate decisions…") for two reasons. First, the use of "predict" is supported by reproducible held-out performance above baseline across randomized train–test splits using supervised models. Second, the reviewer's key point, positional context, is already an explicit component of our definition of morphodynamics, which includes time-resolved features of cell and nuclear shape, movement, and 3D position (including apico-basal location).

We hope these revisions address the reviewer's concern while preserving a concise title that reflects the central contribution of the work.

3) And the same is true for cell division detection and tracking. They claim "providing robust coverage for downstream analyses of morphodynamics". However, in Figure S3 C-I, they show that >50% of cases are false positives or false negatives. The correct cases are always a minority. While I understand the challenge, the wording is again giving an impression of robustness that I don't see supported by their analysis.

We thank the reviewer for highlighting this wording issue. We agree that the term "robust" may imply a level of accuracy in lineage reconstruction and division detection that is not supported by our analysis. Our primary objective, however, is to obtain sufficiently long and continuous single-cell trajectories for morphodynamic feature extraction, rather than to achieve complete or highly accurate lineage reconstruction. As shown in Appendix Figure S3, division detection remains imperfect, and we now state this more explicitly.

Manuscript change (specific wording edits):
We replaced "providing robust coverage for downstream analyses of morphodynamics" with:
"providing sufficient coverage for downstream morphodynamic analyses, while lineage reconstruction remains imperfect."

4) Regarding the end point comparison, the authors answer to reviewer 1 question:

"While I do not doubt the model at all, it would be nice to have an end point comparison of cell shapes in explants versus embryos.

We thank the reviewer for this helpful and constructive suggestion. In response, we have now included an endpoint comparison of cell and nuclear morphology between explants and embryos at different developmental stages, assessed from both the apical and basal sides of the tissue (new Appendix Figure S1). This analysis confirms that the explant system faithfully recapitulates the overall in vivo tissue architecture and cellular morphology."

However, the images in S1 do not really address this, as there are clear differences regarding apical morphology (less intercalating cells, particularly ICs and SSCs not really apparent, while clearly visible in the embryo images). Without a better description/quantification, the statement that they faithfully recapitulate cellular and tissue morphology is a bit misleading. The issue of differences in cell types detected (less ICs and SSCs in their analysis) is also not fully described and instead they state that MCCs are a more abundant cell type. I do not find this sufficiently transparent.

We thank the reviewer for this thoughtful comment and for raising important points regarding the interpretation of the explant data. We note that in the manuscript, we do not state that explants "faithfully recapitulate" in vivo tissue organization; rather, we state that the system "recapitulates the cell morphologies of the embryonic tissue," referring specifically to qualitative morphological features (Figure 1C, Appendix Figure S1).

We agree that the current comparison is primarily qualitative and does not fully capture differences in cell type composition, including the apparent underrepresentation of ICs and SSCs in explants. Our intention in this analysis was to address the reviewer's request for a comparison of cell and nuclear morphology between explants and embryos, rather than to provide a comprehensive assessment of cell type composition. A more quantitative and systematic comparison of cell populations and tissue organization would be valuable; however, this is beyond the scope of the current study.

We also acknowledge that our statement regarding the higher abundance of MCCs could be interpreted as a direct comparison to embryos. This was not our intention, as it reflects the relative representation in our analyzed dataset rather than a quantitative comparison of cell-type composition between conditions. We have revised the manuscript to clarify this point and avoid potential misinterpretation.

To further improve transparency, we have refined the wording describing the explant system to more precisely reflect its scope and have added a statement in the limitations section explicitly acknowledging that explants may not fully reproduce the cell-type composition and tissue architecture observed in embryos.

We hope these clarifications address the reviewer's concerns while maintaining the intended scope of the analysis.

5) Overall, it is still puzzling to me that neither local mobility, nor final apical surface size seem to distinguish cell types in the analysis. These are clearly different between cell types. For the mobility aspect,

I would have expected an approach where the drift is canceled out, but from what I understand a different approach was taken. In my opinion it should be transparently stated that these differences exist between other studies and this "high-throughput" approach and delineate what the advantages/use cases are for the different methods.
Similar for the apical surface size/morphology. Particularly as the biggest confusion seem to be between ICs and SSCs (Figure 5B/C), both cell types have in common that they have small apical surfaces and conical shape in vivo. So final cell shape seems to have quite an influence. This seems to contradict the author's statements. Looking at the PCA EV4 D, the different cell types do actually separate (not worse than many RNA-seq PCAs or plots from flow cytometry). This would be even more clear when "abundant" cell types basal and goblet were removed from the plot. This would be justified as they can be tracked and predicted better than the intercalating cell types and do not change positioning between outer and inner layer. Why not taking such a 2-step approach? The authors might have good reasons, but this should be at least discussed.

We thank the reviewer for this insightful and constructive comment. We agree that both local mobility and apical morphology differ between cell types and can be informative descriptors, and that different analytical approaches may emphasize these features to varying degrees.

To directly address the reviewer's concern regarding global drift, we implemented an alternative preprocessing strategy in which each cell's features were normalized relative to its local neighborhood (k-nearest spatial neighbors at the same timepoint). This local normalization effectively removes tissue-wide offsets and emphasizes local differences in both movement and morphological features. We compared this approach to our original global normalization using identical training and evaluation pipelines. Across both strategies, we obtained highly consistent results, and movement-related features remained minimal contributors to cell fate prediction. This demonstrates that the limited contribution of mobility is not due to uncorrected drift or preprocessing choices (new Figure EV5).

Regarding apical surface size and the suggestion of a two-step classification approach, we agree that endpoint morphology varies among certain cell types, particularly between closely related intercalating populations such as ICs and SSCs. However, implementing a hierarchical or two-step strategy would not fundamentally change our conclusions. Our current framework is already lineage-aware, accounts for differences in cell-type abundance through balanced training and evaluation, and is designed to operate across all cell types without prior stratification.

We further note that PCA representations (Appendix Figure EV4D) correspond to a two-dimensional projection of inherently high-dimensional feature space. While partial separation is visible in this reduced representation, it does not necessarily translate into robust classification performance in a supervised and cross-validated setting.

More broadly, we agree with the reviewer that it is important to distinguish our high-throughput approach from more targeted or high-resolution analyses used in other studies. Approaches that focus on specific features or subsets of cells can provide detailed insights into particular aspects of cell behavior, such as local motility or apical morphology. In contrast, our framework is designed to capture large numbers of cells over time, enabling the extraction of continuous morphodynamic trajectories and unbiased prediction

of cell fate across heterogeneous tissues. These approaches are therefore complementary rather than contradictory, with distinct advantages depending on the biological question.

We have revised the manuscript to clarify these points and added a corresponding discussion outlining the scope, limitations, and use cases of our approach.

6) In Figure 4B, it would be good to label which markers were used for IF and in the IF images (upper row) the location of the identified cell types should be indicted for clarity.

We agree and thank the reviewer for the suggestion. We have updated Figure 4B and its legend to explicitly explain the immunostaining markers and indicate the location of the example annotated cell(s) (arrowheads), improving interpretability.

7) "To ensure correct representation of cell lineages, we manually curated trajectories for MCCs (n = 156), ICs (n = 45), and SSCs (n = 11), which are less abundant and more morphologically variable cell types." This statement is only true for their analysis, but not in the embryo. But it reads like a fact, which I find problematic.

We thank the reviewer for pointing out this ambiguity. We agree that the phrasing could be interpreted as a general statement about cell type abundance and variability in the embryo, whereas our intention was to describe the relative representation and characteristics of cell types within our analyzed dataset.

We have therefore revised the wording to clarify that MCCs, ICs, and SSCs are less abundant and more morphologically variable *in our dataset*, in contrast to basal and goblet cells, which are more abundant and morphologically homogeneous under our imaging and analysis conditions.

We hope this clarification resolves the concern.

8) In Figure 5E: What does it mean that removing Z-position and memebrane-nucleus offset improves classification of SSCs? These are the only two cases where removing features really improves the analysis but it is not explained or discussed.

We thank the reviewer for their keen attention to the biological and technical details of our classification analysis. We share the reviewer's interest in identifying which features most accurately define the specific lineages of the MCE and offer the following clarifications:

While the leave-one-feature-out (LOFO) analysis suggests a marginal improvement in SSC prediction upon removing the Z-position or membrane-nucleus offset, and some other features as shown in Figure EV4D, the impact is less than one percentage point. Given the randomized nature of the train-test splits when assessing model performance, such small differences are subject to fluctuation between positive and negative effects across different runs.

The SSC and IC lineages represent the "minority classes" in our dataset (Figure EV4A). Because of their relatively low sample sizes, the classification results for these cells are less stable than those for the more abundant basal or goblet cells. Consequently, we refrain from drawing large-scale biological conclusions from these minor increases in accuracy, as they likely represent "noise" in the model's optimization for rare events.

As shown in the confusion matrices with these features removed from the XGBoost model (see **Fig. 1**), removing the Z-position results in a trade-off: while it slightly decreases confusion between SSCs and goblet cells, it simultaneously increases confusion between SSCs and basal cells. This suggests that the feature is biologically relevant to SSCs, and its removal shifts the classifier's bias in a way that slightly favors the SSC F1-score.

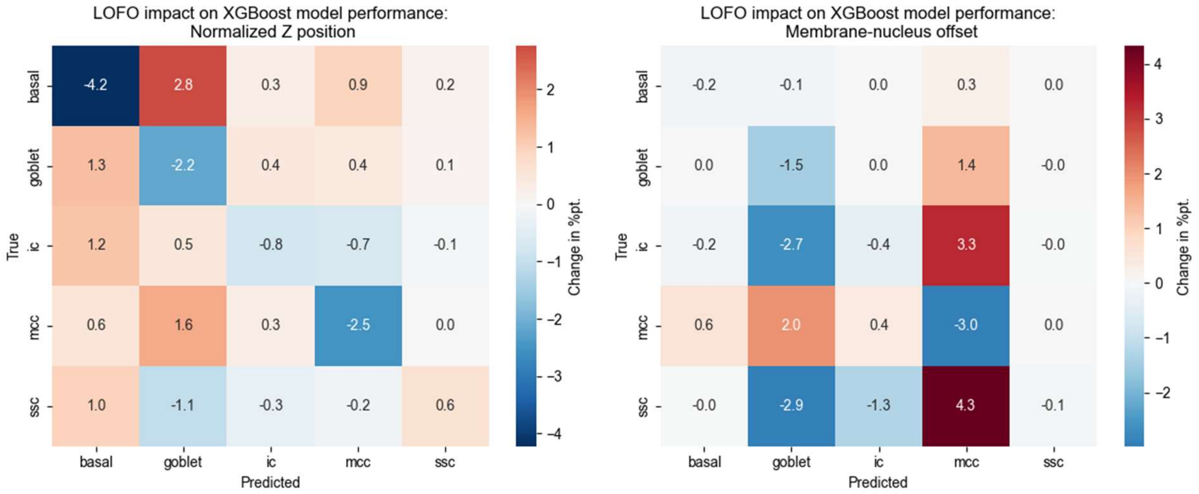

Fig 1. XGBoost confusion matrix upon removal of normalized Z position and membrane-nucleus offset features.

We acknowledge that the SSC class remains "finicky" due to these data constraints. In some iterations, removing the membrane-nucleus offset does not result in any improvement (Fig. 1). Interestingly, it increases confusion between SSCs and MCCs. However, this might again be iteration-specific. We plan to address this in future work by significantly increasing the training data for these rarer lineages to stabilize the feature-importance profiles.

To address this, we have added the following sentence to the **Results** section:

> "Simultaneously, while the removal of certain features, such as Z-position or membrane-nucleus offset, appeared to marginally improve classification accuracy for the SSC lineage (Figure 5F), these changes were within a single percentage point and likely reflect high prediction variance in low-sample-size populations rather than robust biological trends."

9) Please explain the rational for this approach: "The plugin optionally applies the MARI (Mitosis Angular Region of Interest) principle, which filters division events to retain only those in which daughter cells emerge perpendicular to the mother cell's nuclear major axis (see Materials and methods)."

We thank the reviewer for this request for clarification. The rationale for applying the MARI (Mitosis Angular Region of Interest) principle is to improve the reliability of automated detection of cell divisions and lineage assignment in densely packed tissues.

In our imaging conditions, division events can be difficult to resolve due to cell crowding, limited axial resolution, and segmentation uncertainties. As a result, incorrect assignment of daughter cells to a mother cell can occur, particularly when multiple neighboring cells are in close proximity. The MARI criterion provides a geometrical constraint based on the expected orientation of daughter cell emergence relative to the mother cell's nuclear major axis, allowing us to filter out ambiguous or likely incorrect division events.

Importantly, this filtering step is not intended to enforce a biological assumption about division orientation, but rather to increase the confidence of lineage reconstruction by excluding geometrically inconsistent or uncertain events.

16th Apr 2026
Manuscript number: MSB-2025-13344RR
Title: Single-cell morphodynamics predict cell fate decisions during mucociliary epithelial differentiation

Dear Dr Sedzinski,

Congratulations on an excellent manuscript, I am pleased to inform you that your manuscript has been accepted for publication in Molecular Systems Biology. Thank you for your comprehensive response to referee concerns and our editorial formatting requests. It has been a pleasure to work with you to get this to the acceptance stage.

Your manuscript will be processed for publication by EMBO Press. It will be copy edited and you will receive page proofs prior to publication. Please note that you will be contacted by Springer Nature Author Services to complete licensing and payment information.

You may qualify for financial assistance for your publication charges - either via a Springer Nature fully open access agreement or an EMBO initiative. Check your eligibility: https://link.springer.com/journal/44320/how-to-publish-with-us

Should you be planning a Press Release on your article, please get in contact with embo_production@springernature.com as early as possible in order to coordinate publication and release dates.

If you have any questions, please do not hesitate to contact the Editorial Office. Thank you for your contribution to Molecular Systems Biology.

Yours sincerely,

Sincerely,

Poonam Bheda, PhD
Scientific Editor
Molecular Systems Biology

>>> Please note that it is Molecular Systems Biology policy for the transcript of the editorial process (containing referee reports and your response letter) to be published as an online supplement to each paper. If you do NOT want this, you will need to inform the Editorial Office via email immediately. More information is available here: https://link.springer.com/partners/embo-press/editorial-policies#Peer%20review

EMBO Press Author Checklist

Corresponding Author Name: Jakub Sedzinski, Bianca Dumitrascu	
Journal Submitted to: MSB	
Manuscript Number: MSB-2025-13344	

USEFUL LINKS FOR COMPLETING THIS FORM
The EMBO Journal - Author Guidelines
EMBO Reports - Author Guidelines
Molecular Systems Biology - Author Guidelines
EMBO Molecular Medicine - Author Guidelines

Reporting Checklist for Life Science Articles (updated January
This checklist is adapted from Materials Design Analysis Reporting (MDAR) Checklist for Authors. MDAR establishes a minimum set of requirements in transparent reporting in the life sciences (see Statement of Task: 10.31222/osf.io/9sm4x). Please follow the journal's guidelines in preparing your manuscript.
Please note that a copy of this checklist will be published alongside your article.

Abridged guidelines for figures
1. Data
The data shown in figures should satisfy the following conditions:
- the data were obtained and processed according to the field's best practice and are presented to reflect the results of the experiments in an accurate and unbiased manner.
- ideally, figure panels should include only measurements that are directly comparable to each other and obtained with the same assay.
- plots include clearly labeled error bars for independent experiments and sample sizes. Unless justified, error bars should not be shown for technical
- if n<5, the individual data points from each experiment should be plotted. Any statistical test employed should be justified.
- Source Data should be included to report the data underlying figures according to the guidelines set out in the authorship guidelines on Data

2. Captions
Each figure caption should contain the following information, for each panel where they are relevant:
- a specification of the experimental system investigated (eg cell line, species name).
- the assay(s) and method(s) used to carry out the reported observations and measurements.
- an explicit mention of the biological and chemical entity(ies) that are being measured.
- an explicit mention of the biological and chemical entity(ies) that are altered/varied/perturbed in a controlled manner.
- the exact sample size (n) for each experimental group/condition, given as a number, not a range;
- a description of the sample collection allowing the reader to understand whether the samples represent technical or biological replicates (including how many animals, litters, cultures, etc.).
- a statement of how many times the experiment shown was independently replicated in the laboratory.
- definitions of statistical methods and measures:
  - common tests, such as t-test (please specify whether paired vs. unpaired), simple χ2 tests, Wilcoxon and Mann-Whitney tests, can be unambiguously identified by name only, but more complex techniques should be described in the methods section;
  - are tests one-sided or two-sided?
  - are there adjustments for multiple comparisons?
  - exact statistical test results, e.g., P values = x but not P values < x;
  - definition of 'center values' as median or average;
  - definition of error bars as s.d. or s.e.m.

Please complete ALL of the questions below.
Select "Not Applicable" only when the requested information is not relevant for your study.

Materials

Newly Created Materials	Information included in the manuscript?	In which section is the information available? (Reagents and Tools Table, Materials and Methods, Figures, Data Availability Section)
New materials and reagents need to be available; do any restrictions apply?	Not Applicable	

Antibodies	Information included in the manuscript?	In which section is the information available? (Reagents and Tools Table, Materials and Methods, Figures, Data Availability Section)
For **antibodies** provide the following information:<br>- Commercial antibodies: RRID (if possible) or supplier name, catalogue number and or/clone number<br>- Non-commercial: RRID or citation	Yes	Material and Methods; Reagents and Tools Table

DNA and RNA sequences	Information included in the manuscript?	In which section is the information available? (Reagents and Tools Table, Materials and Methods, Figures, Data Availability Section)
**Short novel DNA or RNA including primers, probes**: provide the sequences.	Not Applicable	

Cell materials	Information included in the manuscript?	In which section is the information available? (Reagents and Tools Table, Materials and Methods, Figures, Data Availability Section)
**Cell lines:** Provide species information, strain. Provide accession number in repository **OR** supplier name, catalog number, clone number, and/**OR** RRID.	Not Applicable	
**Primary cultures:** Provide species, strain, sex of origin, genetic modification status.	Not Applicable	
Report if the cell lines were recently **authenticated** (e.g., by STR profiling) and tested for mycoplasma contamination.	Not Applicable	

Experimental animals	Information included in the manuscript?	In which section is the information available? (Reagents and Tools Table, Materials and Methods, Figures, Data Availability Section)
**Laboratory animals or Model organisms:** Provide species, strain, sex, age, genetic modification status. Provide accession number in repository **OR** supplier name, catalog number, clone number, **OR** RRID.	Yes	Material and Methods
**Animal observed in or captured from the field:** Provide species, sex, and age where possible.	Not Applicable	
Please detail **housing and husbandry conditions**.	Yes	Material and Methods

Plants and microbes	Information included in the manuscript?	In which section is the information available? (Reagents and Tools Table, Materials and Methods, Figures, Data Availability Section)
**Plants:** provide species and strain, ecotype and cultivar where relevant, unique accession number if available, and source (including location for collected wild specimens).	Not Applicable	
**Microbes:** provide species and strain, unique accession number if available, and source.	Not Applicable	

Human research participants	Information included in the manuscript?	In which section is the information available? (Reagents and Tools Table, Materials and Methods, Figures, Data Availability Section)
If collected and within the bounds of privacy constraints report on age, sex and gender or ethnicity for all study participants.	Not Applicable	

Core facilities	Information included in the manuscript?	In which section is the information available? (Reagents and Tools Table, Materials and Methods, Figures, Data Availability Section)
If your work benefited from core facilities, was their service mentioned in the acknowledgments section?	Yes	Acknowledgements

Design

Study protocol	Information included in the manuscript?	In which section is the information available? (Reagents and Tools Table, Materials and Methods, Figures, Data Availability Section)
If study protocol has been **pre-registered, provide DOI in the manuscript**. For clinical trials, provide the trial registration number **OR** cite DOI.	Not Applicable	
Report the **clinical trial registration number** (at ClinicalTrials.gov or equivalent), where applicable.	Not Applicable	

Laboratory protocol	Information included in the manuscript?	In which section is the information available? (Reagents and Tools Table, Materials and Methods, Figures, Data Availability Section)
Provide DOI OR other citation details if **external detailed step-by-step protocols** are available.	Not Applicable	

Experimental study design and statistics	Information included in the manuscript?	In which section is the information available? (Reagents and Tools Table, Materials and Methods, Figures, Data Availability Section)
Include a statement about **sample size** estimate even if no statistical methods were used.	Not Applicable	
Were any steps taken to minimize the effects of subjective bias when allocating animals/samples to treatment (e.g. **randomization procedure**)? If yes, have they been described?	Not Applicable	
Include a statement about **blinding** even if no blinding was done.	Yes	Material and Methods
Describe **inclusion/exclusion criteria** if samples or animals were excluded from the analysis. Were the criteria pre-established?<br><br>If sample or data points were omitted from analysis, report if this was due to attrition or intentional exclusion and provide justification.	Not Applicable	
For every figure, are **statistical tests** justified as appropriate? Do the data meet the assumptions of the tests (e.g., normal distribution)? Describe any methods used to assess it. Is there an estimate of variation within each group of data? Is the variance similar between the groups that are being statistically compared?	Yes	Material and Methods

Sample definition and in-laboratory replication	Information included in the manuscript?	In which section is the information available? (Reagents and Tools Table, Materials and Methods, Figures, Data Availability Section)
In the figure legends: state number of times the experiment was **replicated** in laboratory.	Not Applicable	
In the figure legends: define whether data describe **technical or biological replicates**.	Not Applicable	

Ethics

Ethics	Information included in the manuscript?	In which section is the information available? (Reagents and Tools Table, Materials and Methods, Figures, Data Availability Section)
Studies involving **human participants**: State details of **authority granting ethics approval** (IRB or equivalent committee(s), provide reference number for approval.	Not Applicable	
Studies involving **human participants**: Include a statement confirming that **informed consent** was obtained from all subjects and that the experiments conformed to the principles set out in the WMA Declaration of Helsinki and the Department of Health and Human Services Belmont Report.	Not Applicable	
Studies involving **human participants:** For publication of **patient photos**, include a statement confirming that consent to publish was obtained.	Not Applicable	
Studies involving experimental **animals**: State details of **authority granting ethics approva**l (IRB or equivalent committee(s), provide reference number for approval. Include a statement of compliance with ethical regulations.	Yes	Material and Methods
Studies involving **specimen and field samples:** State if relevant **permits** obtained, provide details of authority approving study; if none were required, explain why.	Not Applicable	

Dual Use Research of Concern (DURC)	Information included in the manuscript?	In which section is the information available? (Reagents and Tools Table, Materials and Methods, Figures, Data Availability Section)
Could your study fall under dual use research restrictions? Please check biosecurity documents and list of **select agents and toxins** (CDC): https://www.selectagents.gov/sat/list.htm	Not Applicable	
If you used a select agent, is the security level of the lab appropriate and reported in the manuscript?	Not Applicable	
If a study is subject to dual use research of concern regulations, is the name of the **authority granting approval and reference number** for the regulatory approval provided in the manuscript?	Not Applicable	

Reporting

The MDAR framework recommends adoption of discipline-specific guidelines, established and endorsed through community initiatives. Journals have their own policy about requiring specific guidelines and recommendations to complement MDAR.

Adherence to community standards	Information included in the manuscript?	In which section is the information available? (Reagents and Tools Table, Materials and Methods, Figures, Data Availability Section)
State if relevant guidelines or checklists (e.g., **ICMJE, MIBBI, ARRIVE, PRISMA**) have been followed or provided.	Not Applicable	
For **tumor marker prognostic studies,** we recommend that you follow the **REMARK** reporting guidelines (see link list at top right). See author guidelines, under 'Reporting Guidelines'. Please confirm you have followed these guidelines.	Not Applicable	
For **phase II and III randomized controlled trials**, please refer to the **CONSORT** flow diagram (see link list at top right) and submit the CONSORT checklist (see link list at top right) with your submission. See author guidelines, under 'Reporting Guidelines'. Please confirm you have submitted this list.	Not Applicable	

Data Availability

Data availability	Information included in the manuscript?	In which section is the information available? (Reagents and Tools Table, Materials and Methods, Figures, Data Availability Section)
Have **primary datasets** been deposited according to the journal's guidelines (see 'Data Deposition' section) and the respective accession numbers provided in the Data Availability Section?	Yes	Data Availibility
Were **human clinical and genomic datasets** deposited in a public access-controlled repository in accordance to ethical obligations to the patients and to the applicable consent agreement?	Not Applicable	
Are **computational models** that are central and integral to a study available without restrictions in a machine-readable form? Were the relevant accession numbers or links provided?	Yes	Data Availibility
If publicly available data were reused, provide the respective **data citations in the reference list**.	Not Applicable	
